# Risk of Inflammatory Bowel Disease Following Hospital‐Treated Infections and Modulatory Role of Host Genetics to Support a Multi‐Hit Pathogenesis Model

**DOI:** 10.1002/advs.76509

**Published:** 2026-07-13

**Authors:** Haiming Zhuang, Lintao Dan, Xin Xiang, Xixian Ruan, Shuai Yuan, Jialu Yao, Jiawei Geng, Jonas F. Ludvigsson, Tian Fu, Candida Abreu, Laurent Peyrin‐Biroulet, Xue Li, Yi Xiao, Fernando Magro, Xiaoyan Wang, Jing Sun, Jie Chen

**Affiliations:** ^1^ Department of Gastroenterology Ruijin Hospital Shanghai Jiao Tong University School of Medicine Shanghai P. R. China; ^2^ Department of Gastroenterology The Third Xiangya Hospital of Central South University Changsha P. R. China; ^3^ Xiangya School of Public Health Central South University Changsha P. R. China; ^4^ Department of Surgery Perelman School of Medicine, University of Pennsylvania Philadelphia Pennsylvania USA; ^5^ Department of Medical Epidemiology and Biostatistics Karolinska Institutet Stockholm Sweden; ^6^ Department of Big Data in Health Science School of Public Health and The Second Affiliated Hospital Zhejiang University School of Medicine Hangzhou Zhejiang P. R. China; ^7^ Unit of Cardiovascular and Nutritional Epidemiology Institute of Environmental Medicine, Karolinska Institutet Stockholm Sweden; ^8^ Department of Paediatrics Örebro University Hospital Örebro Sweden; ^9^ Department of Gastroenterology Affiliated Hangzhou First People's Hospital, School of Medicine, Westlake University Hangzhou P. R. China; ^10^ Department of Infectious Diseases, Faculty of Medicine, Centro Hospitalar S. João, Nephrology Research and Development Unit University of Porto Porto Portugal; ^11^ Department of Gastroenterology Université De Lorraine CHRU Nancy France; ^12^ Department of Dermatology Xiangya Hospital Central South University Changsha Hunan P. R. China; ^13^ CINTESIS@RISE Department Faculdade De Medicina da Universidade do Porto Porto Portugal; ^14^ Postdoctoral Station of Clinical Medicine The Third Xiangya Hospital of Central South University Changsha P. R. China

**Keywords:** genetic susceptibility, infection, inflammatory bowel disease

## Abstract

Infectious diseases can cause lasting immune disturbances, but whether they contribute to later inflammatory bowel disease (IBD) is unclear. Hospital‐treated infections may be especially informative because they reflect substantial immune challenge, yet their relation to IBD risk and the role of host genetics remain poorly defined. It examines hospital‐treated infections and incident IBD in a prospective cohort and integrates gene‐environment interaction analyses to identify susceptibility pathways and develop a post‐infection risk score. Hospital‐treated infections of multiple pathogen types and sites were associated with higher subsequent IBD risk. This association is stronger in carriers of immune‐related risk variants, with Crohn's disease linked mainly to innate immune and autophagy pathways and ulcerative colitis to JAK‐STAT, T‐cell differentiation, and chemokine signaling. An Infection IBD Score based on 44 immune‐related genes stratified post‐infection risk. These findings support infections as triggers of IBD in genetically susceptible individuals and highlight a potential tool for risk stratification.

## Graphical abstract

Hospital‐treated infections are linked to increased risk of inflammatory bowel disease in a large prospective cohort. This risk differs by infection burden, time since infection, and host immune‐genetic susceptibility. The findings support a multi‐hit pathogenesis model in which severe infection and inherited susceptibility jointly shape inflammatory bowel disease development.

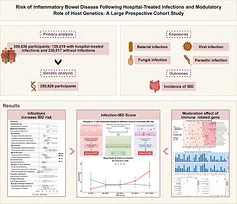

## 1 Introduction

Inflammatory bowel disease (IBD), encompassing Crohn's disease (CD) and ulcerative colitis (UC), comprises a group of immune‐mediated chronic disorders characterized by recurrent gastrointestinal inflammation and sustained disruption of intestinal homeostasis [1, 2]. Although genome‐wide association studies (GWAS) have identified more than 320 genetic loci associated with IBD susceptibility, these variants collectively explain only approximately 10% of the disease heritability [3]. This substantial “missing heritability” underscores the critical role of non‐genetic factors, particularly environmental exposures, in IBD pathogenesis.

Among environmental exposures, infection is one of the most commonly investigated and exerts profound effects on human health [4]. Due to the heterogeneous spatiotemporal distribution of pathogens, individuals are repeatedly exposed to diverse infectious agents throughout life [5]. Increasing evidence indicates that infections not only induce organ injury associated with the acute phase but also trigger long‐lasting immune perturbations, thereby increasing susceptibility to immune‐mediated diseases [6, 7, 8]. This issue has become increasingly prominent with the rising risk of global pandemics and the emergence of novel pathogens. For example, during the recent COVID‐19 pandemic, approximately 43.9% of the global population experienced infection at least once [9]. Consequently, infections have been recognized as important contributors to IBD pathogenesis, warranting focused investigation into their specific pathogenic roles [10].

A limited number of epidemiological studies have suggested a potential link between infections and increased incidence of IBD [11, 12, 13]. Available evidence has focused on specific types of infections, with large‐scale prospective data on overall infections still lacking [14, 15, 16]. This gap hampers the establishment of a clear temporal relationship between infection exposure and subsequent disease onset. Therefore, it is difficult to distinguish whether infection represents a true pathogenic trigger of IBD or merely a secondary consequence [17, 18]. Against the backdrop of ongoing global infectious threats, clarifying this causal relationship is of critical importance for both clinical practice and public health [4].

In healthy individuals, the immune system maintains a delicate balance between tolerance and immune activation. The equilibrium between tolerance and immune activation is disrupted in IBD: barrier defects or innate immune dysfunction heighten susceptibility to pathogen‐driven inflammation, while impaired regulation triggers excessive activation against commensals. For example, *Nod2‐*, *Cybb‐*, or *Atg16l1‐*deficient mice are more prone to opportunistic pathogens and inflammation, and IL10R‐deficient mice develop colitis only after microbial colonization [19, 20, 21, 22]. Effective risk prediction and targeted prevention of IBD require the identification of critical environmental triggers and core gene‐modulated pathogenic pathways [23, 24]. However, systematic investigations into the interplay between infection and host genetic susceptibility remain limited [25, 26].

To address these critical gaps, this study leverages large‐scale phenotypic and genetic data from the UK Biobank prospective cohort to achieve the following objectives: 1) To quantify the association between hospital‐treated infection history and incident IBD (CD and UC); 2) To investigate the modulatory role of immune‐related genes on the link between infection exposure and IBD susceptibility; 3) To develop an Infection IBD Score (IIS), which integrates immune‐related genetic variants for risk stratification and to evaluate its performance.

## 2 Results

### 2.1 Characteristics of the Study Population

The analytic cohort included 359 636 participants, of whom 129 619 had experienced at least one hospital‐treated infection (Figure 1). Among infection subtypes, bacterial infections were the most common that had ever occurred in these individuals (*n* =  119 598; 92.27%), followed by viral (*n* =  16 060; 12.39%), fungal (*n* =  7732; 5.97%), and parasitic infections (*n* =  830; 0.64%) (Table S2).

**FIGURE 1:**
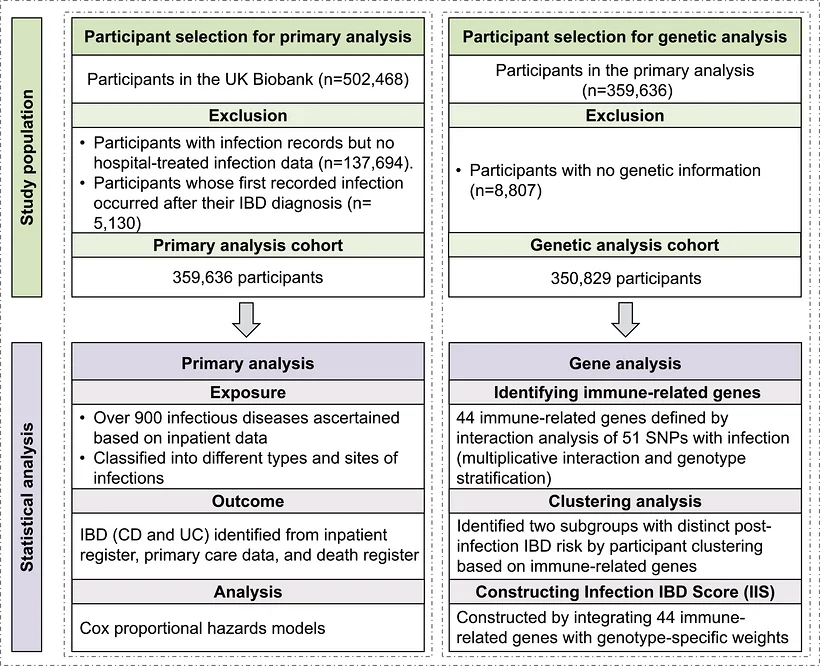
Flowchart of the study.

Compared with participants without hospital‐treated infections, those with such a history were generally older, had higher BMI and TDI, and were more likely to be male, current or former smokers, and physically inactive. They were also less likely to have attained a college or university‐level education (Table 1).

**TABLE 1:** Baseline Characteristics of Participants Based on Infection Exposure Status.

Characteristic	All participants [N = 359 636]	No infection [N = 230 017]	Infection [N = 129 619]	*p* value
**Age (mean (SD))**	57.06 (8.11)	56.24 (8.06)	58.52 (8.00)	<0.001
**Sex (%)**				<0.001
Female	194 066 (54.0)	126 320 (54.9)	67 746 (52.3)	
Male	165 570 (46.0)	103 697 (45.1)	61 873 (47.7)	
**BMI (mean (SD))**	27.47 (4.82)	27.00 (4.50)	28.29 (5.26)	<0.001
**INFLA score (mean (SD))**	−0.04 (6.09)	−0.53 (5.98)	0.83 (6.19)	<0.001
**TDI (mean (SD))**	−1.25 (3.13)	−1.43 (3.04)	−0.94 (3.27)	<0.001
**Ethnic (%)**				0.026
White	339 404 (94.4)	217 225 (94.4)	122 179 (94.3)	
Other	20 232 (5.6)	12 792 (5.6)	7440 (5.7)	
**Education (%)**				<0.001
College degree and above	115 187 (32.0)	80 936 (35.2)	34 251 (26.4)	
No college	244 449 (68.0)	149 081 (64.8)	95 368 (73.6)	
**Alcohol intake (%)**				<0.001
Never	16 222 (4.5)	9224 (4.0)	6998 (5.4)	
Previous	13 199 (3.7)	6637 (2.9)	6562 (5.1)	
Current	330 215 (91.8)	214 156 (93.1)	116 059 (89.5)	
**Smoking status (%)**				<0.001
Never	197,275 (54.9)	133,144 (57.9)	64,131 (49.5)	
Previous	123,718 (34.4)	75,764 (32.9)	47,954 (37.0)	
Current	38,643 (10.7)	21,109 (9.2)	17,534 (13.5)	
**Physical activity (%)**				<0.001
Irregular	111,098 (30.9)	65,922 (28.7)	45,176 (34.9)	
Regular	248,538 (69.1)	164,095 (71.3)	84,443 (65.1)	

SD, standard deviation; BMI, body mass index; INFLA score, low‐grade inflammation score; TDI, Townsend deprivation index.

### 2.2 Hospital‐Treated Infectious Diseases and Risk of Incident IBD, CD, UC Outcomes

During 4,363,921 person‐years of follow‐up, a total of 2,074 incident cases of IBD were identified among the participants. In fully adjusted models, hospital‐treated infection was associated with a markedly increased risk of incident IBD (aHR, 3.44; 95% CI, 3.14–3.77). When stratified by IBD subtype, the association was stronger for CD (aHR, 5.08; 95% CI, 4.35–5.94) than for UC (aHR, 3.34; 95% CI, 3.01–3.72) (*P*‐heterogeneity <0.001) (Figure 2). Similar associations were observed after excluding the first 2–3 years of follow‐up and remained evident after excluding the first 10 years, although with an attenuated effect estimate (aHR, 1.68; 95% CI, 1.35–2.10) (Table S3). A dose–response relationship was observed between infection burden and the risks of IBD, CD, and UC (Table S4). Infections at 7 anatomical sites, including neurological and eye, upper respiratory tract, gastrointestinal, genitourinary, skin and soft tissue, lower respiratory tract, bloodstream, and other infections were all significantly associated with increased risks of IBD, CD, and UC. Notably, gastrointestinal infection demonstrated the strongest associations, with adjusted HR of 5.55 (95% CI, 5.06–6.09) for IBD, 8.37 (95% CI, 7.24–9.68) for CD, and 5.18 (95% CI, 4.64–5.77) for UC (*P*‐heterogeneity < 0.001) (Figure S1). Importantly, after excluding gastrointestinal infections, the associations between non‐gastrointestinal systemic infections and IBD risk remained statistically significant, with adjusted HRs of 1.65 (95% CI, 1.48–1.84) for IBD, 2.10 (95% CI, 1.74–2.54) for CD, and 1.66 (95% CI, 1.46–1.89) for UC, indicating that the observed excess risk was not solely driven by gastrointestinal infections (Table S5).

**FIGURE 2:**
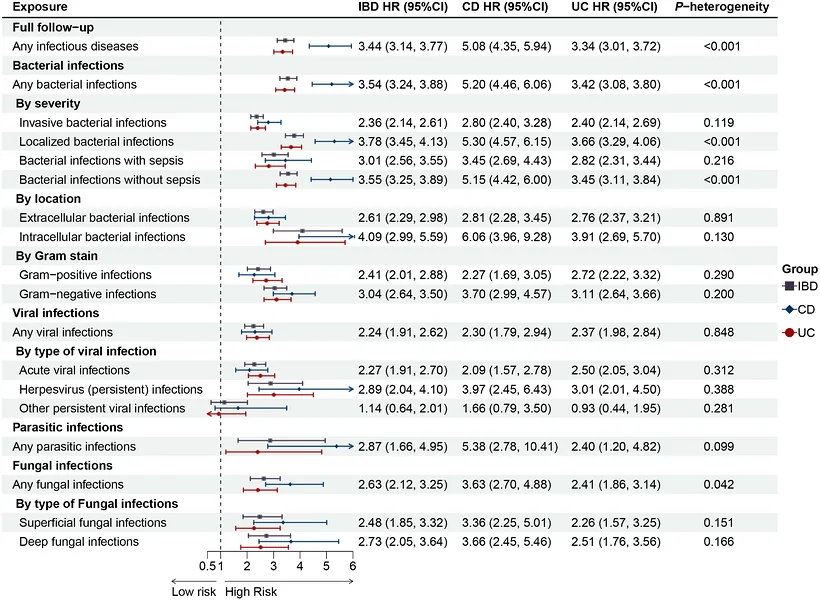
Association between a history of infection and incident IBD, including CD and UC.

In the Level 2 classification by pathogen type, hospital‐treated infections were significantly associated with increased risks of IBD across bacterial (aHR 3.54; 95% CI, 3.24–3.88), viral (aHR 2.24; 95% CI, 1.91–2.62), fungal (aHR 2.63; 95% CI, 2.12–3.25), and parasitic (aHR 2.87; 95% CI, 1.66–4.95) infections. Evidence of heterogeneity between CD and UC was observed for bacterial (*P*‐heterogeneity < 0.001) and fungal (*P*‐heterogeneity = 0.042) infections, but not for viral (*p* = 0.848) or parasitic (*p* = 0.099) infections (Figure 2).

In the Level 3 classification by infection characteristics, all examined bacterial subtypes (intracellular vs. extracellular, Gram‐positive vs. Gram‐negative, invasive vs. localized, and with or without sepsis) and most viral subtypes and fungal infections were significantly associated with increased risks of IBD. Significant heterogeneity between CD and UC was observed for localized bacterial infections (*P*‐heterogeneity < 0.001) and bacterial infections without sepsis (*P*‐heterogeneity < 0.001), whereas no such heterogeneity was detected across viral or fungal subtypes (Figure 2).

Specific‐pathogen analyses showed that intestinal *Clostridioides difficile* and *Campylobacter* infections were significantly associated with increased risks of IBD, CD, and UC, and within fungal infections, *Candida urethritis* and *oral candidiasis* showed similar associations. No significant heterogeneity was observed between CD and UC for any of the specific pathogens (Figure S1).

### 2.3 Modulating Effect of Immune‐Related Genes Implicated in IBD

Recognizing that host gene mutations may modulate the impact of infection on IBD risk, we investigated the modulatory role of immune‐related genes in infection‐related IBD susceptibility (Figure 3A and Figure S2). A total of 44 immune‐related genes with pathogen‐modulatory functions were identified, including 31 in IBD, 29 in CD, and 37 in UC. Based on the strength and consistency of these immune‐related genes, 31 genes implicated in IBD were classified into three evidence tiers. There were 11 genes (*TAB2*, *CCL20*, *CSF2RB*, *IFNG*, *IL18R1*, *IRGM*, *ELMO1*, *IL12B*, *IRF5*, *HLA‐DQA1*, *STAT3*) showed strong and consistent pathogen‐related modulation across multiple pathogens and were designated as high evidence, 14 moderate evidence genes (e.g., *CCL7*, *NOD2*, *IL7R*) exhibited less consistent but still significant modulatory effects, while 6 low evidence genes (e.g., *NFKBIA*, *IL23R*, *IFNGR2*) displayed weak or inconsistent modulation (Figure 3B). Importantly, 14 of the 31 prioritized genes (45.2%) showed attenuated or even directionally opposite associations relative to those reflected in the conventional PRS after infection, rather than uniformly risk‐enhancing effects. This pattern was exemplified by loci such as STAT3 and TAB2 (Table S6). Using the same criteria for disease‐specific analyses, 5 CD‐associated genes (*ELMO1*, *IL18R1*, *MAP3K8*, *SERINC5*, *TAB2*) were classified as high evidence, 16 (e.g., *CCL20*, *CCL7*, *CD40*, *IFNG*) as moderate evidence, and 8 (e.g., *CD226, IFNGR2*, *IL23R*, *IRF6*) as low evidence (Figure 3C). Notably, 13 of these 29 CD‐associated genes (44.8%) showed attenuated or even directionally opposite associations relative to those reflected in the conventional PRS after infection (Table S7). For UC, 7 genes (*CCL20*, *CSF2RB*, *IL12B*, *IL27*, *IRGM*, *NFKBIA*, *STAT3*) were classified as high evidence, 24 (e.g., *ATG5*, *CCL7*, *CD226*, *FCGR2A*) as moderate evidence, and 6 (e.g., *CXCL5*, *IL23R*, *IRF6*) as low evidence (Figure 3D). Similarly, 17 of the 37 UC‐associated genes (45.9%) displayed attenuated or directionally opposite associations compared with the conventional PRS (Table S8).

**FIGURE 3:**
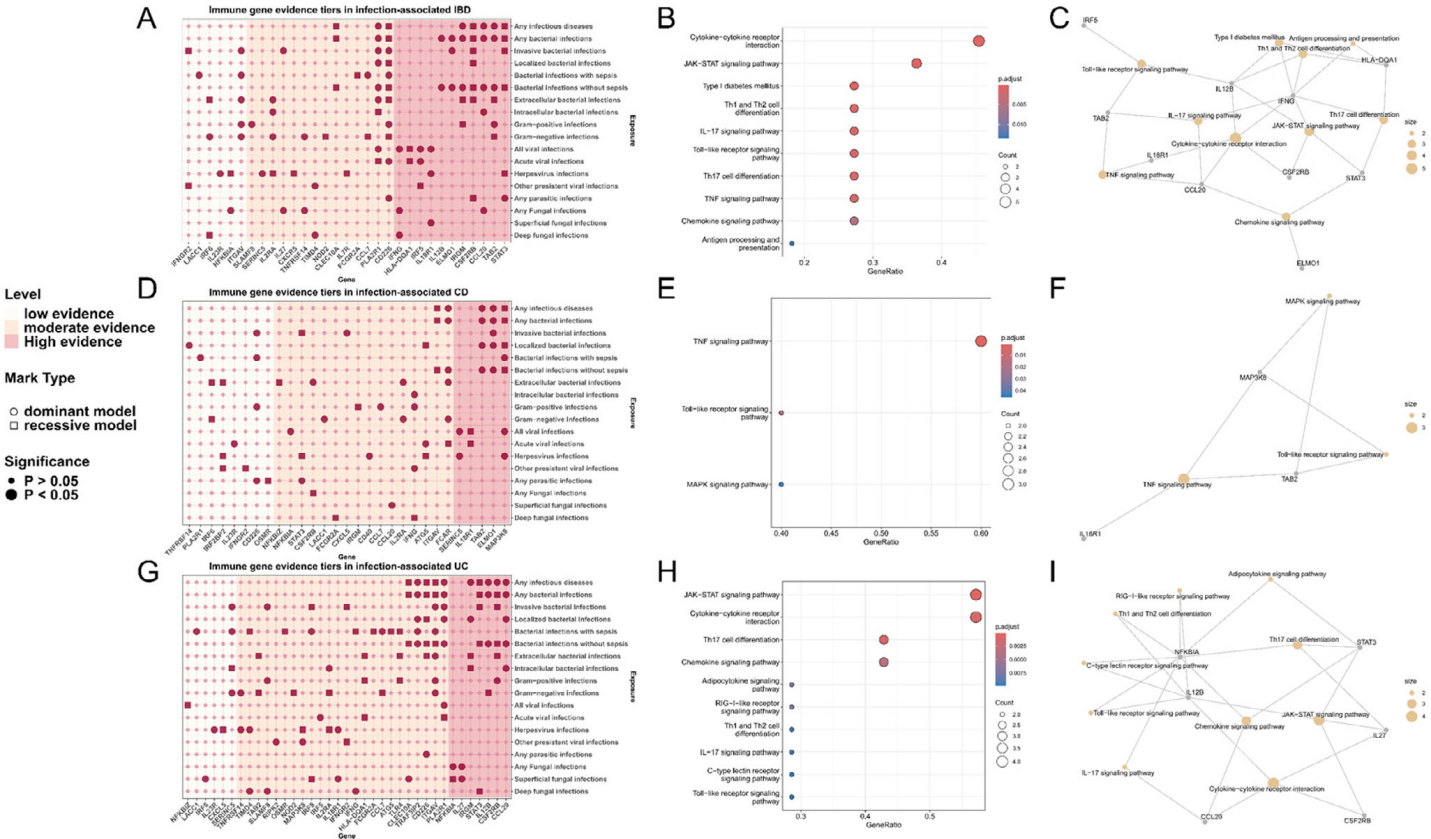
Prioritization and functional characterization of immune‐related genes underlying infection‐associated IBD risk. (A) Overview of the workflow used to prioritize immune‐related genetic loci implicated in infection‐associated susceptibility to IBD. (B–D) Evidence‐based classification and KEGG enrichment of infection‐associated immune‐related genes in IBD, CD, and UC.

Functional enrichment analysis demonstrated that immune‐related genes influencing infection‐related IBD risk were predominantly involved in immune responses and inflammatory signaling, particularly cytokine signaling and T cell differentiation pathways (Figure 3B; Tables S9 and S10). Immune‐related genes in CD and UC, however, exhibited distinct functional profiles: in CD, genes were enriched mainly in innate immune activation and inflammatory pathways, such as TNF, Toll‐like receptor, and MAPK signaling (Figure 3C); whereas in UC, genes were implicated in a broader spectrum of immune and metabolic processes, including JAK–STAT signaling, T cell differentiation, chemokine signaling, and adipocytokine pathways (Figure 3D).

### 2.4 Clustering Analysis of Immune‐Related Genes Implicated in IBD

To further explore the modulatory role of immune‐related genes in post‐infection IBD risk, clustering analyses were conducted based on 31, 29, and 37 immune‐related genes implicated in IBD, CD, and UC, respectively (Tables S11 and S12). Two participant clusters were identified in each analysis, defined by variants modifying the associations between infection and disease risk (Figure 4 and Figure S3). To characterize cross‐disease concordance of the cluster‐enriched gene profiles, we further assessed pairwise Jaccard similarity across IBD, CD, and UC clusters (Figure S4A). This analysis supported two recurrent immune‐genetic profiles across disease definitions. The Cluster 1 was shared across IBD, CD, and UC and included CD226, FCGR2A, IFNGR2, IL23R, IL2RA, and PLA2R1, whereas the Cluster 2 included IL18R1, LACC1, SERINC5, STAT3, and TNFRSF14 (Figure S4B).

**FIGURE 4:**
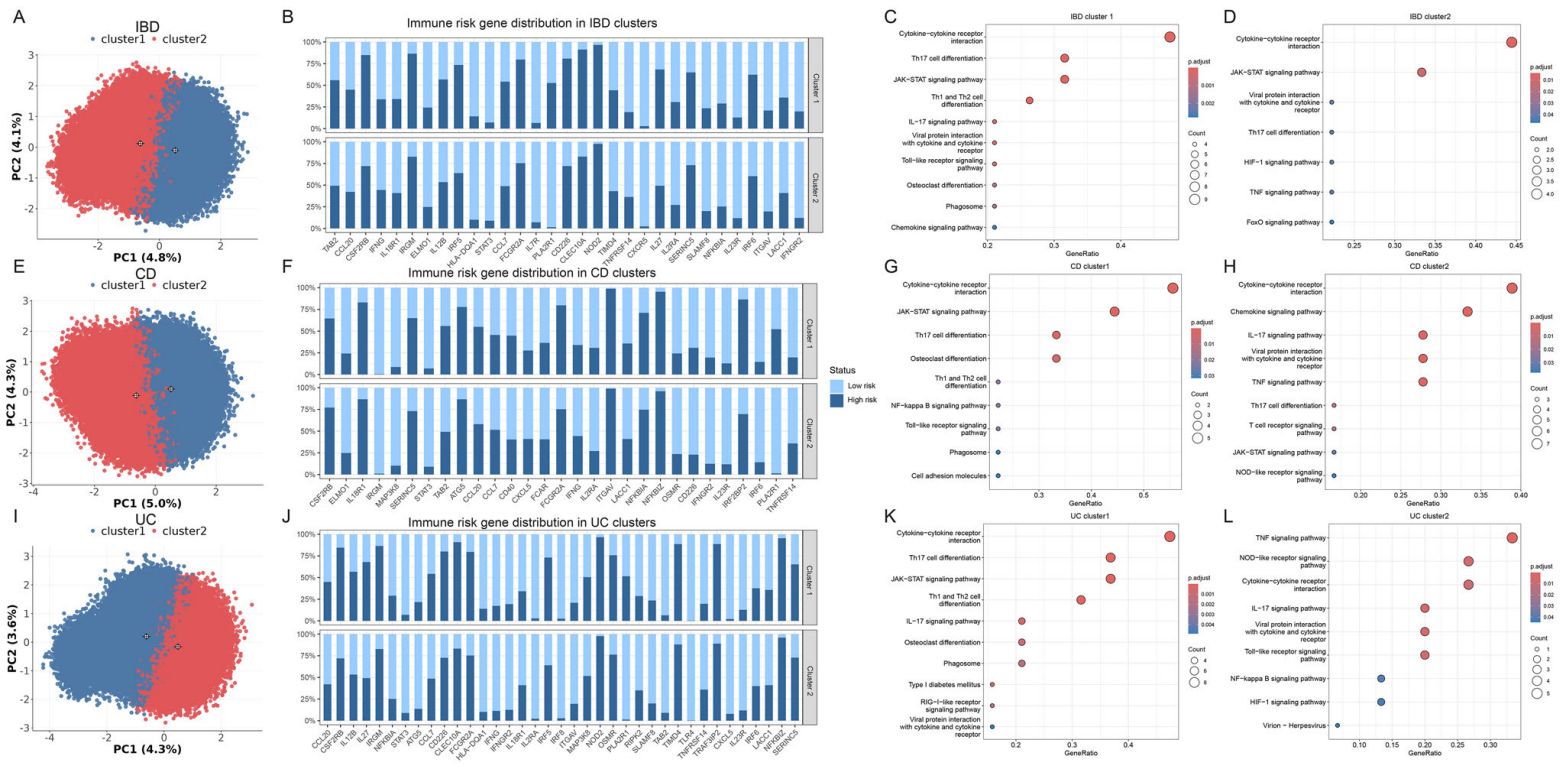
Cluster‐specific distributions of immune‐related genes and KEGG pathway enrichment in infection‐associated IBD, CD, and UC. (A, E, I) Principal component analysis (PCA) identified two distinct clusters in IBD, CD, and UC. (B, F, J) Distribution of immune‐related genes across genetic clusters in IBD, CD, and UC, respectively. (C, G, K) KEGG pathway enrichment analyses of immune‐related genes in Cluster 1 of IBD, CD, and UC, respectively. (D, H, L) KEGG pathway enrichment analyses of immune‐related genes in Cluster 2 of IBD, CD, and UC, respectively.

In CD‐specific, Cluster 1 exhibited a higher proportion of risk variants in genes including *PLA2R1*, *IRF2BP2*, *CD226*, *IFNGR2*, and *TAB2*, with functional profiling revealing significant enrichment in JAK–STAT signaling, Th17 and Th1/Th2 cell differentiation. In contrast, Cluster 2, defined by variants in *TNFRSF14*, *CXCL5*, *CSF2RB*, *IFNG*, and *ATG5*, was more prominently associated with chemokine signaling, IL‐17–mediated inflammation, TNF signaling, as well as T‐cell receptor and NOD‐like receptor pathways. Notably, both clusters shared variants in *MAP3K8*, *IRGM*, *NFKBIZ*, *ELMO1*, and *ITGAV*, converging on common mechanisms including cytokine–cytokine receptor interaction, IL‐17 signaling, and innate immune pathways such as Toll‐like receptor and MAPK signaling, underscoring a foundational role of innate and inflammatory activation in CD (Figure 4D–F and Table S13).

In the UC‐specific analysis, Cluster 1 was primarily characterized by variants in *PLA2R1*, *IL27*, *CSF2RB*, *IRF5*, and *ATG5*. This cluster was functionally enriched in pathways related to Th17 cell differentiation, JAK–STAT signaling, Th1/Th2 differentiation, and RIG‐I–like receptor signaling, highlighting a coordinated adaptive immune. While Cluster 2, containing *TNFRSF14*, *SERINC5*, *IL18R1*, *RIPK2*, and *CXCL5*, also contributed to TNF signaling, NOD‐like receptor signaling, Toll‐like receptor activation, NF‐κB, HIF‐1, and viral protein–cytokine interactions. Both clusters commonly featured *NFKBIZ*, *IRF8*, *TRAF3IP2*, *TLR4*, and *IL2RA* variants, consistently implicating cytokine interaction and chemokine signaling pathways, thereby reflecting convergent inflammatory mechanisms amid distinct cluster‐specific prioritizations (Figure 4G–I and Figure S5, Table S14).

### 2.5 Risk Stratification Based on IIS

To enable genetic risk stratification for post‐infection IBD development, we compared two approaches: the established PRS (constructed based on all recognized risk loci of IBD from previous GWAS) and the IIS. While the PRS failed to stratify IBD risk following infection exposure (Table S15), the IIS demonstrated better discriminative capacity. Individuals with high IIS had substantially higher post‐infection IBD risk than those with low IIS (aHR: 3.15 (95% CI: 2.86–3.48) vs. 6.35 (95% CI: 4.83–8.35); *P* interaction < 0.001), with significant effect modification observed for both CD (*P* interaction = 0.021) and UC (*P* interaction < 0.001) (Figure 5A and Table S16). In absolute risk analyses, participants with high IIS consistently had a greater post‐infection absolute risk than those with low IIS; at 12 years, this corresponded to 11.9 versus 8.1 excess IBD cases per 1,000 individuals (Table S17). Pathogen‐specific IISs for bacterial, viral, and fungal infections also significantly stratified post‐infection IBD risk (All *P* for interaction < 0.05), whereas no stratification was observed for PRS (Tables S19–S21).

**FIGURE 5:**
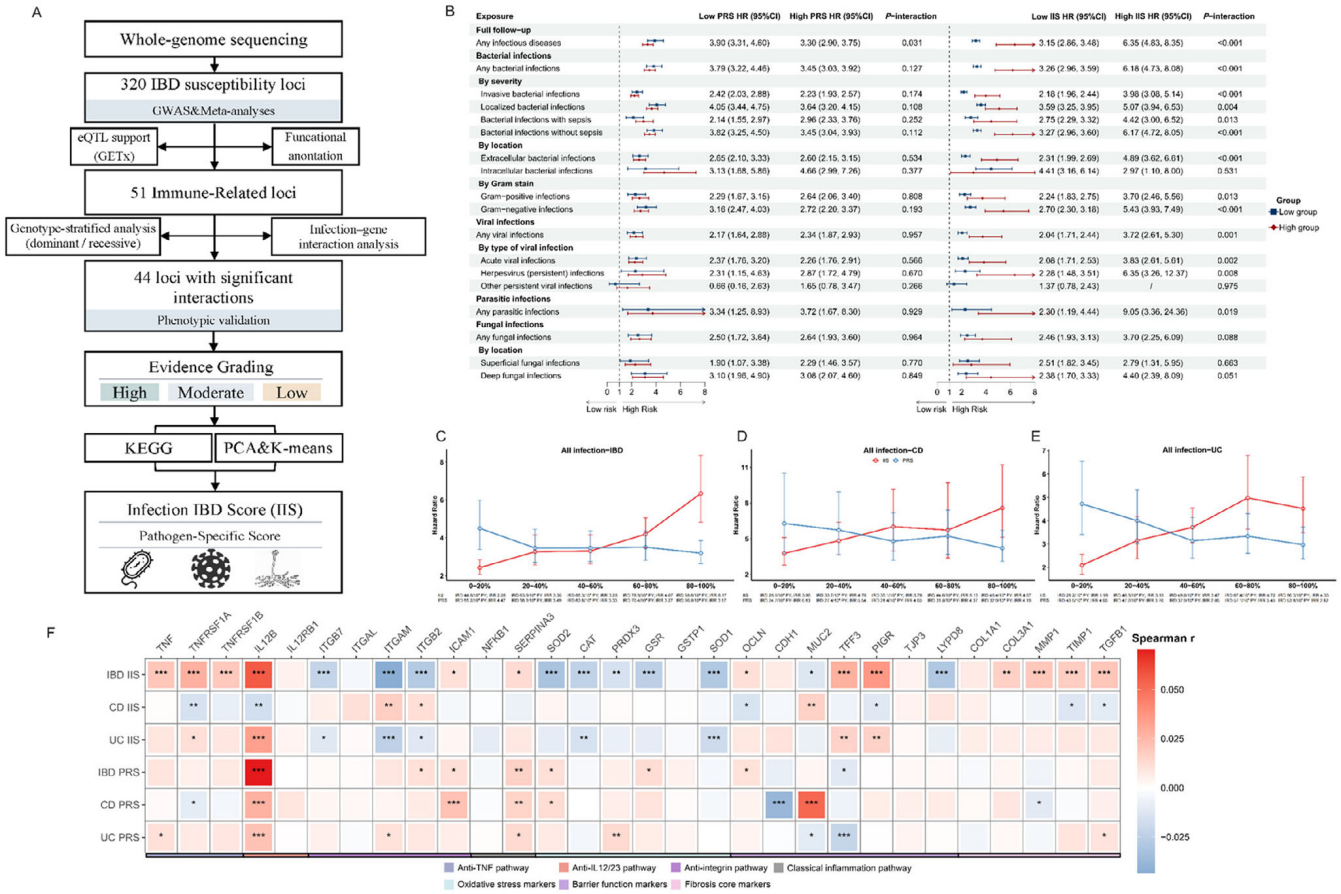
Genetic and proteomic characterization of infection‐associated IBD based on PRS and IIS. (A) Comparison of post‐infection IBD, CD, and UC risks across low‐ and high‐PRS or IIS groups. PRS groups were defined by the median PRS value, whereas IIS groups were defined as IIS ≤0 and IIS >0. (B) Quantile‐based stratification of infection‐associated risk for IBD, CD, and UC. (C) Protein‐level correlations of IIS and PRS in IBD, CD, and UC.

Quantile‐based stratification underscored the divergence between IIS and PRS. IIS quintiles showed a clear dose–response relationship for infection‐related IBD risk: with adjusted HRs increasing progressively across quintiles 0%–<20%: 2.43 (95% CI: 2.07, 2.85), 20%–<40%: 3.27 (95% CI: 2.58–4.16), 40%–<60%: 3.31 (95% CI: 2.63–4.17), 60%–<80%: 4.20 (95% CI: 3.48–5.06), and 80%–100%: 6.35 (95% CI: 4.83–8.35). This increasing trend was also observed in CD and UC (Figure 5B and Table S22). Pathogen‐specific IISs showed similar graded risk patterns, though estimates for viral and fungal IISs did not reach statistical significance, whereas no dose–response relationship was observed for PRS (Table S23). After excluding low‐evidence SNPs, the reduced IIS yielded generally consistent but attenuated risk stratification in both binary and quantile‐based analyses (Figure S6 and Table S24).

To provide biological context for the IIS‐based stratification of post‐infection susceptibility, we examined the correlations between both the IIS and PRS with serum IBD‐related etiological protein profiles (Figure 5C). In the context of IBD, higher IIS was positively correlated with inflammatory mediators, including TNF, TNFRSF1A, TNFRSF1B, IL12B, ICAM1, and SERPINA3, as well as epithelial, barrier, and matrix‐associated proteins, including OCLN, TFF3, PIGR, COL3A1, MMP1, TIMP1, and TGFB1. Conversely, negative correlations were observed with oxidative stress–response enzymes, including SOD2, CAT, PRDX3, GSR, and SOD1, integrins‐related proteins, including ITGB7, ITGAM and ITGB2, and the mucosal defense marker LYPD8. In the subtype analyses, IIS showed distinct correlation patterns between CD and UC. TNFRSF1A, IL12B, OCLN, and PIGR showed opposite associations, being negative in CD but positive in UC. In addition, CD was correlated with higher levels of ITGAM, ITGB2, and MUC2, whereas UC was associated with elevated levels of TFF3 as well as TNFRSF1A and PIGR. These protein‐level associations were broadly consistent with IIS cluster‐enriched pathways related to cytokine signaling, TNF‐mediated inflammation, leukocyte adhesion, epithelial barrier regulation, and tissue remodeling. Such subtype‐specific associations were not observed in the PRS.

### 2.6 Subgroup and Sensitivity Analysis

Subgroup analyses showed no significant effect modification of the infection–IBD association by sex, age, BMI, or smoking overall, though subtype‐specific analyses revealed interactions between certain infections and host factors, suggesting potential heterogeneity (Tables S25–S27). The association between infection and CD varied by Montreal classification, with higher risks observed for stricturing (B2; 10.69, 95% CI: 6.15–18.57, P‐heterogeneity = 0.004), and perianal disease (26.70, 95% CI: 8.15–87.48, P‐heterogeneity = 0.004), compared with non‐stricturing, non‐penetrating disease (4.60, 95% CI: 3.90–5.42) (Table S28). Infection was associated with increased risk across ileal CD, colonic CD, and ileocolonic or unspecified CD. For all infections, the HRs were 3.90 (95% CI: 2.70–5.63) for ileal CD, 4.59 (95% CI: 2.89–7.28) for colonic CD, and 5.53 (95% CI: 4.59–6.66) for ileocolonic or unspecified CD, with no significant heterogeneity across location categories (all P‐heterogeneity >0.05) (Table S29).

We conducted a series of sensitivity analyses, including complete‐case analysis, further adjustment for Charlson Comorbidity index, Low‐grade Inflammation score and baseline antibiotic use, and baseline use of proton pump inhibitors and nonsteroidal anti‐inflammatory drugs, alternative definitions of infection exposure, exclusion of individuals diagnosed with both CD and UC, and restricting the outcome definition to participants with at least two IBD diagnoses to improve diagnostic specificity, all of which yielded results consistent with the main analysis (Tables S30–S36). The association between infection and IBD risk attenuated over time since infection but remained statistically significant, with aHRs of 8.71 (95% CI: 6.61–11.47) within 1 year, 3.58 (95% CI: 3.03–4.23) at 2–5 years, and 2.21 (95% CI: 1.94–2.51) beyond 5 years after infection (Tables S37–S39). Notably, the association remained robust in a competing risks model accounting for death (aHR: 3.76; 95% CI: 3.46–4.08) (Figure S7 and Table S40), and was comparable when follow‐up was restricted to the pre‐COVID‐19 period (aHR: 3.85; 95% CI: 3.47–4.26) (Table S41). Similar associations were observed for both CD and UC. To assess the potential impact of unmeasured confounding, we calculated E‐values for the main infection–IBD associations. For any hospital‐treated infection, the E‐values were 6.34 for IBD, 9.63 for CD, and 6.14 for UC; the corresponding E‐values for the lower confidence limits closest to the null were 5.73, 8.17, and 5.47, respectively. These findings suggest that substantial unmeasured confounding would be required to fully explain the observed associations (Table S42).

## 3 Discussion

In this large‐scale prospective cohort study, we observed a consistent association between hospital‐treated infections and the subsequent risk of IBD. This association was evident for both CD and UC and remained stable across a range of infection characteristics. In addition, we identified 44 immune‐related genes involved in host–pathogen interactions, including 31 associated with IBD, 29 with CD, and 37 with UC. Functional profiling indicated that these genes were primarily involved in immune and inflammatory pathways, particularly cytokine signaling and T cell differentiation. Notably, pathways related to innate immune activation showed stronger involvement in CD, whereas pathways spanning broader immune regulation and metabolic processes appeared more prominent in UC. Integration of these genes into the IIS yielded a validated risk stratification tool that markedly outperformed the conventional PRS with biological plausibility supported by protein profiles. Compared with the low‐IIS group, individuals in the high‐IIS group had more than a two‐fold higher risk of IBD following infection, underscoring both the modulatory role of host genetics and the potential utility of IIS‐guided surveillance in post‐infection individuals.

Previous research on the infection‐IBD interface has primarily focused on opportunistic infections during immunosuppressive treatment [27, 28, 29]. Nevertheless, in the context of the global resurgence of infectious diseases and the emergence of novel pathogens, the potential role of infections to IBD development and progression deserves renewed attention [4, 30]. Our findings align with previous studies showing that gastrointestinal infections, particularly by *Salmonella*, *Campylobacter*, or *Clostridium difficile*, markedly increase subsequent IBD risk [11, 15, 31]. Consistently, a population‐based study in China further demonstrated a bidirectional association between infectious gastroenteritis and IBD [16]. These findings suggest that gastrointestinal infections may be linked to durable disruptions of mucosal homeostasis, and increased susceptibility to chronic intestinal inflammation. For example, an aerolysin‐producing *Aeromonas* variant enriched in ulcerative colitis has been shown to deplete subepithelial macrophages, rendering the colonic mucosa highly vulnerable to inflammatory stimuli [22, 32, 33].

Our findings further suggest that severe infections beyond the gastrointestinal tract may be associated with subsequent IBD in the context of underlying genetic susceptibility and immune dysregulation. We observed a dose‐dependent association between hospital‐treated infections and incident IBD, with higher risk magnitudes for CD than UC. These findings align with and extend previous reports, such as a Danish cohort study linking infectious mononucleosis to IBD [34]. Similar associations have been reported in cardiovascular and neurological disorders, where cumulative infection burden is linked to increased long‐term disease risk, potentially through persistent low‐grade inflammation [34, 35]. Mechanistically, systemic infections may induce sustained cytokine cascades, disrupt immunological homeostasis, and predispose susceptible individuals to intestinal inflammation [19]. However, reverse causation cannot be fully excluded. Severe infection may act as a trigger in some individuals, whereas in others it may reflect undiagnosed or preclinical IBD, as disease evolution may begin years before diagnosis and nonspecific manifestations can appear up to 10 years earlier [16, 36, 37]. Nevertheless, the association remained significant after excluding IBD cases diagnosed within 2, 3, 5, and 10 years after infection, suggesting that short‐term diagnostic delay is unlikely to fully explain the observed association. Interestingly, localized bacterial infections were associated with a higher IBD risk than systemic bacterial infections, and bacterial infections without sepsis conferred greater risk than those complicated by sepsis. This pattern may reflect distinct immunological dynamics of local versus systemic infections: localized infections may induce prolonged tissue‐specific immune activation and inflammatory memory, whereas systemic infections may more strongly engage regulatory or exhaustion pathways [38, 39, 40]. Notably, we observed greater infection‐related susceptibility in complicated CD subtypes, including structuring, penetrating, and perianal phenotypes, suggesting that post‐infectious inflammation may preferentially be more closely associated with aggressive disease behaviors.

Our findings highlight the pivotal role of host immune‐related genes in shaping susceptibility to infection‐associated intestinal inflammation. Functional and clustering analyses revealed that these genes are enriched in pathogen‐responsive pathways converging on fundamental processes such as microbial recognition (Toll‐like receptor signaling), cytokine amplification (TNF, IL‐17, JAK‐STAT), and adaptive immune polarization (Th1/Th2/Th17 differentiation). Notably, the enrichment patterns differed by disease subtype, with CD showing greater involvement of innate immune and autophagy‐related pathways, whereas UC exhibited a broader spectrum encompassing both immune and metabolic processes [41]. These findings are consistent with distinct pathogenic trajectories in CD and UC, potentially reflecting differences in epithelial barrier function, microbial clearance, and effector T‐cell programming [42, 43]

Clustering analyses delineated two intrinsic patterns of immune‐related genetic vulnerability, present in both UC and CD. Across the IBD, CD, and UC analyses, several genes were enriched within the same cluster: *FCGR2A*, *IL23R*, *IL2RA*, *IFNGR2*, *CD226*, and *PLA2R1* were shared Cluster 1‐enriched genes, whereas *IL18R1*, *LACC1*, *SERINC5*, *STAT3*, and *TNFRSF14* were shared Cluster 2‐enriched genes. Several of these shared genes have plausible links to the infection–IBD interface, including *FCGR2A* in antibody‐mediated immune responses, *IL23R* in the IL‐23/Th17 axis, *IFNGR2* in antimicrobial IFN‐γ signaling, and *LACC1* in myeloid bacterial handling and intestinal inflammation [44, 45, 46, 47]. Individuals carrying Cluster 1 variants exhibited substantially increased susceptibility to IBD, most notably in UC, following bacterial infections. This pattern is consistent with the central role of epithelial barrier integrity and mucosal immune regulation in UC, where bacterial exposure may amplify inflammation in the setting of genetically mediated differences in microbial sensing, cytokine signaling, and chemokine‐driven leukocyte recruitment [48, 49]. By contrast, CD appears more strongly influenced by alterations characteristic of Cluster 2, including autophagy impairment, defective innate microbial clearance, and transmural immune activation. These processes may require chronic dysbiosis or cumulative insults, reducing the apparent impact of single infection events [50, 51]. Proteomic analyses provided complementary biological support for these gene‐based findings, with innate immune activation and epithelial stress predominating in CD and adaptive immune and epithelial repair responses in UC. Together, these findings suggest that infection‐associated immune dysregulation may arise through both shared immune‐genetic programs and disease‐specific pathways [52].

A critical limitation of conventional PRS is their aggregation of genetic effects without consideration of environmental interactions, thereby limiting their ability to capture context‐dependent susceptibility. To address this limitation, we systematically identified immune‐related genes that showed significant interactions with hospital‐treated infections in IBD, and integrated them into the IIS. This score quantifies cumulative vulnerability to post‐infection IBD susceptibility, thereby extending genetic risk prediction into an environmentally contextualized framework. Individuals in the highest IIS quintile had more than a twofold higher risk of IBD following infection compared with those in the lowest quintile. This pattern supports the view that post‐infection IBD susceptibility reflects not only infectious exposure itself, but also the immune‐genetic background in which that exposure occurs.

The directionally heterogeneous effects of these loci may partly explain why conventional PRS did not capture post‐infection susceptibility in our study. Loci such as STAT3 and TAB2 did not show uniformly risk‐enhancing effects after infection in our study, but instead displayed attenuated or even directionally opposite associations, indicating that the effects of inflammatory signaling loci may shift according to infectious context. Experimental evidence supports this interpretation. In a *Fusobacterium nucleatum*–associated colitis model, intestinal epithelial Stat3 deletion attenuated colitis severity, inflammatory cytokine expression, barrier disruption, and epithelial apoptosis, and pharmacologic inhibition of STAT3 phosphorylation produced similar protective effects [53, 54, 55]. Likewise, ASB1 was shown to stabilize TAB2 and enhance downstream NF‐κB/MAPK signaling, whereas Asb1 deficiency protected mice from LPS‐ and *Salmonella*‐induced inflammatory death and ameliorated DSS colitis with reduced colonic p‐p65 and IL‐6 [56]. Together, these findings provide biological support for the possibility that selected immune‐related loci shape host responses to infection and may influence progression from acute inflammatory perturbation to persistent intestinal inflammation.

Limitations of the present study merit consideration. First, reliance on hospital records may have missed milder or outpatient‐managed infections, leading to potential exposure misclassification. Hospital‐treated infection is also heterogeneous, and UK Biobank first‐occurrence records did not permit reliable assessment of treatment completeness, recurrence, or persistence. Second, although the associations were robust to adjustment for multiple measured covariates and sensitivity analyses, residual confounding cannot be excluded, including from diet, gut microbiota composition, early‐life exposures, and more detailed antibiotic history. Third, despite lag analyses excluding IBD cases diagnosed within the first 2–3 years and within the first 10 years after infection, as well as E‐value analyses, reverse causation cannot be fully ruled out, particularly given the possibility of diagnostic delay, prolonged subclinical IBD, or immune dysfunction preceding formal diagnosis. Fourth, information on infection chronicity or recurrence was not available, precluding assessment of whether repeated or persistent infections contribute to IBD risk. Finally, UK Biobank participants were predominantly middle‐aged to older adults of European ancestry, which may limit generalizability to younger‐onset IBD and ethnically diverse populations.

In this large prospective cohort, hospital‐treated infections were associated with a sustained increase in IBD risk across multiple infection categories, with stronger relative associations for CD than for UC. We identified 44 immune‐related genetic variants that modified post‐infection susceptibility and integrated them into an Infection IBD Score, which stratified post‐infection IBD risk more clearly than a conventional PRS. Together, these findings link population‐level infection risk with host immune‐genetic susceptibility and support a multi‐hit model in which severe infections and immune‐related genetic background jointly contribute to future IBD risk.

## 4 Methods

### 4.1 Study Design and Population

This prospective cohort study was conducted using data from the UK Biobank, a large population‐based resource comprising over 500 000 participants aged 40–69 years at baseline (recruited between 2006 and 2010). Ethical approval was obtained from the North West Multi‐center Research Ethics Committee (21/NW/0157) [57].

To ensure exposure validity and preserve the temporal sequence between infection and incident IBD, we excluded participants with infection records lacking hospital‐treated infection status, those whose first recorded infection occurred after IBD diagnosis, and those with prevalent IBD at baseline.

### 4.2 Exposure Assessment

Hospital‐treated infectious diseases were defined as the primary exposure and were ascertained through linkage to national inpatient health records in the UK. Diagnoses were ascertained based on ICD‐9 and ICD‐10 coding, only hospital‐recorded infections with a valid date of first occurrence were included to ensure high diagnostic specificity [34]. To comprehensively capture the complexity of infectious exposure, participants were classified into three graded levels: Level 1 (overall infection), Level 2 (e.g., bacterial, viral, fungal, and parasitic), and Level 3 (e.g., invasiveness, Gram stain, and severity). These classifications were used to assess variation in infection‐associated IBD risk by pathogen type, anatomical site, invasiveness, sepsis status, bacterial characteristics, viral persistence, fungal depth, and selected pathogen‐level categories. Bacterial infection and fungal infection diagnoses were further classified by pathogen for pathogen‐level analysis (Table S43).

Infections were additionally classified by 9 anatomical system, including neurological and eye, upper respiratory tract, gastrointestinal, genitourinary, skin and soft tissue, lower respiratory tract, bloodstream, bone, heart. Infections that could not be attributed to a specific site were grouped as other infections (Table S44) [35]. The number of cumulative infections was also been counted [34].

### 4.3 Outcome Ascertainment

The primary outcome was incident IBD, including CD and UC. Cases were ascertained through linkage with nationwide inpatient, primary care and death register records within the UK Biobank. CD and UC were defined using ICD‐9 codes 555 and 556, and ICD‐10 codes K50 and K51, respectively [58]. CD subphenotypes, including disease behavior and disease location, were further defined using ICD diagnostic. Previous studies conducted in the UK have demonstrated acceptable diagnostic accuracy when using at least one diagnosis code, with reported accuracies of 87% in inpatient data and 92% in primary care data [59, 60]. As a sensitivity analysis to improve outcome specificity, we restricted the outcome definition to participants with at least two recorded IBD diagnoses.

### 4.4 Covariates

Baseline covariates were prespecified based on their established associations with both infectious exposures and IBD risk. These included age, sex (male, female), ethnicity (white, others), education level (college degree, below college degree), and body mass index (BMI, continuous in kg/m^2^), smoking status (never, former, current), alcohol intake (never, former, current), and physical activity (adequate, inadequate). We included Townsend deprivation index (TDI) as a measure of material deprivation within the population, which was derived from postcodes of participants in the UK Biobank. Missing data were minimal (proportion <3%) and were imputed using mean for continuous variables and mode for categorical variables (Table S45).

### 4.5 Genetic Variables From Whole‐Genome Sequencing Data

To prioritize immune‐related genes involved in infection‐related IBD susceptibility, we compiled a set of 320 genome‐wide significant IBD susceptibility loci from published large‐scale GWAS and GWAS meta‐analyses. These loci were supported by meta‐analytic evidence or replication across at least two independent GWAS, with each locus mapped to a single representative gene and represented by a lead SNP [3]. Variants within these loci were functionally annotated using integrated genomic resources, including NCBI, GeneCards, and the GWAS Catalog. Loci mapped to genes with established roles in host immunity or pathogen defense were prioritized for interaction analyses, with functional support further assessed using eQTL evidence from GTEx Project v8.

For each locus, we used whole‐genome sequencing (WGS) data from the UK Biobank to perform genotype‐stratified analyses and classified associated SNPs under dominant or recessive genetic models based on effect estimate patterns [61]. For each prioritized immune‐related locus, infection–genotype interaction was evaluated using Cox proportional hazards models that included infection status, genotype group, and a multiplicative infection × genotype interaction term, with full covariate adjustment. Because these analyses were conducted within a biologically pre‐specified candidate set of known IBD susceptibility loci annotated to immune or pathogen‐defense functions, loci with two‐sided interaction *p* values <0.05 were prioritized for IIS construction. Loci showing evidence of interaction were further examined for associations with inflammation‐related phenotypes, including circulating markers, leukocyte subsets, and ratio‐based indices, with multiple testing controlled using the Benjamini–Hochberg false discovery rate procedure [62]. Finally, selected loci were categorized by degree of evidence (high, moderate, or low) that supports a modulatory role in infection‐related susceptibility to IBD. High‐evidence loci were defined as those demonstrating both 1) statistically significant and directionally consistent infection‐gene interactions across Level 1 overall infections, Level 2 pathogen classes, and Level 3 pathogen characteristics, and 2) functional support, including biologically plausible annotations related to immune or infection response, with additional support from eQTL evidence where available. Loci meeting only one of these criteria were classified as moderate evidence, whereas those meeting neither criterion were designated as low evidence.

To compare risk stratification of IBD following infection exposure, we additionally constructed polygenic risk scores (PRS) to quantify genetic susceptibility to IBD, CD, and UC, following established protocols [63, 64]. Kyoto Encyclopedia of Genes and Genomes (KEGG) pathway enrichment analysis was then performed to explore the major biological functions of these loci [65]. Clustering analysis based on the 44 loci was further conducted using Principal Component Analysis (PCA) and k‐means, and the resulting groups were used for stratified analyses of post‐infection IBD risk [66]. To assess concordance of cluster‐enriched gene profiles across IBD, CD, and UC, pairwise similarity was further evaluated using the Jaccard index, calculated as the number of shared cluster‐enriched genes divided by the union of genes in each pairwise comparison [67].

To comprehensively integrate the effects of these variants for risk stratification, we subsequently developed an IIS by assigning genotype‐specific weights according to the direction and inheritance pattern of each variant's interaction effect from stratified analyses. Separate IIS were constructed for IBD, CD, and UC using the corresponding outcome‐specific prioritized loci. For loci following a dominant model, the reference homozygote was assigned −1 and the heterozygote/alternative homozygote were assigned +1 when the variant increased risk; the coding was reversed when the variant decreased risk. For loci following a recessive model, the alternative homozygote was assigned +1 and the other genotypes −1 when the variant increased risk, with the coding reversed when the variant decreased risk. Using the same framework, we further derived pathogen‐specific scores for bacterial, viral, and fungal infections.

We used plasma proteomic measurements from the UK Biobank (UKB) Pharma Proteomics Project (UKB‐PPP), in which approximately 54 000 participants had ≈2900 proteins quantified using the Olink Explore PEA platform [68]. This large‐scale dataset provides a comprehensive resource for linking genetic and proteomic signatures of immune regulation. Using this dataset, we curated a panel of IBD‐related proteins grouped into prespecified functional categories and assessed their correlations with the IIS using Spearman correlation analyses (Table S46).

### 4.6 Statistical Analysis

Baseline characteristics were summarized as means with standard deviations (SDs) for continuous variables and as counts with percentages for categorical variables. Follow‐up time was calculated from the date of infection occurrence until the earliest of IBD diagnosis, death, loss to follow‐up, or end of the study period (Hospital Episode Statistics for England in 2022‐10‐31, Scottish Morbidity Record in 2022‐08‐31, Patient Episode Database for Wales in 2022‐03‐31). Infections were modeled as time‐varying exposures.

In the primary analysis, Cox proportional hazards models were used to estimate hazard ratios (HRs) and 95% confidence intervals (CIs) for the associations between hospital‐treated infectious diseases and incident IBD, including both CD and UC. The fully adjusted models accounted for age, sex, ethnicity, educational attainment, TDI, BMI, smoking status, alcohol consumption, and physical activity. Heterogeneity across disease subtypes was calculated with a contrast test method to assess whether the exposure‐disease association differed among the disease subtypes [69].

In secondary analyses, dose–response relationships were evaluated based on infection burden definitions used in previous studies [34]. The cumulative number of distinct infectious disease diagnoses was categorized into four groups: no infection, one infection, two infections, and three or more infections. Because UK Biobank first‐occurrence records provide only the first recorded date for each infectious condition, recurrence, persistence, or subsequent new episodes of the same infection could not be reliably distinguished. Each infectious condition was counted only once when calculating total infection burden. Trend tests were conducted to assess linear associations across infection burden categories.

In genetic variants level analyses, participants were stratified into high/low groups using IBD‐PRS and IIS thresholds. Infection‐genetic interaction effects were assessed via multiplicative interaction p‐values for each scoring system. To compare the predictive performance of IBD‐PRS and IIS for infection‐associated IBD risk, each scoring system was divided into quintiles (0%–<20%, 20%–<40%, 40%–<60%, 60%–<80%, 80%–100%). HRs with 95% CIs were estimated to assess relative risk discrimination between the two systems and dose–response trends across quintiles.

Subgroup analyses were conducted by age (<60 vs. ≥60), sex (female vs. male), BMI (<25 vs. ≥25 kg/m^2^), and smoking status (never vs. previous/current). Additional analyses were conducted according to CD subphenotypes, including disease behavior and disease location. To assess the robustness of our findings, we conducted several sensitivity analyses: 1) complete‐case analysis; 2) additionally adjusting for Charlson Comorbidity Index (CCI), Low‐grade Inflammation score (INFLA score), and baseline antibiotic use, and baseline use of proton pump inhibitors and nonsteroidal anti‐inflammatory drugs (Table S47); 3) excluding IBD cases diagnosed within one, two, and three years following the initial infectious episode; 4) excluding participants with post‐baseline infections and restricting the analysis to pre‐baseline infections to minimize reverse causation; 5) including all infections, encompassing self‐reported and primary care recorded infections in addition to hospital‐treated infections; 6) examining the association across different time intervals since infection (<1 year, 2–5 years, and >5 years); 7) excluding participants with gastrointestinal infections; 8) excluding individuals simultaneously diagnosed with both CD and UC; 9) restricting the outcome definition to participants with at least two recorded IBD diagnoses; 10) applying a competing risk model to account for death as a competing event; and 11) restricting follow‐up to the pre‐COVID‐19 period (before 1 January 2020) to exclude potential effects of the pandemic. Analyses were conducted in R version 4.2.0. Two‐sided *p* values < 0.05 were considered significant.

## Author Contributions

Conceptualization: **X.Y.W**., **J.S**., and **J.C**.; Methodology: **H.M.Z**., **L.T.D**., **X.X**., **J.F.L**., **T.F**., **C.A**., **L.P.B**., **X.L**., and **F.M**.; Data curation: **L.T.D**., **X.X.R**., **S.Y**., **J.L.Y**., and **J.W.G**.; Formal analysis: **L.T.D**., **J.C**., and **X.X.R**.; Investigation: **H.M.Z**., **L.T.D**., and **X.X**.; Writing – original draft: **H.M.Z**., **L.T.D**., and **X.X**.; Writing – review & editing: **H.M.Z**., **L.T.D**., **X.X**., **J.F.L**., **T.F**., **C.A**., **L.P.B**., **X.L**., **Y.X**., **F.M**., **X.Y.W**., **J.S**., and **J.C**.; Supervision: **X.Y.W**., **J.S**., and **J.C**.; Project administration: **X.Y.W**., **J.S**., and **J.C**.

## Funding

The authors are supported by the National Natural Science Foundation of China (82500637, J.C.; U23A20492 and 82170553, X.Y.W.; 82470543 and 82204019, X.L.; 82270575, J.S.), the Natural Science Fund for Excellent Young Scholars of Hunan Province (2025JJ40083, J.C.), the Natural Science Fund for Distinguished Young Scholars of Zhejiang Province (LRG26H260001, X.L.), the Natural Science Foundation of Changsha (kq2502174, J.C.), the National Postdoctoral Program for Innovative Talents of China (GZC20251322, J.C.), the China Postdoctoral Science Foundation (2025M782332, J.C.), the Science Fund for Creative Research Groups of the Natural Science Foundation of Hunan Province (2024JJ1014, X.Y.W.), the Scientific Research Program of FuRong Laboratory (2023SK2085‐3, X.Y.W.), the Qingfeng Scientific Research Fund of the China Crohn's & Colitis Foundation (CCCF‐QF‐2023Z02‐02, X.L.), and Central South University Clinical Research Zhang Xiao‐qian Program (ZXQ2026A06).

## Conflicts of Interest

Laurent Peyrin‐Biroulet has served as a consultant for Abbvie, Abivax, Adacyte, Alimentiv, Alfasigma, Amgen, Apini, Banook, Bristol Myers Squibb (BMS), Celltrion, Enthera, Ferring, Fresenius Kabi, Galapagos, Genentech, Gilead, Iterative Health, Janssen, Lilly, LifeMine, Medac, Morphic, MSD, Nordic Pharma, Novartis, Oncodesign Precision Medicine, ONO Pharma, OSE Immunotherapeutics, Par' Immune, Pfizer, Prometheus, Roche, Roivant, Samsung, Sandoz, Sanofi, Sorriso, Spyre, Takeda, Teva, ThirtyFiveBio, Tillotts, Vectivbio, Vedanta, and Ventyx. He has received lecture fees from Abbvie, Alfasigma, Amgen, Biogen, Celltrion, Ferring, Galapagos, Genentech, Gilead, Iterative Health, Janssen, Lilly, Medac, MSD, Nordic Pharma, Pfizer, Sandoz, Takeda, and Tillotts.

## Supporting information




**Supporting File**: advs76509‐sup‐0001‐SuppMat.docx.

## Data Availability

The data that support the findings of this study are available from UK Biobank. Restrictions apply to the availability of these data, which were used under license for this study. Data are available from https://www.ukbiobank.ac.uk/ with the permission of UK Biobank.
