# Supplementary material for: Risk of Inflammatory Bowel Disease Following Hospital‐Treated Infections and Modulatory Role of Host Genetics to Support a Multi‐Hit Pathogenesis Model
Source: Adv Sci (Weinh). 2026 Jul 13;13(55):e76509. doi: 10.1002/advs.76509 (PMC13359404; doi:10.1002/advs.76509)
Supplement: Supplementary file 1 [file ADVS-13-e76509-s001.docx]

Supporting Information

**Risk of Inflammatory Bowel Disease Following Hospital-Treated Infections and Modulatory Role of Host Genetics to Support a Multi-hit Pathogenesis Model**

Haiming Zhuang†, Lintao Dan†, Xin Xiang†, Xixian Ruan, Shuai Yuan, Jialu Yao, Jiawei Geng, Jonas F Ludvigsson, Tian Fu, Candida Abreu, Laurent Peyrin-Biroulet, Xue Li, Yi Xiao, Fernando Magro, Xiaoyan Wang *, Jing Sun *, Jie Chen *

**This file includes:**

Supplementary Methods

Figs. S1 to S8

Tables S1 to S47

References (1 to 9)

**[Supplementary Methods](#_Toc9260)**	[5](#_Toc9260)

**[Supplementary Figure](#_Toc27853)**	[10](#_Toc27853)

[Figure S1. Associations of (A) detailed infection types and (B) infection locations with incident IBD, including CD and UC.	10](#_Toc14828)

[Figure S2. Associations between immune-related SNPs and inflammation-related laboratory markers.	11](#_Toc31997)

[Figure S3. Elbow plots for determining the optimal number of clusters in k-means clustering of immune-related genetic variants.	13](#_Toc16152)

[Figure S4. Gene-profile similarity of cluster-enriched immune-related genes across IBD, CD, and UC.	14](#_Toc31685)

[Figure S5. Clustering of immune-related SNPs and stratified risk analysis for IBD, CD, and UC.	15](#_Toc4229)

[Figure S6. Quantile-based stratification of post-infection IBD, CD, and UC risk by reduced IIS after excluding low-evidence SNPs.	16](#_Toc20086)

[Figure S7. Cumulative incidence of IBD, CD and UC according to infection type in competing risk models accounting for death as a competing event.	17](#_Toc22595)

[Figure S8. Age-specific associations between infection and risk of IBD, CD, and UC.	18](#_Toc1903)

**[Supplementary Tables](#_Toc3554)**	[19](#_Toc3554)

[Table S1. STROBE Statement—Checklist of items that should be included in reports of cohort studies.	19](#_Toc4497)

[Table S2. Hospital-treated infections: hierarchical classification of infection types and subtypes.	21](#_Toc2205)

[Table S3. Sensitivity analyses of the association between hospital-treated infection and incident IBD, CD, and UC using lag periods of 1, 2, 3, and 10 years after infection.	22](#_Toc1953)

[Table S4. Associations Between Infectious Burden and the Risk of IBD, CD, and UC.	23](#_Toc24268)

[Table S5. Sensitivity analyses of the associations between infection and incident IBD, CD, and UC after exclusion of participants with gastrointestinal infections.	24](#_Toc6806)

[Table S6. Prioritized immune-related SNPs derived from genome-wide significant IBD susceptibility loci and incorporated into the IIS.	27](#_Toc10630)

[Table S7. Prioritized immune-related SNPs derived from genome-wide significant CD susceptibility loci and incorporated into the IIS.	28](#_Toc25408)

[Table S8. Prioritized immune-related SNPs derived from genome-wide significant UC susceptibility loci and incorporated into the IIS.	29](#_Toc14882)

[Table S9. KEGG pathway enrichment analysis according to evidence level of immune-related genes.	31](#_Toc16952)

[Table S10. KEGG pathway enrichment analysis of prioritized immune-related genes implicated in infection-associated IBD, CD, and UC susceptibility.	38](#_Toc30965)

[Table S11. Prioritized immune-related SNPs from genome-wide significant IBD loci for infection-related modulation.	43](#_Toc18486)

[Table S12. Proportion of Genetic Risk in Two Clusters of immune-related Genes in IBD.	45](#_Toc7866)

[Table S13. Proportion of Genetic Risk in Two Clusters of immune-related Genes in CD.	46](#_Toc12871)

[Table S14. Proportion of Genetic Risk in Two Clusters of immune-related Genes in UC.	47](#_Toc18057)

[Table S15. Stratification of post-infection IBD, CD, and UC risk by PRS across infection types.	48](#_Toc8307)

[Table S16. Stratification of post-infection IBD, CD, and UC risk by IIS across infection types.	50](#_Toc1106)

[Table S17. Absolute risks and infection-associated absolute risk differences for IBD, CD, and UC according to binary-based IIS strata.	53](#_Toc8916)

[Table S18. Absolute risks and infection-associated absolute risk differences for IBD, CD, and UC according to binary-based PRS strata.	55](#_Toc19489)

[Table S19. Stratification of post-infection IBD, CD, and UC risk by bacterial-specific IIS.	57](#_Toc22577)

[Table S20. Stratification of post-infection IBD, CD, and UC risk by viral-specific IIS.	58](#_Toc6345)

[Table S21. Stratification of post-infection IBD, CD, and UC risk by fungal-specific IIS.	59](#_Toc29334)

[Table S22. Dose–response relationship between infection types and risk of IBD and subtypes according to IIS and PRS quintiles.	60](#_Toc29863)

[Table S23. Dose–response relationship between infection types and risk of IBD and subtypes according to pathogen-specific IIS and PRS quintiles.	63](#_Toc22909)

[Table S24. Stratification of post-infection IBD, CD, and UC risk by reduced IIS after excluding low-evidence SNPs across infection types.	64](#_Toc23951)

**[Subgroup analysis](#_Toc7820)**	[67](#_Toc7820)

[Table S25. Subgroup analysis for associations of infection with incident of inflammatory bowel disease stratified by age, sex, body mass index, and smoking status.	67](#_Toc1324)

[Table S26. Subgroup analysis for associations of infection with incident of crohn's disease stratified by age, sex, body mass index, and smoking status.	70](#_Toc24819)

[Table S27. Subgroup analysis for associations of infection with incident of ulcerative colitis stratified by age, sex, body mass index, and smoking status.	73](#_Toc14682)

[Table S28. Subgroup analysis for the associations between infection types and CD Montreal phenotypes.	76](#_Toc23780)

Table S29. Associations between infection subtypes and incident CD according to disease location.	77

**[Sensitivity analyses](#_Toc23610)**	[78](#_Toc23610)

Table S30. Sensitivity analysis of the associations between infection and incident IBD, CD, and UC using a complete-case approach.	78

[Table S31. Sensitivity analyses of the associations between infection and incident IBD, CD, and UC after further adjustment for CCI, INFLA score, and baseline use of proton pump inhibitors and nonsteroidal anti-inflammatory drugs..	81](#_Toc4041)

[Table S32. Sensitivity analyses of the associations between infection and incident IBD, CD, and UC with further adjustment for baseline antibiotic use.	84](#_Toc21018)

[Table S33. Sensitivity analysis of the associations between infectious diseases and risk of IBD, CD, and UC after excluding participants with post-baseline infections (N = 270,749).	86](#_Toc24348)

[Table S34. Sensitivity analysis of the associations between infectious diseases and risk of IBD, CD, and UC after including both hospital-treated and self-reported infections (N = 498,107).	89](#_Toc15205)

[Table S35. Sensitivity analyses of the associations between infection and incident IBD, CD, and UC after exclusion of individuals diagnosed with both CD and UC.	92](#_Toc24335)

[Table S36. Sensitivity analyses of the associations between infection and incident IBD, CD, and UC restricting the outcome definition to participants with at least two IBD diagnoses.	95](#_Toc17200)

[Table S37. Sensitivity analyses of the associations between infection and incident IBD by time since infection.	98](#_Toc32615)

[Table S38. Sensitivity analyses of the associations between infection and incident CD by time since infection.	101](#_Toc20260)

[Table S39. Sensitivity analyses of the associations between infection and incident UC by time since infection.	104](#_Toc22580)

[Table S40. Sensitivity analyses assessing the associations between infectious diseases and IBD, CD, and UC accounting for the competing risk of death.	107](#_Toc986)

Table S41. Sensitivity analysis of the associations between infectious diseases and risk of IBD, CD, and UC, with follow-up restricted to the pre–COVID-19 period.	108

Table S42. Sensitivity analysis of the associations between infection and incident IBD, CD, and UC using E-values..	109

[Table S43. Classification of type-specific infectious diseases in the study on diagnosis level.	111](#_Toc23890)

[Table S44. Classification of site-specific infectious diseases in the study on diagnosis level.	118](#_Toc26847)

[Table S45. Definition, classification, and missing rates of major covariates.	120](#_Toc14029)

[Table S46. Comprehensive IBD-related protein panel integrating inflammatory, therapeutic, and barrier-fibrosis pathways.	122](#_Toc4594)

[Table S47. Definition and coding of antibiotic use.	124](#_Toc11687)

Supplementary Methods

**lCD codes assigned for phenotypes of the Montreal Classification**

The Montreal classification for CD phenotypes was determined based on ICD diagnostic and surgical procedure codes. Disease behavior was defined as non-stricturing, non-penetrating (B1), stricturing (B2; K56.5, K56.6, K56.7, or K62.4), penetrating (B3; K63.0, K63.2, K31.6, N82.2, N82.3, N82.4), Perianal disease modifier (P; K60.3, K60.4, K60.5, K61.0, K61.1, K61.2, K61.3, K61.4, K62.4). CD disease location was classified using ICD-10 codes as ileal CD (K50.0), colonic CD (K50.1), and ileocolonic or unspecified CD (K50.8 or K50.9) (1).

**Ascertainment of hospital-treated infectious disease**

We retrieved both primary and secondary diagnoses of hospital-treated diseases from linked hospital admission data from HES APC (Hospital Episode Statistics–Admitted Patient Care [England]), SMR01 (Scottish Morbidity Records–General/Acute Inpatient and Day Case Admissions [Scotland]) and PEDW (Patient Episode Database for Wales), using the International Classification of Diseases, 10th Revision (ICD-10) and ICD-9. For the primary analysis, we classified hospital-treated infectious diseases hierarchically to reflect the type of pathogen and severity of infection. Level 1 includes all infectious diseases—ie, a total of 960 ICD-10 codes. At level 2, infectious diseases are divided into bacterial, viral, parasitic, or fungal infections. At level 3, we further classified bacterial infections to reflect properties of the infection and pathogen: disease invasiveness and severity ([potentially] invasive vs [mostly] localised, and with sepsis vs without sepsis); bacterial location and related adaptive immune responses (extracellular vs obligate or facultative intracellular [extracellular bacteria tend to trigger type 17 T-helper responses, intracellular bacteria and viruses tend to trigger type 1 T-helper responses]); and cell wall structure (Gram-positive vs Gram-negative bacteria vs mycobacteria vs mycoplasma [lipopolysaccharides produced by Gram-negative bacteria strongly stimulate the immune system]). Classifications of bacterial location and cell wall structure were based only on ICD-10 codes that defined the causative microorganism unambiguously. Examples of invasive bacterial infections included appendicitis, pneumonia, and pyelonephritis; and localised bacterial infections included gastroenteritis, tonsillitis, and cystitis. Viral infections were classified into acute infections typically eradicated by the immune system, herpesvirus infections that persist in the body after primary infection, and other persistent viral infections such as HIV. Fungal infections were further classified into superficial fungal infections (eg, dermatophytosis, candidiasis of skin and mucosa), which are generally localized and less life-threatening, and deep fungal infections (eg, aspergillosis, systemic candidiasis), which often involve invasive disease with high morbidity and mortality. This subdivision was made to capture clinically meaningful heterogeneity, as superficial and deep fungal infections differ substantially in pathogenesis, immune response, and prognosis. Mycobacterial, mycoplasma, and parasitic infections were too rare to be analysed separately, but they were included in the analyses of the broader categories of infections to which they belonged (2,3).

To further investigate the role of specific pathogens in the development of IBD, we performed pathogen-level analyses restricted to infections with a sufficient number of cases in the UK Biobank. The selection of pathogens was informed by prior knowledge of their potential relevance to intestinal inflammation and immune dysregulation. For instance, analyses included *Clostridium difficile* and *Campylobacter* among bacterial infections, and *Candida albicans* among fungal infections. This approach allowed us to evaluate whether individual microorganisms, beyond the broader infection categories, conferred distinct risks of IBD (4,5).

**Covariates**

Adjusted covariates included age (continuous), sex (male or female), ethnicity (white, non-white, or unknown), body mass index (BMI, continuous), physical activity (regular or irregular), smoking status (never, former, or current smoker), and drinking status (never, former, or current drinker). Additional covariates included the INFLA score, a composite index reflecting chronic low-grade inflammation based on C-reactive protein (CRP), white blood cell count, platelet count, and the neutrophil-to-lymphocyte ratio, with higher values indicating greater inflammatory burden, as well as the Charlson Comorbidity Index (CCI), which quantifies baseline comorbidities. To handle missing data, continuous variables were imputed using the mean value, while categorical variables, all with <3% missingness, were imputed using the most frequent category.

**Genetic-related analysis**

To perform risk stratification, polygenic risk scores (PRS) were constructed to quantify genetic susceptibility to IBD, including Crohn’s disease (CD) and ulcerative colitis (UC). The detailed methodology for PRS construction has been described previously. Briefly, PRS for CD and UC were generated separately using 48 and 67 independent single nucleotide polymorphisms (SNPs), respectively. These SNPs were identified based on genome-wide significance (P < 5 × 10⁻⁸) and stringent linkage disequilibrium thresholds (r² < 0.001), derived from a large genome-wide association study (GWAS) of 86,640 individuals of European ancestry (6,7). Participants were categorized into high or low PRS groups according to the empirical distribution of scores. Multiplicative interaction between infection status and PRS category (high vs. low) was evaluated using Cox regression models stratified by PRS groups.

To prioritize immune-related genes implicated in IBD, we first extracted 320 genome-wide significant susceptibility loci from the largest available IBD GWAS meta-analysis (8). Each locus had been mapped to a single representative gene and was represented by a lead SNP. Variants within these loci were functionally annotated using integrated genomic resources, including NCBI ([https://www.ncbi.nlm.nih.gov/](https://www.ncbi.nlm.nih.gov/?utm_source=chatgpt.com)), GeneCards ([https://www.genecards.org/](https://www.genecards.org/?utm_source=chatgpt.com)), and the GWAS Catalog ([https://www.ebi.ac.uk/gwas/](https://www.ebi.ac.uk/gwas/?utm_source=chatgpt.com)). Based on these annotations, we prioritized 51 loci mapping to genes with established roles in host immunity or pathogen defense for detailed investigation.

For each locus, whole-genome sequencing (WGS) data from the UK Biobank were used to obtain accurate genotype information and to perform genotype-stratified analyses. Participants were grouped into three genotype categories (homozygous reference, heterozygous, homozygous alternate), and we classified the inheritance model of each SNP as dominant or recessive based on effect estimate patterns. Specifically, a dominant model was assigned when hazard ratios (HRs) were similar between heterozygotes and homozygous alternates, while a recessive model was inferred when HRs were similar between heterozygotes and homozygous references.

Interaction analysis identified 44 of the 51 prioritized loci as exhibiting significant interactions with infectious exposures. We assessed associations between genetic variants and inflammation-related phenotypes. Inflammatory markers included C-reactive protein (CRP), white blood cell count (WBC), platelet count (PLT), absolute counts of lymphocytes, neutrophils, eosinophils, basophils, and monocytes; red cell distribution width; the percentages of lymphocytes, monocytes, and neutrophils; and ratio-based indices including neutrophil-to-lymphocyte ratio (NLR), platelet-to-lymphocyte ratio (PLR), lymphocyte-to-monocyte ratio (LMR), systemic immune-inflammation index (SII), and inflammation score (INFLA). For each marker, extreme outliers (values < Q1−3×IQR or > Q3+3×IQR) were considered implausible and excluded; no imputation was performed. To examine different genetic models, each SNP genotype was recoded into binary groups for a recessive model and a dominant model. Group differences in continuous markers were tested using two-sided Wilcoxon rank-sum tests. Multiple testing was controlled using the Benjamini–Hochberg false discovery rate, applied separately within each genetic model (9).

We used these to develop an Infection IBD Score (IIS) by integrating genotype-specific weights across loci. Weights were assigned according to the direction and inheritance model of each variant’s interaction effect: For dominant variants, the reference homozygote was coded −1, and heterozygote/alternate homozygote were coded +1 when the alternate allele increased infection-associated IBD risk; the coding was reversed when the alternate allele reduced risk. For recessive variants, the alternate homozygote was coded +1 and the other genotypes −1 when risk was increased; the coding was reversed when risk was reduced.

Using the same framework, we further derived pathogen-specific IIS for bacterial, viral, and fungal infections. Parasitic infections were excluded due to insufficient case numbers. Participants were first classified into high and low IIS groups based on the median distribution, and multiplicative interaction P values were calculated to assess statistical significance. To evaluate potential dose–response relationships, IIS values were additionally categorized into quintiles.

To assess whether prioritized infection-interacting loci captured distinct immune-genetic vulnerability patterns, we performed unsupervised clustering based on the genotype matrix of these loci. Principal component analysis (PCA) was first used to reduce dimensionality and summarize the major axes of genetic variation. Principal components explaining at least 70% of the cumulative variance were retained and used for K-means clustering. The optimal number of clusters was evaluated using the elbow method based on within-cluster sum of squares (WSS). Across IBD, CD, and UC, WSS showed the largest reduction from K = 1 to K = 2, followed by smaller incremental decreases with additional clusters, supporting a two-cluster solution. The resulting cluster assignments were then used in stratified analyses of post-infection IBD risk. To evaluate shared and disease-specific genetic patterns, we further performed overlap analyses of cluster-enriched genes across IBD, CD, and UC.

To evaluate whether clustering based on the 44 previously identified infection-interacting SNPs could distinguish individuals with elevated post-infection IBD risk, we conducted the following analyses. Principal component analysis (PCA) was first applied to the genotype data of these SNPs to reduce dimensionality and capture major genetic variation patterns. Principal components explaining at least 70% of the cumulative variance were retained for clustering. The optimal number of clusters was determined using the elbow method based on the within-cluster sum of squares, which supported a two-cluster solution. Subsequently, k-means clustering was performed using the selected components, and the resulting cluster assignments were employed for stratified analyses of the participants. To further investigate the biological functions and pathways of the identified genes, Kyoto Encyclopedia of Genes and Genomes (KEGG) enrichment analysis was performed. This analysis enabled the identification of major functional categories and signaling pathways associated with the selected genes.

Supplementary Figure

## Figure S1. Associations of (A) detailed infection types and (B) infection locations with incident IBD, including CD and UC.

## Figure S2. Associations between immune-related SNPs and inflammation-related laboratory markers.

NLR, neutrophil-to-lymphocyte ratio; PLR, platelet-to-lymphocyte ratio; LMR, lymphocyte-to-monocyte ratio; SII, systemic immune-inflammation index; INFLA, low-grade inflammation score; Statistical significance was indicated by asterisks based on adjusted *P* values: *** for *P* < 0.001, ** for *P* < 0.01, * for *P* < 0.05, and no mark for *P* ≥ 0.05.

## Figure S3. Elbow plots for determining the optimal number of clusters in k-means clustering of immune-related genetic variants.

Elbow plots based on within-cluster sum of squares are shown for IBD-, CD-, and UC-associated immune-related genetic variants. The dashed vertical line indicates the selected two-cluster solution (K = 2), which was used for subsequent stratified and overlap analyses.

## Figure S4. Gene-profile similarity of cluster-enriched immune-related genes across IBD, CD, and UC.

(A) Pairwise Jaccard similarity of cluster-enriched immune-related genes across IBD, CD, and UC clusters. Jaccard index was calculated as the number of shared genes divided by the union of genes in each pairwise comparison. (B) Venn plots showing shared and disease-specific cluster-enriched genes within the Cluster 1 and Cluster 2 across IBD, CD, and UC. Shared genes across IBD, CD, and UC are listed below each Venn plot. (C) Venn plots showing shared and disease-specific cluster-enriched genes between CD and UC within the Cluster 1 and Cluster 2. Shared genes between CD and UC are listed below each Venn plot.

## Figure S5. Clustering of immune-related SNPs and stratified risk analysis for IBD, CD, and UC.

Stratified analyses of post-infection risk across clusters for overall infectious diseases and subtypes. Hazard ratios (HRs) with 95% confidence intervals (CIs) were estimated, and P values for interaction were calculated to assess heterogeneity between clusters.

## Figure S6. Quantile-based stratification of post-infection IBD, CD, and UC risk by reduced IIS after excluding low-evidence SNPs.

## Figure S7. Cumulative incidence of IBD, CD and UC according to infection type in competing risk models accounting for death as a competing event.

Cumulative incidence curves of (A) IBD, (B) CD, and (C) UC according to infection type (all infections, bacterial, viral, parasitic, and fungal) in competing risk models accounting for death as a competing event. The y-axis represents cumulative incidence (%), and the x-axis represents follow-up time (years). The tables below each panel show cumulative incidence estimates (%, 95% CI) at 1, 3, 6, 9, and 12 years.

## Figure S8. Age-specific associations between infection and risk of IBD, CD, and UC.

HRs were estimated in fully adjusted Cox proportional hazards models across predefined age categories (<45, 46–50, 51–55, 56–60, 61–65, ≥66 years).

Supplementary Tables

## Table S1. STROBE Statement—Checklist of items that should be included in reports of cohort studies.

|  | Item No | Recommendation | Page number |
| --- | --- | --- | --- |
| **Title and abstract** | 1 | (*a*) Indicate the study’s design with a commonly used term in the title or the abstract | 1 |
|  |  | (*b*) Provide in the abstract an informative and balanced summary of what was done and what was found | 3 |
| Introduction | | |  |
| Background/rationale | 2 | Explain the scientific background and rationale for the investigation being reported | 4 |
| Objectives | 3 | State specific objectives, including any prespecified hypotheses | 5 |
| Methods | | |  |
| Study design | 4 | Present key elements of study design early in the paper | 17 |
| Setting | 5 | Describe the setting, locations, and relevant dates, including periods of recruitment, exposure, follow-up, and data collection | 17 |
| Participants | 6 | (*a*) Give the eligibility criteria, and the sources and methods of selection of participants. Describe methods of follow-up | 17-18 |
|  |  | (*b*) For matched studies, give matching criteria and number of exposed and unexposed | NA |
| Variables | 7 | Clearly define all outcomes, exposures, predictors, potential confounders, and effect modifiers. Give diagnostic criteria, if applicable | 17-19, Tables S3, S33-35 |
| Data sources/ measurement | 8* | For each variable of interest, give sources of data and details of methods of assessment (measurement). Describe comparability of assessment methods if there is more than one group | 17-19 |
| Bias | 9 | Describe any efforts to address potential sources of bias | 20-21 |
| Study size | 10 | Explain how the study size was arrived at | 17 |
| Quantitative variables | 11 | Explain how quantitative variables were handled in the analyses. If applicable, describe which groupings were chosen and why | 18, Table S2 |
| Statistical methods | 12 | (*a*) Describe all statistical methods, including those used to control for confounding | 19-21 |
|  |  | (*b*) Describe any methods used to examine subgroups and interactions | 19-21 |
|  |  | (*c*) Explain how missing data were addressed | 18 |
|  |  | (*d*) If applicable, explain how loss to follow-up was addressed | NA |
|  |  | (*e*) Describe any sensitivity analyses | 20-21 |
| Results | | |  |
| Participants | 13* | (a) Report numbers of individuals at each stage of study—eg numbers potentially eligible, examined for eligibility, confirmed eligible, included in the study, completing follow-up, and analysed | 6, Figure 1 |
|  |  | (b) Give reasons for non-participation at each stage | Figure 1 |
|  |  | (c) Consider use of a flow diagram | Figure 1 |
| Descriptive data | 14* | (a) Give characteristics of study participants (eg demographic, clinical, social) and information on exposures and potential confounders | 6, Table 1 |
|  |  | (b) Indicate number of participants with missing data for each variable of interest | Table S2 |
|  |  | (c) Summarise follow-up time (eg, average and total amount) | 6 |
| Outcome data | 15* | Report numbers of outcome events or summary measures over time | 6 |
| Main results | 16 | (*a*) Give unadjusted estimates and, if applicable, confounder-adjusted estimates and their precision (eg, 95% confidence interval). Make clear which confounders were adjusted for and why they were included | 6-7, Figure 2 |
|  |  | (*b*) Report category boundaries when continuous variables were categorized | 10, Table S1, Table S14-15 |
|  |  | (*c*) If relevant, consider translating estimates of relative risk into absolute risk for a meaningful time period | Figure 5 |
| Other analyses | 17 | Report other analyses done—eg analyses of subgroups and interactions, and sensitivity analyses | 10-11 |
| Discussion | | |  |
| Key results | 18 | Summarise key results with reference to study objectives | 12 |
| Limitations | 19 | Discuss limitations of the study, taking into account sources of potential bias or imprecision. Discuss both direction and magnitude of any potential bias | 15-16 |
| Interpretation | 20 | Give a cautious overall interpretation of results considering objectives, limitations, multiplicity of analyses, results from similar studies, and other relevant evidence | 12-15 |
| Generalisability | 21 | Discuss the generalisability (external validity) of the study results | 12-15 |
| Other information | | |  |
| Funding | 22 | Give the source of funding and the role of the funders for the present study and, if applicable, for the original study on which the present article is based | 22 |

## Table S2. Hospital-treated infections: hierarchical classification of infection types and subtypes.

| **129,619 participants with hospital-treated infections** | | | |
| --- | --- | --- | --- |
| **119,598 Bacterial infections** | **16,060 Viral infections** | **830 Parasitic infections** | **7,732 Fungal infections** |
| **By severity** | **By type of viral infection** |  | **By type of Fungal infections** |
| 62,584 Invasive Bacterial infections | 12,840 Acute viral infections |  | 4,409 superficial Funagl infections |
| 89,062 Localized Bacterial infections | 2,256 Herpesvirus infections |  | 3,648 deep Funagl infections |
| 14,047 Bacterial infections with sepsis | 1,585 Other persistent viral infections |  |  |
| 117,276 Bacterial infections without sepsis |  |  |  |
| **By location** |  |  |  |
| 23,225 Extracellular Bacterial infections |  |  |  |
| 1,712 Intracellular Bacterial infections |  |  |  |
| **By Gram stain** |  |  |  |
| 10,601 Gram-positive infections |  |  |  |
| 17,503 Gram-negative infections |  |  |  |
| **Pathogen-level** |  |  | **Pathogen-level** |
| 1,346 Clostridium difficile |  |  | 6,461 Candidal |
| 834 Campylobacter |  |  | 334 Aspergillosis |
| 192 Salmonella |  |  |  |

**Table S3**. Sensitivity analyses of the association between hospital-treated infection and incident IBD, CD, and UC using lag periods of 1, 2, 3, and 10 years after infection.

| **Lag period after infection** | **IBD** | | | **CD** | | | **UC** | | |
| --- | --- | --- | --- | --- | --- | --- | --- | --- | --- |
|  | **Case/person-year** | **HR (95%CI)** | ***P*** | **Case/person-year** | **HR (95%CI)** | ***P*** | **Case/person-year** | **HR (95%CI)** | ***P*** |
| Follow up from year 1 onwards | 884/1,260,664 | 2.93 (2.65, 3.23) | <0.001 | 360/1,257,704 | 4.06 (3.43, 4.82) | <0.001 | 638/1,259,272 | 2.87 (2.56, 3.22) | <0.001 |
| Follow up from year 2 onwards | 740/1,260,447 | 2.76 (2.48, 3.07) | <0.001 | 292/1,257,570 | 3.79 (3.16, 4.55) | <0.001 | 534/1,259,093 | 2.67 (2.36, 3.02) | <0.001 |
| Follow up from year 3 onwards | 627/1,260,166 | 2.62 (2.34, 2.93) | <0.001 | 256/1,257,472 | 3.71 (3.06, 4.50) | <0.001 | 442/1,258,849 | 2.49 (2.19, 2.84) | <0.001 |
| Follow up from year 10 onwards | 127/1,257,201 | 1.68 (1.35, 2.10) | <0.001 | 47/1,256,257 | 1.94 (1.33, 2.84) | <0.001 | 93/1,256,784 | 1.71 (1.32, 2.22) | <0.001 |

CI, confidence interval; HR, hazard ratio; IBD inflammatory bowel disease; CD crohn's disease; UC ulcerative colitis.

Based on the fully-adjusted model, adjusted for age, sex, ethnic background, education level, TDI, BMI, smoking status, alcohol consumption, physical activity.

**Table S4.** Associations Between Infectious Burden and the Risk of IBD, CD, and UC.

| **Number of simultaneous infections** | **IBD** | | | **CD** | | | **UC** | | |
| --- | --- | --- | --- | --- | --- | --- | --- | --- | --- |
|  | **Case/person-year** | **HR (95%CI)** | ***P*** | **Case/person-year** | **HR (95%CI)** | ***P*** | **Case/person-year** | **HR (95%CI)** | ***P*** |
| Single infection diagnosis | 482/726,505 | 2.48 (2.22, 2.78) | <0.001 | 200/725,412 | 3.49 (2.89, 4.23) | <0.001 | 340/725,973 | 2.37 (2.07, 2.71) | <0.001 |
| Two different infections diagnoses | 268/269,839 | 3.72 (3.23, 4.29) | <0.001 | 124/269,164 | 5.91 (4.72, 7.39) | <0.001 | 187/269,463 | 3.52 (2.98, 4.17) | <0.001 |
| Multiple different infection (>=3) diagnoses | 162/118,739 | 4.93 (4.13, 5.87) | <0.001 | 71/118,319 | 7.25 (5.49, 9.59) | <0.001 | 126/118,580 | 5.23 (4.28, 6.39) | <0.001 |
| P-trend |  |  | <0.001 |  |  | <0.001 |  |  | <0.001 |

CI, confidence interval; HR, hazard ratio; IBD, inflammatory bowel disease; CD, Crohn's disease; UC, ulcerative colitis.

Based on the fully-adjusted model, adjusted for age, sex, ethnic background, education level, TDI, BMI, smoking status, alcohol consumption, physical activity.

**Table S5.** Sensitivity analyses of the associations between infection and incident IBD, CD, and UC after exclusion of participants with gastrointestinal infections.

| **Exposure** | **IBD** | | | **CD** | | | **UC** | | |
| --- | --- | --- | --- | --- | --- | --- | --- | --- | --- |
|  | **Case/person-year** | **HR (95%CI)** | ***P*** | **Case/person-year** | **HR (95%CI)** | ***P*** | **Case/person-year** | **HR (95%CI)** | ***P*** |
| **All infectious** | 878/2,604,615 | Ref |  | 252/2,600,084 | Ref |  | 656/2,603,004 | Ref |  |
|  | 540/1,053,155 | 1.65 (1.48, 1.84) | <0.001 | 204/1,051,472 | 2.10 (1.74, 2.54) | <0.001 | 402/1,052,420 | 1.66 (1.46, 1.89) | <0.001 |
| **Bacterial infections** | 910/2,691,289 | Ref |  | 264/2,686,628 | Ref |  | 682/2,689,635 | Ref |  |
|  | 508/966,481 | 1.67 (1.50, 1.87) | <0.001 | 192/964,928 | 2.11 (1.74, 2.56) | <0.001 | 376/965,789 | 1.67 (1.46, 1.90) | <0.001 |
| **By severity** |  |  |  |  |  |  |  |  |  |
| Invasive bacterial infections | 1107/3,139,158 | Ref |  | 330/3,133,877 | Ref |  | 832/3,137,244 | Ref |  |
|  | 311/518,613 | 1.77 (1.55, 2.01) | <0.001 | 126/517,679 | 2.34 (1.89, 2.89) | <0.001 | 226/518,180 | 1.72 (1.48, 2.00) | <0.001 |
| Localized bacterial infections | 1061/2,981,689 | Ref |  | 325/2,976,577 | Ref |  | 791/2,979,819 | Ref |  |
|  | 357/676,081 | 1.56 (1.38, 1.77) | <0.001 | 131/674,979 | 1.80 (1.46, 2.21) | <0.001 | 267/675,605 | 1.59 (1.38, 1.83) | <0.001 |
| Bacterial infections with sepsis | 1352/3,587,082 | Ref |  | 429/3,581,053 | Ref |  | 1015/3,584,823 | Ref |  |
|  | 66/70,688 | 2.27 (1.77, 2.91) | <0.001 | 27/70,503 | 2.88 (1.94, 4.27) | <0.001 | 43/70,601 | 1.97 (1.44, 2.68) | <0.001 |
| Bacterial infections without sepsis | 916/2,702,235 | Ref |  | 268/2,697,570 | Ref |  | 684/2,700,570 | Ref |  |
|  | 502/955,535 | 1.67 (1.49, 1.87) | <0.001 | 188/953,986 | 2.07 (1.71, 2.51) | <0.001 | 374/954,854 | 1.68 (1.48, 1.91) | <0.001 |
| **By location** |  |  |  |  |  |  |  |  |  |
| Extracellular bacterial infections | 1297/3,492,966 | Ref |  | 408/3,487,082 | Ref |  | 967/3,490,759 | Ref |  |
|  | 121/164,804 | 1.92 (1.59, 2.32) | <0.001 | 48/164,474 | 2.34 (1.73, 3.17) | <0.001 | 91/164,665 | 1.96 (1.57, 2.43) | <0.001 |
| Intracellular bacterial infections | 1414/3,650,103 | Ref |  | 454/3,643,899 | Ref |  | 1056/3,647,761 | Ref |  |
|  | 4/7,667 | 1.39 (0.52, 3.70) | 0.514 | 2/7,657 | 2.15 (0.54, 8.65) | 0.280 | 2/7,663 | 0.93 (0.23, 3.72) | 0.917 |
| **By Gram stain** |  |  |  |  |  |  |  |  |  |
| Gram-positive infections | 1375/3,576,992 | Ref |  | 443/3,570,936 | Ref |  | 1024/3,574,698 | Ref |  |
|  | 43/80,778 | 1.35 (1.00, 1.84) | 0.052 | 13/80,620 | 1.23 (0.71, 2.14) | 0.468 | 34/80,726 | 1.45 (1.03, 2.04) | 0.035 |
| Gram-negative infections | 1317/3,549,041 | Ref |  | 413/3,543,081 | Ref |  | 984/3,546,796 | Ref |  |
|  | 101/108,729 | 2.40 (1.95, 2.95) | <0.001 | 43/108,474 | 3.13 (2.28, 4.31) | <0.001 | 74/108,628 | 2.37 (1.87, 3.02) | <0.001 |
| **Viral infections** | 1345/3,527,880 | Ref |  | 429/3,521,977 | Ref |  | 1003/3,525,651 | Ref |  |
|  | 73/129,890 | 1.57 (1.24, 1.99) | <0.001 | 27/129,579 | 1.72 (1.16, 2.54) | 0.007 | 55/129,773 | 1.62 (1.23, 2.12) | 0.001 |
| **By type of viral infection** |  |  |  |  |  |  |  |  |  |
| Acute viral infections | 1366/3,563,528 | Ref |  | 438/3,557,538 | Ref |  | 1017/3,561,253 | Ref |  |
|  | 52/94,242 | 1.50 (1.14, 1.99) | 0.004 | 18/94,018 | 1.54 (0.96, 2.47) | 0.073 | 41/94,171 | 1.62 (1.18, 2.21) | 0.003 |
| Herpesvirus (persistent) infections | 1399/3,639,403 | Ref |  | 448/3,633,262 | Ref |  | 1044/3,637,101 | Ref |  |
|  | 19/18,367 | 2.85 (1.81, 4.48) | <0.001 | 8/18,293 | 3.59 (1.78, 7.22) | <0.001 | 14/18,323 | 2.85 (1.68, 4.84) | <0.001 |
| Other persistent viral infections | 1411/3,634,564 | Ref |  | 453/3,628,379 | Ref |  | 1054/3,632,221 | Ref |  |
|  | 7/23,207 | 0.88 (0.42, 1.85) | 0.731 | 3/23,177 | 1.04 (0.33, 3.25) | 0.947 | 4/23,203 | 0.70 (0.26, 1.87) | 0.478 |
| **Parasitic infections** | 1414/3,650,466 | Ref |  | 453/3,644,255 | Ref |  | 1057/3,648,124 | Ref |  |
|  | 4/7,304 | 1.47 (0.55, 3.94) | 0.439 | 3/7,301 | 3.43 (1.10, 10.72) | 0.034 | 1/7,300 | 0.49 (0.07, 3.51) | 0.481 |
| **Fungal infections** | 1380/3,608,831 | Ref |  | 437/3,602,723 | Ref |  | 1031/3,606,541 | Ref |  |
|  | 38/48,939 | 1.96 (1.42, 2.71) | <0.001 | 19/48,833 | 2.90 (1.82, 4.60) | <0.001 | 27/48,883 | 1.90 (1.29, 2.79) | 0.001 |
| **By type of Fungal infections** |  |  |  |  |  |  |  |  |  |
| Superficial fungal infections | 1398/3,631,891 | Ref |  | 446/3,625,724 | Ref |  | 1045/3,629,577 | Ref |  |
|  | 20/25,880 | 1.96 (1.26, 3.05) | 0.003 | 10/25,832 | 2.81 (1.50, 5.27) | 0.001 | 13/25,847 | 1.75 (1.01, 3.03) | 0.045 |
| Deep fungal infections | 1397/3,632,799 | Ref |  | 445/3,626,646 | Ref |  | 1043/3,630,484 | Ref |  |
|  | 21/24,971 | 2.08 (1.35, 3.20) | 0.001 | 11/24,910 | 3.26 (1.79, 5.95) | <0.001 | 15/24,940 | 2.00 (1.20, 3.34) | 0.008 |

CI, confidence interval; HR, hazard ratio; IBD, inflammatory bowel disease; CD, Crohn's disease; UC, ulcerative colitis.

Based on the fully-adjusted model, adjusted for age, sex, ethnic background, education level, TDI, BMI, smoking status, alcohol consumption, physical activity.

## Table S6. Prioritized immune-related SNPs derived from genome-wide significant IBD susceptibility loci and incorporated into the IIS.

| SNP | Gene | SNP_index | IIS_value | Effect_IBD |
| --- | --- | --- | --- | --- |
| rs2413430 | CSF2RB | chr22:36908468:A:T | -1 | -0.1413 |
| rs6658353 | FCGR2A | chr1:161499264:G:C | -1 | -0.1039 |
| rs1882348 | IL18R1 | chr2:102368211:T:A | -1 | -0.0991 |
| rs4795894 | CCL7 | chr17:34264425:C:T | -1 | -0.0886 |
| rs1886730 | TNFRSF14 | chr1:2557169:T:C | -1 | -0.0593 |
| rs415704 | CLEC10A | chr17:7074375:C:T | -1 | -0.0586 |
| rs1363232 | TIMD4 | chr5:156956411:A:G | -1 | -0.0585 |
| rs727088 | CD226 | chr18:69863203:G:A | -1 | -0.0377 |
| rs4664304 | PLA2R1 | chr2:159937497:A:G | -1 | -0.0213 |
| rs7758080 | TAB2 | chr6:149255943:A:G | -1 | 0.0469 |
| rs4973341 | CCL20 | chr2:227795646:C:T | -1 | 0.0764 |
| rs1558746 | IFNG | chr12:68111348:G:A | -1 | 0.135 |
| rs1000113 | IRGM | chr5:150860514:C:T | -1 | 0.1561 |
| rs5743289 | NOD2 | chr16:50722863:C:T | -1 | 0.198 |
| rs11581607 | IL23R | chr1:67242007:G:A | 1 | -0.6024 |
| rs9271511 | HLA-DQA1 | chr6:32621706:G:A | 1 | -0.1643 |
| rs12601611 | STAT3 | chr17:42345810:C:T | 1 | -0.1332 |
| rs2595392 | ITGAV | chr2:186665389:C:G | 1 | -0.1042 |
| rs12722489 | IL2RA | chr10:6060049:C:T | 1 | -0.0875 |
| rs34687326 | SLAMF8 | chr1:159830120:G:A | 1 | -0.0855 |
| rs10041920 | SERINC5 | chr5:80229545:A:G | 1 | -0.0529 |
| rs743228 | NFKBIA | chr14:35390868:C:T | 1 | -0.046 |
| rs3176905 | CXCR5 | chr11:118884921:T:C | 1 | -0.0459 |
| rs3194051 | IL7R | chr5:35876172:A:G | 1 | 0.0372 |
| rs2073486 | IRF6 | chr1:209802870:G:A | 1 | 0.054 |
| rs3757387 | IRF5 | chr7:128936032:T:C | 1 | 0.0578 |
| rs73120731 | ELMO1 | chr7:37417880:A:G | 1 | 0.0666 |
| rs2284553 | IFNGR2 | chr21:33404389:A:G | 1 | 0.0718 |
| rs1373904 | LACC1 | chr13:43901262:A:G | 1 | 0.0783 |
| rs4787458 | IL27 | chr16:28519966:A:G | 1 | 0.0876 |
| rs755374 | IL12B | chr5:159402286:C:T | 1 | 0.168 |

IIS_value denotes the assigned SNP coding in the IIS based on the genetic model and direction of association with IBD, where +1 represents the genotype coded as contributing positively to the IIS and −1 represents the opposite coding. Effect_IBD denotes the published effect estimate of each SNP on IBD susceptibility, with positive and negative values indicating risk-increasing and protective effects, respectively.

## Table S7. Prioritized immune-related SNPs derived from genome-wide significant CD susceptibility loci and incorporated into the IIS.

| SNP | Gene | SNP_index | IIS_value | Effect_CD |
| --- | --- | --- | --- | --- |
| rs2595392 | ITGAV | chr2:186665389:C:G | -1 | -0.1197 |
| rs1882348 | IL18R1 | chr2:102368211:T:A | -1 | -0.1103 |
| rs9480634 | ATG5 | chr6:106020611:C:T | -1 | -0.0978 |
| rs727088 | CD226 | chr18:69863203:G:A | -1 | -0.04 |
| rs4664304 | PLA2R1 | chr2:159937497:A:G | -1 | -0.0281 |
| rs6658353 | FCGR2A | chr1:161499264:G:C | -1 | -0.0254 |
| rs1886730 | TNFRSF14 | chr1:2557169:T:C | -1 | -0.0187 |
| rs11672983 | FCAR | chr19:54871595:G:A | -1 | 0.0177 |
| rs616597 | NFKBIZ | chr3:101850882:A:C | -1 | 0.0545 |
| rs1558746 | IFNG | chr12:68111348:G:A | -1 | 0.0798 |
| rs7758080 | TAB2 | chr6:149255943:A:G | -1 | 0.0819 |
| rs11581607 | IL23R | chr1:67242007:G:A | 1 | -0.7662 |
| rs2413430 | CSF2RB | chr22:36908468:A:T | 1 | -0.1975 |
| rs12601611 | STAT3 | chr17:42345810:C:T | 1 | -0.1505 |
| rs12722489 | IL2RA | chr10:6060049:C:T | 1 | -0.1105 |
| rs4795894 | CCL7 | chr17:34264425:C:T | 1 | -0.1032 |
| rs1883832 | CD40 | chr20:46118343:C:T | 1 | -0.1004 |
| rs743228 | NFKBIA | chr14:35390868:C:T | 1 | -0.0918 |
| rs7898978 | MAP3K8 | chr10:30410871:T:C | 1 | -0.0809 |
| rs10041920 | SERINC5 | chr5:80229545:A:G | 1 | -0.0376 |
| rs2472649 | CXCL5 | chr4:73991991:A:G | 1 | 0.0114 |
| rs2073486 | IRF6 | chr1:209802870:G:A | 1 | 0.0559 |
| rs10910476 | IRF2BP2 | chr1:234599210:C:T | 1 | 0.062 |
| rs395157 | OSMR | chr5:38867630:C:T | 1 | 0.0798 |
| rs4973341 | CCL20 | chr2:227795646:C:T | 1 | 0.0801 |
| rs2284553 | IFNGR2 | chr21:33404389:A:G | 1 | 0.1096 |
| rs73120731 | ELMO1 | chr7:37417880:A:G | 1 | 0.1341 |
| rs1373904 | LACC1 | chr13:43901262:A:G | 1 | 0.1617 |
| rs1000113 | IRGM | chr5:150860514:C:T | 1 | 0.1822 |

IIS_value denotes the assigned SNP coding in the IIS based on the genetic model and direction of association with CD, where +1 represents the genotype coded as contributing positively to the IIS and −1 represents the opposite coding. Effect_CD denotes the published effect estimate of each SNP on IBD susceptibility, with positive and negative values indicating risk-increasing and protective effects, respectively.

## Table S8. Prioritized immune-related SNPs derived from genome-wide significant UC susceptibility loci and incorporated into the IIS.

| SNP | Gene | SNP_index | IIS_value | Effect_UC |
| --- | --- | --- | --- | --- |
| rs6658353 | FCGR2A | chr1:161499264:G:C | -1 | -0.1589 |
| rs1886730 | TNFRSF14 | chr1:2557169:T:C | -1 | -0.0936 |
| rs1882348 | IL18R1 | chr2:102368211:T:A | -1 | -0.0914 |
| rs2413430 | CSF2RB | chr22:36908468:A:T | -1 | -0.0747 |
| rs4795894 | CCL7 | chr17:34264425:C:T | -1 | -0.0684 |
| rs1363232 | TIMD4 | chr5:156956411:A:G | -1 | -0.0492 |
| rs415704 | CLEC10A | chr17:7074375:C:T | -1 | -0.0483 |
| rs727088 | CD226 | chr18:69863203:G:A | -1 | -0.0318 |
| rs743228 | NFKBIA | chr14:35390868:C:T | -1 | -0.0215 |
| rs4664304 | PLA2R1 | chr2:159937497:A:G | -1 | -0.0084 |
| rs5743289 | NOD2 | chr16:50722863:C:T | -1 | -0.0054 |
| rs389848 | RIPK2 | chr8:89784739:T:G | -1 | 0.022 |
| rs2073486 | IRF6 | chr1:209802870:G:A | -1 | 0.0498 |
| rs616597 | NFKBIZ | chr3:101850882:A:C | -1 | 0.0624 |
| rs4973341 | CCL20 | chr2:227795646:C:T | -1 | 0.0717 |
| rs395157 | OSMR | chr5:38867630:C:T | -1 | 0.0739 |
| rs1000113 | IRGM | chr5:150860514:C:T | -1 | 0.144 |
| rs73534586 | TRAF3IP2 | chr6:111594960:G:A | -1 | 0.1942 |
| rs11581607 | IL23R | chr1:67242007:G:A | 1 | -0.452 |
| rs9271511 | HLA-DQA1 | chr6:32621706:G:A | 1 | -0.3463 |
| rs12601611 | STAT3 | chr17:42345810:C:T | 1 | -0.1158 |
| rs2595392 | ITGAV | chr2:186665389:C:G | 1 | -0.0904 |
| rs12722489 | IL2RA | chr10:6060049:C:T | 1 | -0.0743 |
| rs7898978 | MAP3K8 | chr10:30410871:T:C | 1 | -0.0705 |
| rs9480634 | ATG5 | chr6:106020611:C:T | 1 | -0.0679 |
| rs34687326 | SLAMF8 | chr1:159830120:G:A | 1 | -0.0643 |
| rs10041920 | SERINC5 | chr5:80229545:A:G | 1 | -0.0633 |
| rs4986790 | TLR4 | chr9:117713024:A:G | 1 | -0.0015 |
| rs7758080 | TAB2 | chr6:149255943:A:G | 1 | 0.0121 |
| rs1373904 | LACC1 | chr13:43901262:A:G | 1 | 0.0138 |
| rs2284553 | IFNGR2 | chr21:33404389:A:G | 1 | 0.043 |
| rs4787458 | IL27 | chr16:28519966:A:G | 1 | 0.0609 |
| rs2472649 | CXCL5 | chr4:73991991:A:G | 1 | 0.0633 |
| rs3757387 | IRF5 | chr7:128936032:T:C | 1 | 0.1038 |
| rs755374 | IL12B | chr5:159402286:C:T | 1 | 0.1479 |
| rs16940202 | IRF8 | chr16:85980635:T:C | 1 | 0.1605 |
| rs1558746 | IFNG | chr12:68111348:G:A | 1 | 0.1919 |

IIS_value denotes the assigned SNP coding in the IIS based on the genetic model and direction of association with UC where +1 represents the genotype coded as contributing positively to the IIS and −1 represents the opposite coding. Effect_UC denotes the published effect estimate of each SNP on IBD susceptibility, with positive and negative values indicating risk-increasing and protective effects, respectively.

**Table S9.** KEGG pathway enrichment analysis according to evidence level of immune-related genes**.**

| Gene set | Description | GeneRatio | BgRatio | RichFactor | FoldEnrichment | pvalue | p.adjust | geneID | Count |
| --- | --- | --- | --- | --- | --- | --- | --- | --- | --- |
| All identified infection-associated genes | Cytokine-cytokine receptor interaction | 17/40 | 298/9446 | 0.057 | 13.472 | <0.001 | <0.001 | CCL20/CSF2RB/IFNG/IL18R1/IL12B/CCL7/IL7R/TNFRSF14/CXCR5/IL27/IL2RA/CD40/CXCL5/OSMR/IL23R/IFNGR2/TNFSF15 | 17 |
| All identified infection-associated genes | JAK-STAT signaling pathway | 10/40 | 168/9446 | 0.060 | 14.057 | <0.001 | <0.001 | CSF2RB/IFNG/IL12B/STAT3/IL7R/IL27/IL2RA/OSMR/IL23R/IFNGR2 | 10 |
| All identified infection-associated genes | Th17 cell differentiation | 8/40 | 109/9446 | 0.073 | 17.332 | <0.001 | <0.001 | IFNG/HLA-DQA1/STAT3/IL27/IL2RA/NFKBIA/IL23R/IFNGR2 | 8 |
| All identified infection-associated genes | IL-17 signaling pathway | 7/40 | 95/9446 | 0.074 | 17.401 | <0.001 | <0.001 | TAB2/CCL20/IFNG/CCL7/CXCL5/TRAF3IP2/NFKBIA | 7 |
| All identified infection-associated genes | Viral protein interaction with cytokine and cytokine receptor | 7/40 | 100/9446 | 0.070 | 16.531 | <0.001 | <0.001 | CCL20/IL18R1/CCL7/TNFRSF14/CXCR5/IL2RA/CXCL5 | 7 |
| All identified infection-associated genes | Toll-like receptor signaling pathway | 7/40 | 109/9446 | 0.064 | 15.166 | <0.001 | <0.001 | TAB2/IL12B/IRF5/MAP3K8/CD40/TLR4/NFKBIA | 7 |
| All identified infection-associated genes | TNF signaling pathway | 7/40 | 119/9446 | 0.059 | 13.891 | <0.001 | <0.001 | TAB2/CCL20/IL18R1/MAP3K8/NOD2/CXCL5/NFKBIA | 7 |
| All identified infection-associated genes | Th1 and Th2 cell differentiation | 6/40 | 93/9446 | 0.065 | 15.235 | <0.001 | <0.001 | IFNG/IL12B/HLA-DQA1/IL2RA/NFKBIA/IFNGR2 | 6 |
| All identified infection-associated genes | NOD-like receptor signaling pathway | 7/40 | 189/9446 | 0.037 | 8.746 | <0.001 | <0.001 | TAB2/IRGM/NOD2/ATG5/RIPK2/TLR4/NFKBIA | 7 |
| All identified infection-associated genes | Chemokine signaling pathway | 7/40 | 193/9446 | 0.036 | 8.565 | <0.001 | <0.001 | CCL20/ELMO1/STAT3/CCL7/CXCR5/CXCL5/NFKBIA | 7 |
| All identified infection-associated genes | Phagosome | 6/40 | 159/9446 | 0.038 | 8.911 | <0.001 | <0.001 | HLA-DQA1/FCGR2A/PLA2R1/FCAR/TLR4/ITGAV | 6 |
| All identified infection-associated genes | Osteoclast differentiation | 5/40 | 143/9446 | 0.035 | 8.257 | <0.001 | 0.001 | TAB2/IFNG/FCGR2A/NFKBIA/IFNGR2 | 5 |
| All identified infection-associated genes | Type I diabetes mellitus | 3/40 | 44/9446 | 0.068 | 16.101 | 0.001 | 0.003 | IFNG/IL12B/HLA-DQA1 | 3 |
| All identified infection-associated genes | NF-kappa B signaling pathway | 4/40 | 105/9446 | 0.038 | 8.996 | 0.001 | 0.004 | TAB2/CD40/TLR4/NFKBIA | 4 |
| All identified infection-associated genes | HIF-1 signaling pathway | 4/40 | 110/9446 | 0.036 | 8.587 | 0.001 | 0.004 | IFNG/STAT3/TLR4/IFNGR2 | 4 |
| All identified infection-associated genes | Alcoholic liver disease | 4/40 | 144/9446 | 0.028 | 6.560 | 0.003 | 0.010 | TAB2/IL12B/TLR4/NFKBIA | 4 |
| All identified infection-associated genes | RIG-I-like receptor signaling pathway | 3/40 | 72/9446 | 0.042 | 9.840 | 0.003 | 0.011 | IL12B/ATG5/NFKBIA | 3 |
| All identified infection-associated genes | Necroptosis | 4/40 | 159/9446 | 0.025 | 5.941 | 0.004 | 0.014 | IFNG/STAT3/TLR4/IFNGR2 | 4 |
| All identified infection-associated genes | Cell adhesion molecules | 4/40 | 160/9446 | 0.025 | 5.904 | 0.005 | 0.014 | HLA-DQA1/CD226/CD40/ITGAV | 4 |
| All identified infection-associated genes | Hematopoietic cell lineage | 3/40 | 100/9446 | 0.030 | 7.085 | 0.009 | 0.024 | HLA-DQA1/IL7R/IL2RA | 3 |
| All identified infection-associated genes | T cell receptor signaling pathway | 3/40 | 122/9446 | 0.025 | 5.807 | 0.015 | 0.035 | IFNG/MAP3K8/NFKBIA | 3 |
| All identified infection-associated genes | PI3K-Akt signaling pathway | 5/40 | 362/9446 | 0.014 | 3.262 | 0.018 | 0.040 | IL7R/IL2RA/OSMR/TLR4/ITGAV | 5 |
| All identified infection-associated genes | Intestinal immune network for IgA production | 2/40 | 50/9446 | 0.040 | 9.446 | 0.019 | 0.042 | HLA-DQA1/CD40 | 2 |
| High- and moderate-evidence genes | Cytokine-cytokine receptor interaction | 15/38 | 298/9446 | 0.050 | 12.512 | 0.000 | 0.000 | IL2RA/IL27/CXCR5/TNFRSF14/IL7R/CCL7/IFNG/IL18R1/IL12B/CSF2RB/CCL20/CXCL5/CD40/OSMR/IFNGR2 | 15 |
| High- and moderate-evidence genes | JAK-STAT signaling pathway | 9/38 | 168/9446 | 0.054 | 13.317 | 0.000 | 0.000 | IL2RA/IL27/IL7R/IFNG/IL12B/CSF2RB/STAT3/OSMR/IFNGR2 | 9 |
| High- and moderate-evidence genes | IL-17 signaling pathway | 7/38 | 95/9446 | 0.074 | 18.316 | 0.000 | 0.000 | CCL7/IFNG/CCL20/TAB2/NFKBIA/CXCL5/TRAF3IP2 | 7 |
| High- and moderate-evidence genes | Viral protein interaction with cytokine and cytokine receptor | 7/38 | 100/9446 | 0.070 | 17.401 | 0.000 | 0.000 | IL2RA/CXCR5/TNFRSF14/CCL7/IL18R1/CCL20/CXCL5 | 7 |
| High- and moderate-evidence genes | Toll-like receptor signaling pathway | 7/38 | 109/9446 | 0.064 | 15.964 | 0.000 | 0.000 | IRF5/IL12B/TAB2/NFKBIA/CD40/MAP3K8/TLR4 | 7 |
| High- and moderate-evidence genes | Th17 cell differentiation | 7/38 | 109/9446 | 0.064 | 15.964 | 0.000 | 0.000 | IL2RA/IL27/IFNG/HLA-DQA1/STAT3/NFKBIA/IFNGR2 | 7 |
| High- and moderate-evidence genes | TNF signaling pathway | 7/38 | 119/9446 | 0.059 | 14.622 | 0.000 | 0.000 | NOD2/IL18R1/CCL20/TAB2/NFKBIA/CXCL5/MAP3K8 | 7 |
| High- and moderate-evidence genes | Th1 and Th2 cell differentiation | 6/38 | 93/9446 | 0.065 | 16.037 | 0.000 | 0.000 | IL2RA/IFNG/HLA-DQA1/IL12B/NFKBIA/IFNGR2 | 6 |
| High- and moderate-evidence genes | NOD-like receptor signaling pathway | 7/38 | 189/9446 | 0.037 | 9.207 | 0.000 | 0.000 | NOD2/IRGM/TAB2/NFKBIA/ATG5/RIPK2/TLR4 | 7 |
| High- and moderate-evidence genes | Chemokine signaling pathway | 7/38 | 193/9446 | 0.036 | 9.016 | 0.000 | 0.000 | CXCR5/CCL7/ELMO1/CCL20/STAT3/NFKBIA/CXCL5 | 7 |
| High- and moderate-evidence genes | Phagosome | 6/38 | 159/9446 | 0.038 | 9.380 | 0.000 | 0.000 | FCGR2A/PLA2R1/HLA-DQA1/ITGAV/FCAR/TLR4 | 6 |
| High- and moderate-evidence genes | Osteoclast differentiation | 5/38 | 143/9446 | 0.035 | 8.692 | 0.000 | 0.001 | FCGR2A/IFNG/TAB2/NFKBIA/IFNGR2 | 5 |
| High- and moderate-evidence genes | Type I diabetes mellitus | 3/38 | 44/9446 | 0.068 | 16.949 | 0.001 | 0.003 | IFNG/HLA-DQA1/IL12B | 3 |
| High- and moderate-evidence genes | NF-kappa B signaling pathway | 4/38 | 105/9446 | 0.038 | 9.470 | 0.001 | 0.003 | TAB2/NFKBIA/CD40/TLR4 | 4 |
| High- and moderate-evidence genes | HIF-1 signaling pathway | 4/38 | 110/9446 | 0.036 | 9.039 | 0.001 | 0.004 | IFNG/STAT3/IFNGR2/TLR4 | 4 |
| High- and moderate-evidence genes | Alcoholic liver disease | 4/38 | 144/9446 | 0.028 | 6.905 | 0.003 | 0.009 | IL12B/TAB2/NFKBIA/TLR4 | 4 |
| High- and moderate-evidence genes | RIG-I-like receptor signaling pathway | 3/38 | 72/9446 | 0.042 | 10.357 | 0.003 | 0.010 | IL12B/NFKBIA/ATG5 | 3 |
| High- and moderate-evidence genes | Necroptosis | 4/38 | 159/9446 | 0.025 | 6.254 | 0.004 | 0.011 | IFNG/STAT3/IFNGR2/TLR4 | 4 |
| High- and moderate-evidence genes | Cell adhesion molecules | 4/38 | 160/9446 | 0.025 | 6.214 | 0.004 | 0.011 | CD226/HLA-DQA1/CD40/ITGAV | 4 |
| High- and moderate-evidence genes | Hematopoietic cell lineage | 3/38 | 100/9446 | 0.030 | 7.457 | 0.007 | 0.021 | IL2RA/IL7R/HLA-DQA1 | 3 |
| High- and moderate-evidence genes | T cell receptor signaling pathway | 3/38 | 122/9446 | 0.025 | 6.113 | 0.013 | 0.030 | IFNG/NFKBIA/MAP3K8 | 3 |
| High- and moderate-evidence genes | PI3K-Akt signaling pathway | 5/38 | 362/9446 | 0.014 | 3.433 | 0.014 | 0.032 | IL2RA/IL7R/ITGAV/OSMR/TLR4 | 5 |
| High- and moderate-evidence genes | Intestinal immune network for IgA production | 2/38 | 50/9446 | 0.040 | 9.943 | 0.017 | 0.038 | HLA-DQA1/CD40 | 2 |
| High-evidence genes | Toll-like receptor signaling pathway | 5/15 | 109/9446 | 0.046 | 28.887 | 0.000 | 0.000 | IRF5/IL12B/TAB2/MAP3K8/NFKBIA | 5 |
| High-evidence genes | Th17 cell differentiation | 5/15 | 109/9446 | 0.046 | 28.887 | 0.000 | 0.000 | IFNG/HLA-DQA1/STAT3/NFKBIA/IL27 | 5 |
| High-evidence genes | TNF signaling pathway | 6/15 | 119/9446 | 0.042 | 26.459 | 0.000 | 0.000 | IL18R1/CCL20/TAB2/MAP3K8/NFKBIA | 5 |
| High-evidence genes | Cytokine-cytokine receptor interaction | 46188 | 298/9446 | 0.020 | 12.679 | 0.000 | 0.000 | IFNG/IL18R1/IL12B/CSF2RB/CCL20/IL27 | 6 |
| High-evidence genes | JAK-STAT signaling pathway | 5/15 | 168/9446 | 0.030 | 18.742 | 0.000 | 0.000 | IFNG/IL12B/CSF2RB/STAT3/IL27 | 5 |
| High-evidence genes | Th1 and Th2 cell differentiation | 4/15 | 93/9446 | 0.043 | 27.085 | 0.000 | 0.000 | IFNG/HLA-DQA1/IL12B/NFKBIA | 4 |
| High-evidence genes | IL-17 signaling pathway | 4/15 | 95/9446 | 0.042 | 26.515 | 0.000 | 0.000 | IFNG/CCL20/TAB2/NFKBIA | 4 |
| High-evidence genes | Type I diabetes mellitus | 3/15 | 44/9446 | 0.068 | 42.936 | 0.000 | 0.000 | IFNG/HLA-DQA1/IL12B | 3 |
| High-evidence genes | Chemokine signaling pathway | 4/15 | 193/9446 | 0.021 | 13.051 | 0.000 | 0.001 | ELMO1/CCL20/STAT3/NFKBIA | 4 |
| High-evidence genes | T cell receptor signaling pathway | 3/15 | 122/9446 | 0.025 | 15.485 | 0.001 | 0.003 | IFNG/MAP3K8/NFKBIA | 3 |
| High-evidence genes | Osteoclast differentiation | 3/15 | 143/9446 | 0.021 | 13.211 | 0.001 | 0.005 | IFNG/TAB2/NFKBIA | 3 |
| High-evidence genes | Alcoholic liver disease | 3/15 | 144/9446 | 0.021 | 13.119 | 0.001 | 0.005 | IL12B/TAB2/NFKBIA | 3 |
| High-evidence genes | NOD-like receptor signaling pathway | 3/15 | 189/9446 | 0.016 | 9.996 | 0.003 | 0.009 | IRGM/TAB2/NFKBIA | 3 |
| High-evidence genes | Adipocytokine signaling pathway | 2/15 | 70/9446 | 0.029 | 17.992 | 0.005 | 0.015 | STAT3/NFKBIA | 2 |
| High-evidence genes | RIG-I-like receptor signaling pathway | 2/15 | 72/9446 | 0.028 | 17.493 | 0.006 | 0.015 | IL12B/NFKBIA | 2 |
| High-evidence genes | Antigen processing and presentation | 2/15 | 82/9446 | 0.024 | 15.359 | 0.007 | 0.018 | IFNG/HLA-DQA1 | 2 |
| High-evidence genes | Viral protein interaction with cytokine and cytokine receptor | 2/15 | 100/9446 | 0.020 | 12.595 | 0.011 | 0.026 | IL18R1/CCL20 | 2 |
| High-evidence genes | NF-kappa B signaling pathway | 2/15 | 105/9446 | 0.019 | 11.995 | 0.012 | 0.026 | TAB2/NFKBIA | 2 |
| High-evidence genes | C-type lectin receptor signaling pathway | 2/15 | 105/9446 | 0.019 | 11.995 | 0.012 | 0.026 | IL12B/NFKBIA | 2 |
| High-evidence genes | Insulin resistance | 2/15 | 109/9446 | 0.018 | 11.555 | 0.013 | 0.027 | STAT3/NFKBIA | 2 |
| High-evidence genes | HIF-1 signaling pathway | 2/15 | 110/9446 | 0.018 | 11.450 | 0.013 | 0.027 | IFNG/STAT3 | 2 |
| High-evidence genes | Apoptosis | 2/15 | 137/9446 | 0.015 | 9.193 | 0.019 | 0.040 | CSF2RB/NFKBIA | 2 |

**Table S10.** KEGG pathway enrichment analysis of prioritized immune-related genes implicated in infection-associated IBD, CD, and UC susceptibility**.**

| Disease | Description | GeneRatio | BgRatio | RichFactor | FoldEnrichment | pvalue | p.adjust | geneID | Count |
| --- | --- | --- | --- | --- | --- | --- | --- | --- | --- |
| IBD | Cytokine-cytokine receptor interaction | 13/28 | 298/9446 | 0.044 | 14.717 | <0.001 | <0.001 | IFNGR2/IL23R/IL2RA/IL27/CXCR5/TNFRSF14/IL7R/CCL7/IFNG/IL18R1/IL12B/CSF2RB/CCL20 | 13 |
| IBD | Th17 cell differentiation | 8/28 | 109/9446 | 0.073 | 24.760 | <0.001 | <0.001 | IFNGR2/IL23R/NFKBIA/IL2RA/IL27/IFNG/HLA-DQA1/STAT3 | 8 |
| IBD | JAK-STAT signaling pathway | 9/28 | 168/9446 | 0.054 | 18.073 | <0.001 | <0.001 | IFNGR2/IL23R/IL2RA/IL27/IL7R/IFNG/IL12B/CSF2RB/STAT3 | 9 |
| IBD | Th1 and Th2 cell differentiation | 6/28 | 93/9446 | 0.065 | 21.765 | <0.001 | <0.001 | IFNGR2/NFKBIA/IL2RA/IFNG/HLA-DQA1/IL12B | 6 |
| IBD | Viral protein interaction with cytokine and cytokine receptor | 6/28 | 100/9446 | 0.060 | 20.241 | <0.001 | <0.001 | IL2RA/CXCR5/TNFRSF14/CCL7/IL18R1/CCL20 | 6 |
| IBD | IL-17 signaling pathway | 5/28 | 95/9446 | 0.053 | 17.756 | <0.001 | <0.001 | NFKBIA/CCL7/IFNG/CCL20/TAB2 | 5 |
| IBD | Chemokine signaling pathway | 6/28 | 193/9446 | 0.031 | 10.488 | <0.001 | <0.001 | NFKBIA/CXCR5/CCL7/ELMO1/CCL20/STAT3 | 6 |
| IBD | TNF signaling pathway | 5/28 | 119/9446 | 0.042 | 14.175 | <0.001 | <0.001 | NFKBIA/NOD2/IL18R1/CCL20/TAB2 | 5 |
| IBD | Osteoclast differentiation | 5/28 | 143/9446 | 0.035 | 11.796 | <0.001 | <0.001 | IFNGR2/NFKBIA/FCGR2A/IFNG/TAB2 | 5 |
| IBD | Toll-like receptor signaling pathway | 4/28 | 109/9446 | 0.037 | 12.380 | <0.001 | 0.002 | NFKBIA/IRF5/IL12B/TAB2 | 4 |
| IBD | Type I diabetes mellitus | 3/28 | 44/9446 | 0.068 | 23.002 | <0.001 | 0.002 | IFNG/HLA-DQA1/IL12B | 3 |
| IBD | Phagosome | 4/28 | 159/9446 | 0.025 | 8.487 | 0.001 | 0.005 | ITGAV/FCGR2A/PLA2R1/HLA-DQA1 | 4 |
| IBD | NOD-like receptor signaling pathway | 4/28 | 189/9446 | 0.021 | 7.140 | 0.002 | 0.010 | NFKBIA/NOD2/IRGM/TAB2 | 4 |
| IBD | Hematopoietic cell lineage | 3/28 | 100/9446 | 0.030 | 10.121 | 0.003 | 0.013 | IL2RA/IL7R/HLA-DQA1 | 3 |
| IBD | HIF-1 signaling pathway | 3/28 | 110/9446 | 0.027 | 9.201 | 0.004 | 0.015 | IFNGR2/IFNG/STAT3 | 3 |
| IBD | Alcoholic liver disease | 3/28 | 144/9446 | 0.021 | 7.028 | 0.009 | 0.029 | NFKBIA/IL12B/TAB2 | 3 |
| IBD | Efferocytosis | 3/28 | 157/9446 | 0.019 | 6.446 | 0.011 | 0.034 | ITGAV/TIMD4/ELMO1 | 3 |
| IBD | Necroptosis | 3/28 | 159/9446 | 0.019 | 6.365 | 0.011 | 0.034 | IFNGR2/IFNG/STAT3 | 3 |
| IBD | Cell adhesion molecules | 3/28 | 160/9446 | 0.019 | 6.325 | 0.011 | 0.034 | ITGAV/CD226/HLA-DQA1 | 3 |
| IBD | Adipocytokine signaling pathway | 2/28 | 70/9446 | 0.029 | 9.639 | 0.018 | 0.050 | NFKBIA/STAT3 | 2 |
| CD | Cytokine-cytokine receptor interaction | 12/27 | 298/9446 | 0.040 | 14.088 | 0.000 | 0.000 | TNFRSF14/IL23R/IFNGR2/OSMR/CSF2RB/CXCL5/CD40/CCL7/CCL20/IL2RA/IFNG/IL18R1 | 12 |
| CD | IL-17 signaling pathway | 6/27 | 95/9446 | 0.063 | 22.096 | 0.000 | 0.000 | NFKBIA/CXCL5/CCL7/CCL20/IFNG/TAB2 | 6 |
| CD | Viral protein interaction with cytokine and cytokine receptor | 6/27 | 100/9446 | 0.060 | 20.991 | 0.000 | 0.000 | TNFRSF14/CXCL5/CCL7/CCL20/IL2RA/IL18R1 | 6 |
| CD | JAK-STAT signaling pathway | 7/27 | 168/9446 | 0.042 | 14.577 | 0.000 | 0.000 | IL23R/IFNGR2/OSMR/STAT3/CSF2RB/IL2RA/IFNG | 7 |
| CD | Th17 cell differentiation | 6/27 | 109/9446 | 0.055 | 19.258 | 0.000 | 0.000 | IL23R/IFNGR2/NFKBIA/STAT3/IL2RA/IFNG | 6 |
| CD | TNF signaling pathway | 6/27 | 119/9446 | 0.050 | 17.640 | 0.000 | 0.000 | NFKBIA/CXCL5/CCL20/IL18R1/TAB2/MAP3K8 | 6 |
| CD | Chemokine signaling pathway | 6/27 | 193/9446 | 0.031 | 10.876 | 0.000 | 0.000 | NFKBIA/STAT3/CXCL5/CCL7/CCL20/ELMO1 | 6 |
| CD | Osteoclast differentiation | 5/27 | 143/9446 | 0.035 | 12.233 | 0.000 | 0.000 | IFNGR2/NFKBIA/FCGR2A/IFNG/TAB2 | 5 |
| CD | Th1 and Th2 cell differentiation | 4/27 | 93/9446 | 0.043 | 15.047 | 0.000 | 0.001 | IFNGR2/NFKBIA/IL2RA/IFNG | 4 |
| CD | Toll-like receptor signaling pathway | 4/27 | 109/9446 | 0.037 | 12.839 | 0.000 | 0.002 | NFKBIA/CD40/TAB2/MAP3K8 | 4 |
| CD | Phagosome | 4/27 | 159/9446 | 0.025 | 8.801 | 0.001 | 0.007 | PLA2R1/FCGR2A/ITGAV/FCAR | 4 |
| CD | NOD-like receptor signaling pathway | 4/27 | 189/9446 | 0.021 | 7.404 | 0.002 | 0.011 | NFKBIA/IRGM/ATG5/TAB2 | 4 |
| CD | NF-kappa B signaling pathway | 3/27 | 105/9446 | 0.029 | 9.996 | 0.003 | 0.015 | NFKBIA/CD40/TAB2 | 3 |
| CD | HIF-1 signaling pathway | 3/27 | 110/9446 | 0.027 | 9.541 | 0.004 | 0.017 | IFNGR2/STAT3/IFNG | 3 |
| CD | T cell receptor signaling pathway | 3/27 | 122/9446 | 0.025 | 8.603 | 0.005 | 0.021 | NFKBIA/IFNG/MAP3K8 | 3 |
| CD | Necroptosis | 3/27 | 159/9446 | 0.019 | 6.601 | 0.010 | 0.035 | IFNGR2/STAT3/IFNG | 3 |
| CD | Cell adhesion molecules | 3/27 | 160/9446 | 0.019 | 6.560 | 0.010 | 0.035 | CD226/CD40/ITGAV | 3 |
| UC | Cytokine-cytokine receptor interaction | 13/34 | 298/9446 | 0.044 | 12.120 | 0.000 | 0.000 | IL23R/CXCL5/TNFRSF14/OSMR/IL2RA/IL18R1/IFNGR2/IFNG/CCL7/IL27/IL12B/CSF2RB/CCL20 | 13 |
| UC | Th17 cell differentiation | 8/34 | 109/9446 | 0.073 | 20.391 | 0.000 | 0.000 | IL23R/IL2RA/IFNGR2/IFNG/HLA-DQA1/NFKBIA/IL27/STAT3 | 8 |
| UC | JAK-STAT signaling pathway | 9/34 | 168/9446 | 0.054 | 14.883 | 0.000 | 0.000 | IL23R/OSMR/IL2RA/IFNGR2/IFNG/IL27/STAT3/IL12B/CSF2RB | 9 |
| UC | IL-17 signaling pathway | 7/34 | 95/9446 | 0.074 | 20.471 | 0.000 | 0.000 | CXCL5/TAB2/IFNG/CCL7/TRAF3IP2/NFKBIA/CCL20 | 7 |
| UC | TNF signaling pathway | 7/34 | 119/9446 | 0.059 | 16.343 | 0.000 | 0.000 | CXCL5/TAB2/NOD2/MAP3K8/IL18R1/NFKBIA/CCL20 | 7 |
| UC | Th1 and Th2 cell differentiation | 6/34 | 93/9446 | 0.065 | 17.924 | 0.000 | 0.000 | IL2RA/IFNGR2/IFNG/HLA-DQA1/NFKBIA/IL12B | 6 |
| UC | Viral protein interaction with cytokine and cytokine receptor | 6/34 | 100/9446 | 0.060 | 16.669 | 0.000 | 0.000 | CXCL5/TNFRSF14/IL2RA/IL18R1/CCL7/CCL20 | 6 |
| UC | Toll-like receptor signaling pathway | 6/34 | 109/9446 | 0.055 | 15.293 | 0.000 | 0.000 | TAB2/MAP3K8/IRF5/TLR4/NFKBIA/IL12B | 6 |
| UC | NOD-like receptor signaling pathway | 7/34 | 189/9446 | 0.037 | 10.290 | 0.000 | 0.000 | TAB2/RIPK2/NOD2/ATG5/TLR4/NFKBIA/IRGM | 7 |
| UC | Osteoclast differentiation | 5/34 | 143/9446 | 0.035 | 9.714 | 0.000 | 0.001 | TAB2/IFNGR2/IFNG/FCGR2A/NFKBIA | 5 |
| UC | Phagosome | 5/34 | 159/9446 | 0.031 | 8.737 | 0.000 | 0.001 | HLA-DQA1/FCGR2A/TLR4/ITGAV/PLA2R1 | 5 |
| UC | Type I diabetes mellitus | 3/34 | 44/9446 | 0.068 | 18.943 | 0.001 | 0.002 | IFNG/HLA-DQA1/IL12B | 3 |
| UC | Chemokine signaling pathway | 5/34 | 193/9446 | 0.026 | 7.198 | 0.001 | 0.003 | CXCL5/CCL7/NFKBIA/STAT3/CCL20 | 5 |
| UC | HIF-1 signaling pathway | 4/34 | 110/9446 | 0.036 | 10.103 | 0.001 | 0.003 | IFNGR2/IFNG/TLR4/STAT3 | 4 |
| UC | Alcoholic liver disease | 4/34 | 144/9446 | 0.028 | 7.717 | 0.002 | 0.006 | TAB2/TLR4/NFKBIA/IL12B | 4 |
| UC | RIG-I-like receptor signaling pathway | 3/34 | 72/9446 | 0.042 | 11.576 | 0.002 | 0.008 | ATG5/NFKBIA/IL12B | 3 |
| UC | Necroptosis | 4/34 | 159/9446 | 0.025 | 6.989 | 0.002 | 0.008 | IFNGR2/IFNG/TLR4/STAT3 | 4 |
| UC | NF-kappa B signaling pathway | 3/34 | 105/9446 | 0.029 | 7.938 | 0.006 | 0.018 | TAB2/TLR4/NFKBIA | 3 |
| UC | T cell receptor signaling pathway | 3/34 | 122/9446 | 0.025 | 6.832 | 0.009 | 0.026 | MAP3K8/IFNG/NFKBIA | 3 |
| UC | Cell adhesion molecules | 3/34 | 160/9446 | 0.019 | 5.209 | 0.019 | 0.048 | HLA-DQA1/CD226/ITGAV | 3 |

## Table S11. Prioritized immune-related SNPs from genome-wide significant IBD loci for infection-related modulation.

| SNP | Gene | Chr | GRCh38 position | Ref | Alt | Frequency of ref allele (1000 Genomes_30x-europe) |
| --- | --- | --- | --- | --- | --- | --- |
| rs1886730 | TNFRSF14 | chr1 | 2557169 | T | C | 0.5182 |
| rs140466198 | PTAFR | chr1 | 28131939 | C | T | 1.0000 |
| rs11581607 | IL23R | chr1 | 67242007 | G | A | 0.9400 |
| rs34687326 | SLAMF8 | chr1 | 159830120 | G | A | 0.8934 |
| rs6658353 | FCGR2A | chr1 | 161499264 | G | C | 0.4818 |
| rs3024495 | IL10 | chr1 | 206769068? | C | T | 0.8365 |
| rs2073486 | IRF6 | chr1 | 209802870 | G | A | 0.6453 |
| rs10910476 | IRF2BP2 | chr1 | 234599210 | C | T | 0.4566 |
| rs1882348 | IL18R1 | chr2 | 102368211 | T | A | 0.6177 |
| rs4664304 | PLA2R1 | chr2 | 159937497 | A | G | 0.4542 |
| rs2124440 | ITGA4 | chr2 | 181463487 | G | A | 0.4712 |
| rs2595392 | ITGAV | chr2 | 186665389 | C | G | 0.8997 |
| rs4973341 | CCL20 | chr2 | 227795646 | C | T | 0.3444 |
| rs616597 | NFKBIZ | chr3 | 101850882 | A | C | 0.2275 |
| rs2472649 | CXCL5 | chr4 | 73991991 | A | G | 0.1872 |
| rs3194051 | IL7R | chr5 | 35876172 | A | G | 0.7148 |
| rs395157 | OSMR | chr5 | 38867630 | C | T | 0.4866 |
| rs10041920 | SERINC5 | chr5 | 80229545 | A | G | 0.5474 |
| rs1000113 | IRGM | chr5 | 150860514 | C | T | 0.9005 |
| rs1363232 | TIMD4 | chr5 | 156956411 | A | G | 0.3294 |
| rs755374 | IL12B | chr5 | 159402286 | C | T | 0.6706 |
| rs9271511 | HLA-DQA1 | chr6 | 32621706 | G | A | 0.6801 |
| rs9480634 | ATG5 | chr6 | 106020611 | C | T | 0.4368 |
| rs73534586 | TRAF3IP2 | chr6 | 111594960 | G | A | 0.9360 |
| rs7758080 | TAB2 | chr6 | 149255943 | A | G | 0.7378 |
| rs73120731 | ELMO1 | chr7 | 37417880 | A | G | 0.8728 |
| rs3757387 | IRF5 | chr7 | 128936032 | T | C | 0.5521 |
| rs389848 | RIPK2 | chr8 | 89784739 | T | G | 0.4289 |
| rs56211063 | TNFSF15 | chr9 | 114823617 | G | C | 0.9889 |
| rs4986790 | TLR4 | chr9 | 117713024 | A | G | 0.9487 |
| rs12722489 | IL2RA | chr10 | 6060049 | C | T | 0.8570 |
| rs4748198 | PRKCQ | chr10 | 6607973 | C | T | 0.7954 |
| rs7898978 | MAP3K8 | chr10 | 30410871 | T | C | 0.7109 |
| rs3176905 | CXCR5 | chr11 | 118884921 | T | C | 0.8357 |
| rs4020660 | LTBR | chr12 | 6402491 | G | A | 0.5545 |
| rs1558746 | IFNG | chr12 | 68111348 | G | A | 0.6256 |
| rs1373904 | LACC1 | chr13 | 43901262 | A | G | 0.7796 |
| rs743228 | NFKBIA | chr14 | 35390868 | C | T | 0.5363 |
| rs4787458 | IL27 | chr16 | 28519966 | A | G | 0.6738 |
| rs5743289 | NOD2 | chr16 | 50722863 | C | T | 0.8239 |
| rs16940202 | IRF8 | chr16 | 85980635 | T | C | 0.8191 |
| rs415704 | CLEC10A | chr17 | 7074375 | C | T | 0.6635 |
| rs4795894 | CCL7 | chr17 | 34264425 | C | T | 0.6864 |
| rs12601611 | STAT3 | chr17 | 42345810 | C | T | 0.7212 |
| rs547268 | PTPN2 | chr18 | 12823057 | G | T | 0.1351 |
| rs727088 | CD226 | chr18 | 69863203 | G | A | 0.4637 |
| rs12720356 | TYK2 | chr19 | 10359299 | A | C | 0.9036 |
| rs11672983 | FCAR | chr19 | 54871595 | G | A | 0.5987 |
| rs1883832 | CD40 | chr20 | 46118343 | C | T | 0.2654 |
| rs2284553 | IFNGR2 | chr21 | 33404389 | A | G | 0.3902 |
| rs2413430 | CSF2RB | chr22 | 36908468 | A | T | 0.4352 |

**Table S12**. Proportion of Genetic Risk in Two Clusters of immune-related Genes in IBD.

| Gene | cluster1 | cluster2 | Difference |
| --- | --- | --- | --- |
| TNFRSF14 | 0.19 | 0.364 | -0.174 |
| IFNG | 0.338 | 0.444 | -0.106 |
| SERINC5 | 0.649 | 0.731 | -0.082 |
| IL18R1 | 0.341 | 0.408 | -0.067 |
| LACC1 | 0.358 | 0.409 | -0.051 |
| STAT3 | 0.07 | 0.089 | -0.019 |
| NOD2 | 0.966 | 0.976 | -0.01 |
| ELMO1 | 0.242 | 0.248 | -0.006 |
| IL7R | 0.066 | 0.07 | -0.004 |
| CXCR5 | 0.029 | 0.024 | 0.005 |
| IL23R | 0.128 | 0.119 | 0.009 |
| TIMD4 | 0.443 | 0.431 | 0.012 |
| ITGAV | 0.209 | 0.195 | 0.014 |
| IRF6 | 0.622 | 0.603 | 0.019 |
| CCL20 | 0.449 | 0.422 | 0.027 |
| IL12B | 0.567 | 0.534 | 0.033 |
| SLAMF8 | 0.235 | 0.2 | 0.035 |
| IL2RA | 0.306 | 0.271 | 0.035 |
| NFKBIA | 0.29 | 0.253 | 0.037 |
| IRGM | 0.865 | 0.828 | 0.037 |
| HLA-DQA1 | 0.142 | 0.101 | 0.041 |
| FCGR2A | 0.797 | 0.753 | 0.044 |
| CCL7 | 0.542 | 0.488 | 0.054 |
| TAB2 | 0.559 | 0.493 | 0.066 |
| IFNGR2 | 0.198 | 0.122 | 0.076 |
| CLEC10A | 0.913 | 0.829 | 0.084 |
| CD226 | 0.808 | 0.72 | 0.088 |
| IRF5 | 0.734 | 0.639 | 0.095 |
| CSF2RB | 0.849 | 0.72 | 0.129 |
| IL27 | 0.683 | 0.493 | 0.19 |
| PLA2R1 | 0.527 | 0.014 | 0.513 |

**Table S13.** Proportion of Genetic Risk in Two Clusters of immune-related Genes in CD.

| Gene | cluster1 | cluster2 | Difference |
| --- | --- | --- | --- |
| TNFRSF14 | 0.196 | 0.358 | -0.162 |
| CXCL5 | 0.277 | 0.410 | -0.133 |
| CSF2RB | 0.645 | 0.772 | -0.127 |
| IFNG | 0.340 | 0.444 | -0.104 |
| ATG5 | 0.778 | 0.867 | -0.089 |
| SERINC5 | 0.650 | 0.731 | -0.081 |
| CCL7 | 0.456 | 0.515 | -0.059 |
| LACC1 | 0.358 | 0.409 | -0.051 |
| FCAR | 0.365 | 0.405 | -0.040 |
| IL18R1 | 0.830 | 0.868 | -0.038 |
| NFKBIA | 0.710 | 0.747 | -0.037 |
| CCL20 | 0.549 | 0.581 | -0.032 |
| STAT3 | 0.070 | 0.090 | -0.020 |
| MAP3K8 | 0.086 | 0.102 | -0.016 |
| IRGM | 0.005 | 0.011 | -0.006 |
| NFKBIZ | 0.953 | 0.959 | -0.006 |
| ELMO1 | 0.242 | 0.248 | -0.006 |
| ITGAV | 0.988 | 0.989 | -0.001 |
| IRF6 | 0.145 | 0.142 | 0.003 |
| OSMR | 0.243 | 0.236 | 0.007 |
| IL23R | 0.129 | 0.118 | 0.011 |
| IL2RA | 0.307 | 0.270 | 0.037 |
| CD40 | 0.447 | 0.404 | 0.043 |
| FCGR2A | 0.797 | 0.752 | 0.045 |
| TAB2 | 0.559 | 0.492 | 0.067 |
| IFNGR2 | 0.195 | 0.124 | 0.071 |
| CD226 | 0.307 | 0.229 | 0.078 |
| IRF2BP2 | 0.865 | 0.697 | 0.168 |
| PLA2R1 | 0.522 | 0.014 | 0.508 |

**Table S14.** Proportion of Genetic Risk in Two Clusters of immune-related Genes in UC.

| Gene | cluster1 | cluster2 | Difference |
| --- | --- | --- | --- |
| TNFRSF14 | 0.198 | 0.359 | -0.161 |
| SERINC5 | 0.653 | 0.728 | -0.075 |
| IL18R1 | 0.341 | 0.410 | -0.069 |
| RIPK2 | 0.287 | 0.350 | -0.063 |
| CXCL5 | 0.022 | 0.079 | -0.057 |
| LACC1 | 0.358 | 0.410 | -0.052 |
| TAB2 | 0.064 | 0.091 | -0.027 |
| IRF6 | 0.376 | 0.400 | -0.024 |
| STAT3 | 0.070 | 0.090 | -0.020 |
| MAP3K8 | 0.505 | 0.517 | -0.012 |
| NOD2 | 0.966 | 0.976 | -0.010 |
| OSMR | 0.758 | 0.763 | -0.005 |
| NFKBIZ | 0.954 | 0.959 | -0.005 |
| IRF8 | 0.026 | 0.029 | -0.003 |
| TRAF3IP2 | 0.887 | 0.889 | -0.002 |
| TLR4 | 0.004 | 0.004 | 0.000 |
| IL2RA | 0.029 | 0.023 | 0.006 |
| TIMD4 | 0.887 | 0.880 | 0.007 |
| IL23R | 0.128 | 0.118 | 0.010 |
| ITGAV | 0.209 | 0.195 | 0.014 |
| CCL20 | 0.449 | 0.421 | 0.028 |
| IL12B | 0.567 | 0.533 | 0.034 |
| SLAMF8 | 0.235 | 0.199 | 0.036 |
| IRGM | 0.865 | 0.827 | 0.038 |
| HLA-DQA1 | 0.140 | 0.102 | 0.038 |
| NFKBIA | 0.291 | 0.251 | 0.040 |
| FCGR2A | 0.796 | 0.753 | 0.043 |
| CCL7 | 0.543 | 0.486 | 0.057 |
| IFNG | 0.174 | 0.113 | 0.061 |
| IFNGR2 | 0.193 | 0.125 | 0.068 |
| CD226 | 0.801 | 0.727 | 0.074 |
| CLEC10A | 0.908 | 0.832 | 0.076 |
| ATG5 | 0.217 | 0.137 | 0.080 |
| IRF5 | 0.732 | 0.640 | 0.092 |
| CSF2RB | 0.846 | 0.720 | 0.126 |
| IL27 | 0.679 | 0.492 | 0.187 |
| PLA2R1 | 0.516 | 0.014 | 0.502 |

**Table S15.** Stratification of post-infection IBD, CD, and UC risk by PRS across infection types.

| **Exposure** | **IBD** | | | **CD** | | | **UC** | | |
| --- | --- | --- | --- | --- | --- | --- | --- | --- | --- |
|  | **Low** | **High** | ***P*-interaction** | **Low** | **High** | ***P*-interaction** | **Low** | **High** | ***P*-interaction** |
|  | **HR (95%CI)** | **HR (95%CI)** |  | **HR (95%CI)** | **HR (95%CI)** |  | **HR (95%CI)** | **HR (95%CI)** |  |
| **All infectious** | 3.90 (3.31, 4.60) | 3.30 (2.90, 3.75) | 0.031 | 6.36 (3.91, 10.35) | 4.85 (3.18, 7.40) | 0.083 | 3.84 (3.19, 4.64) | 3.25 (2.78, 3.79) | 0.244 |
| **Bacterial infections** | 3.79 (3.22, 4.46) | 3.45 (3.03, 3.92) | 0.127 | 5.95 (3.71, 9.54) | 5.16 (3.39, 7.86) | 0.108 | 3.74 (3.11, 4.51) | 3.34 (2.86, 3.89) | 0.444 |
| **By severity** |  |  |  |  |  |  |  |  |  |
| Invasive bacterial infections | 2.42 (2.03, 2.88) | 2.23 (1.93, 2.57) | 0.174 | 3.76 (2.39, 5.93) | 2.96 (1.93, 4.52) | 0.070 | 2.26 (1.84, 2.77) | 2.29 (1.93, 2.72) | 0.858 |
| Localized bacterial infections | 4.05 (3.44, 4.75) | 3.64 (3.20, 4.15) | 0.108 | 4.79 (3.07, 7.47) | 5.34 (3.56, 8.01) | 0.301 | 3.98 (3.31, 4.78) | 3.63 (3.11, 4.23) | 0.571 |
| Bacterial infections with sepsis | 2.14 (1.55, 2.97) | 2.96 (2.33, 3.76) | 0.252 | 4.11 (2.02, 8.38) | 3.80 (1.94, 7.43) | 0.515 | 2.69 (1.89, 3.82) | 2.10 (1.53, 2.90) | 0.372 |
| Bacterial infections without sepsis | 3.82 (3.25, 4.50) | 3.45 (3.04, 3.93) | 0.112 | 5.39 (3.39, 8.58) | 5.23 (3.44, 7.97) | 0.094 | 3.80 (3.16, 4.58) | 3.39 (2.90, 3.95) | 0.441 |
| **By location** |  |  |  |  |  |  |  |  |  |
| Extracellular bacterial infections | 2.65 (2.10, 3.33) | 2.60 (2.15, 3.15) | 0.534 | 1.28 (0.55, 2.97) | 2.95 (1.68, 5.18) | 0.033 | 2.72 (2.08, 3.55) | 2.72 (2.18, 3.40) | 0.902 |
| Intracellular bacterial infections | 3.13 (1.68, 5.86) | 4.66 (2.99, 7.26) | 0.377 | 8.29 (2.59, 26.53) | 6.28 (1.98, 19.94) | 0.870 | 3.18 (1.51, 6.72) | 4.02 (2.32, 6.96) | 0.625 |
| **By Gram stain** |  |  |  |  |  |  |  |  |  |
| Gram-positive infections | 2.29 (1.67, 3.15) | 2.64 (2.06, 3.40) | 0.808 | 1.64 (0.59, 4.51) | 2.55 (1.17, 5.56) | 0.256 | 3.46 (2.52, 4.76) | 2.41 (1.77, 3.27) | 0.128 |
| Gram-negative infections | 3.16 (2.47, 4.03) | 2.72 (2.20, 3.37) | 0.193 | 2.20 (1.00, 4.85) | 3.79 (2.12, 6.76) | 0.383 | 2.85 (2.12, 3.85) | 2.92 (2.28, 3.73) | 0.810 |
| **Viral infections** | 2.17 (1.64, 2.88) | 2.34 (1.87, 2.93) | 0.957 | 2.45 (1.17, 5.12) | 2.57 (1.33, 4.96) | 0.792 | 2.57 (1.89, 3.51) | 2.44 (1.88, 3.17) | 0.874 |
| **By type of viral infection** | |  |  |  |  |  |  |  |  |
| Acute viral infections | 2.37 (1.76, 3.20) | 2.26 (1.76, 2.91) | 0.566 | 1.49 (0.54, 4.08) | 2.63 (1.27, 5.46) | 0.995 | 2.78 (2.00, 3.87) | 2.51 (1.88, 3.33) | 0.717 |
| Herpesvirus (persistent) infections | 2.31 (1.15, 4.63) | 2.87 (1.72, 4.79) | 0.670 | 6.87 (2.15, 21.89) | 3.37 (0.83, 13.76) | 0.302 | 3.20 (1.59, 6.44) | 2.13 (1.06, 4.28) | 0.446 |
| Other persistent viral infections | 0.66 (0.16, 2.63) | 1.65 (0.78, 3.47) | 0.266 | 2.24 (0.31, 16.32) | / | 0.515 | 0.98 (0.24, 3.95) | 1.33 (0.50, 3.55) | 0.733 |
| **Parasitic infections** | 3.34 (1.25, 8.93) | 3.72 (1.67, 8.30) | 0.929 | 9.87 (1.37, 71.24) | 11.44 (2.81, 46.55) | 0.996 | 2.26 (0.56, 9.07) | 3.48 (1.30, 9.31) | 0.590 |
| **Fungal infections** | 2.50 (1.72, 3.64) | 2.64 (1.93, 3.60) | 0.964 | 1.99 (0.62, 6.38) | 3.57 (1.55, 8.23) | 0.098 | 2.09 (1.29, 3.41) | 2.55 (1.76, 3.69) | 0.488 |
| **By location** |  |  |  |  |  |  |  |  |  |
| Superficial fungal infections | 1.90 (1.07, 3.38) | 2.29 (1.46, 3.57) | 0.770 | 1.18 (0.16, 8.53) | 3.19 (1.00, 10.17) | 0.871 | 1.36 (0.61, 3.04) | 2.36 (1.41, 3.94) | 0.235 |
| Deep fungal infections | 3.10 (1.96, 4.90) | 3.08 (2.07, 4.60) | 0.849 | 2.71 (0.66, 11.13) | 3.50 (1.10, 11.10) | 0.117 | 2.86 (1.61, 5.08) | 2.79 (1.72, 4.52) | 0.991 |

CI, confidence interval; HR, hazard ratio; IBD, inflammatory bowel disease; CD, Crohn's disease; UC, ulcerative colitis; PRS, Polygenic Risk Score.

Based on the fully-adjusted model, adjusted for age, sex, ethnic background, education level, TDI, BMI, smoking status, alcohol consumption, physical activity.

**Table S16.** Stratification of post-infection IBD, CD, and UC risk by IIS across infection types.

| **Exposure** | **IBD** | | | **CD** | | | **UC** | | |
| --- | --- | --- | --- | --- | --- | --- | --- | --- | --- |
|  | **Low** | **High** | ***P*-interaction** | **Low** | **High** | ***P*-interaction** | **Low** | **High** | ***P*-interaction** |
|  | **HR (95%CI)** | **HR (95%CI)** |  | **HR (95%CI)** | **HR (95%CI)** |  | **HR (95%CI)** | **HR (95%CI)** |  |
| **All infectious** | 3.15 (2.86, 3.48) | 6.35 (4.83, 8.35) | <0.001 | 4.73 (3.98, 5.63) | 7.59 (5.13, 11.22) | 0.021 | 3.12 (2.79, 3.49) | 6.27 (4.29, 9.18) | <0.001 |
| **Bacterial infections** | 3.26 (2.96, 3.59) | 6.18 (4.73, 8.08) | <0.001 | 4.73 (3.99, 5.62) | 8.48 (5.73, 12.54) | 0.005 | 3.19 (2.86, 3.57) | 6.29 (4.33, 9.14) | <0.001 |
| **By severity** |  |  |  |  |  |  |  |  |  |
| Invasive bacterial infections | 2.18 (1.96, 2.44) | 3.98 (3.08, 5.14) | <0.001 | 2.64 (2.21, 3.16) | 3.77 (2.67, 5.32) | 0.051 | 2.23 (1.97, 2.53) | 4.50 (3.16, 6.41) | <0.001 |
| Localized bacterial infections | 3.59 (3.25, 3.95) | 5.07 (3.94, 6.53) | 0.004 | 4.98 (4.22, 5.89) | 6.95 (4.89, 9.88) | 0.067 | 3.46 (3.10, 3.87) | 5.57 (3.92, 7.93) | 0.006 |
| Bacterial infections with sepsis | 2.75 (2.29, 3.32) | 4.42 (3.00, 6.52) | 0.013 | 3.28 (2.45, 4.38) | 3.78 (2.18, 6.55) | 0.473 | 2.24 (1.77, 2.82) | 9.63 (6.20, 14.97) | <0.001 |
| Bacterial infections without sepsis | 3.27 (2.96, 3.60) | 6.17 (4.72, 8.05) | <0.001 | 4.73 (3.99, 5.62) | 7.96 (5.42, 11.69) | 0.01 | 3.24 (2.90, 3.62) | 5.93 (4.10, 8.57) | 0.001 |
| **By location** |  |  |  |  |  |  |  |  |  |
| Extracellular bacterial infections | 2.31 (1.99, 2.69) | 4.89 (3.62, 6.61) | <0.001 | 2.43 (1.90, 3.11) | 4.74 (3.16, 7.11) | 0.004 | 2.57 (2.18, 3.04) | 4.88 (3.21, 7.43) | 0.003 |
| Intracellular bacterial infections | 4.41 (3.16, 6.14) | 2.97 (1.10, 8.00) | 0.531 | 6.49 (4.05, 10.39) | 5.65 (2.08, 15.32) | 0.815 | 4.12 (2.79, 6.07) | 3.09 (0.76, 12.54) | 0.784 |
| **By Gram stain** |  |  |  |  |  |  |  |  |  |
| Gram-positive infections | 2.24 (1.83, 2.75) | 3.70 (2.46, 5.56) | 0.013 | 2.08 (1.46, 2.95) | 3.35 (1.88, 5.98) | 0.137 | 2.66 (2.14, 3.30) | 3.48 (1.94, 6.24) | 0.291 |
| Gram-negative infections | 2.70 (2.30, 3.18) | 5.43 (3.93, 7.49) | <0.001 | 3.27 (2.55, 4.21) | 5.75 (3.77, 8.77) | 0.019 | 2.90 (2.42, 3.47) | 5.26 (3.34, 8.28) | 0.011 |
| **Viral infections** | 2.04 (1.71, 2.44) | 3.72 (2.61, 5.30) | 0.001 | 2.14 (1.60, 2.85) | 2.88 (1.70, 4.87) | 0.326 | 2.16 (1.77, 2.64) | 5.27 (3.37, 8.22) | <0.001 |
| **By type of viral infection** |  |  |  |  |  |  |  |  |  |
| Acute viral infections | 2.08 (1.71, 2.53) | 3.83 (2.61, 5.61) | 0.002 | 2.06 (1.48, 2.85) | 2.39 (1.28, 4.44) | 0.641 | 2.32 (1.87, 2.87) | 4.88 (2.97, 8.02) | 0.003 |
| Herpesvirus (persistent) infections | 2.28 (1.48, 3.51) | 6.35 (3.26, 12.37) | 0.008 | 3.25 (1.79, 5.91) | 5.74 (2.12, 15.55) | 0.325 | 2.59 (1.62, 4.12) | 8.23 (3.61, 18.78) | 0.012 |
| Other persistent viral infections | 2.30 (1.19, 4.44) | 9.05 (3.36, 24.36) | 0.019 | 1.21 (0.45, 3.23) | 4.33 (1.37, 13.74) | 0.134 | 0.78 (0.32, 1.88) | 2.50 (0.61, 10.22) | 0.1 |
| **Parasitic infections** | 3.34 (1.25, 8.93) | 3.72 (1.67, 8.30) | 0.929 | 5.61 (2.66, 11.84) | 6.03 (1.48, 24.63) | 0.947 | 2.07 (0.92, 4.61) | 7.44 (1.83, 30.24) | 0.088 |
| **Fungal infections** | 2.46 (1.93, 3.13) | 3.70 (2.25, 6.09) | 0.088 | 3.30 (2.33, 4.68) | 5.31 (2.97, 9.47) | 0.151 | 2.12 (1.57, 2.84) | 5.51 (3.01, 10.07) | 0.003 |
| **By location** |  |  |  |  |  |  |  |  |  |
| Superficial fungal infections | 2.51 (1.82, 3.45) | 2.79 (1.31, 5.95) | 0.663 | 3.28 (2.07, 5.20) | 4.47 (1.96, 10.21) | 0.524 | 1.86 (1.22, 2.84) | 6.97 (3.38, 14.36) | 0.001 |
| Deep fungal infections | 2.38 (1.70, 3.33) | 4.40 (2.39, 8.09) | 0.051 | 3.22 (1.98, 5.23) | 5.04 (2.34, 10.86) | 0.274 | 2.31 (1.56, 3.41) | 4.00 (1.62, 9.87) | 0.228 |

CI, confidence interval; HR, hazard ratio; IBD, inflammatory bowel disease; CD, Crohn's disease; UC, ulcerative colitis; IIS, Infection IBD Score.

Based on the fully-adjusted model, adjusted for age, sex, ethnic background, education level, TDI, BMI, smoking status, alcohol consumption, physical activity.

**Table S17.** Absolute risks and infection-associated absolute risk differences for IBD, CD, and UC according to binary-based IIS strata.

| Outcome | Time horizon (years) | IIS group | Absolute risk in uninfected participants | Absolute risk after hospital-treated infection | Absolute risk difference, percentage points | Excess cases per 1,000 participants |
| --- | --- | --- | --- | --- | --- | --- |
| IBD | 1 | Low | 0.03% (0.02%, 0.04%) | 0.22% (0.18%, 0.25%) | 0.188 | 1.876 |
| IBD | 1 | High | 0.02% (0.01%, 0.03%) | 0.29% (0.24%, 0.34%) | 0.270 | 2.701 |
| IBD | 3 | Low | 0.08% (0.07%, 0.10%) | 0.44% (0.40%, 0.49%) | 0.361 | 3.607 |
| IBD | 3 | High | 0.07% (0.05%, 0.08%) | 0.56% (0.49%, 0.63%) | 0.491 | 4.909 |
| IBD | 6 | Low | 0.17% (0.14%, 0.19%) | 0.73% (0.67%, 0.80%) | 0.566 | 5.659 |
| IBD | 6 | High | 0.13% (0.10%, 0.15%) | 0.91% (0.81%, 1.00%) | 0.780 | 7.802 |
| IBD | 9 | Low | 0.28% (0.25%, 0.31%) | 0.97% (0.89%, 1.06%) | 0.696 | 6.964 |
| IBD | 9 | High | 0.19% (0.16%, 0.22%) | 1.22% (1.10%, 1.34%) | 1.028 | 10.277 |
| IBD | 12 | Low | 0.37% (0.34%, 0.40%) | 1.18% (1.09%, 1.28%) | 0.810 | 8.103 |
| IBD | 12 | High | 0.27% (0.23%, 0.30%) | 1.45% (1.32%, 1.59%) | 1.187 | 11.868 |
| CD | 1 | Low | 0.01% (0.00%, 0.02%) | 0.11% (0.09%, 0.14%) | 0.103 | 1.026 |
| CD | 1 | High | 0.00% (0.00%, 0.01%) | 0.12% (0.09%, 0.15%) | 0.119 | 1.192 |
| CD | 3 | Low | 0.03% (0.02%, 0.04%) | 0.21% (0.17%, 0.24%) | 0.176 | 1.763 |
| CD | 3 | High | 0.01% (0.01%, 0.02%) | 0.21% (0.17%, 0.25%) | 0.201 | 2.013 |
| CD | 6 | Low | 0.05% (0.04%, 0.06%) | 0.34% (0.29%, 0.39%) | 0.289 | 2.886 |
| CD | 6 | High | 0.03% (0.02%, 0.04%) | 0.37% (0.32%, 0.43%) | 0.341 | 3.414 |
| CD | 9 | Low | 0.07% (0.06%, 0.09%) | 0.47% (0.41%, 0.53%) | 0.398 | 3.981 |
| CD | 9 | High | 0.06% (0.04%, 0.07%) | 0.49% (0.42%, 0.55%) | 0.430 | 4.301 |
| CD | 12 | Low | 0.11% (0.09%, 0.13%) | 0.56% (0.49%, 0.63%) | 0.454 | 4.538 |
| CD | 12 | High | 0.07% (0.05%, 0.09%) | 0.56% (0.49%, 0.64%) | 0.493 | 4.932 |
| UC | 1 | Low | 0.02% (0.01%, 0.03%) | 0.16% (0.13%, 0.19%) | 0.139 | 1.387 |
| UC | 1 | High | 0.02% (0.01%, 0.03%) | 0.18% (0.14%, 0.21%) | 0.159 | 1.589 |
| UC | 3 | Low | 0.06% (0.05%, 0.07%) | 0.35% (0.31%, 0.40%) | 0.292 | 2.920 |
| UC | 3 | High | 0.05% (0.04%, 0.07%) | 0.35% (0.30%, 0.40%) | 0.296 | 2.958 |
| UC | 6 | Low | 0.13% (0.11%, 0.15%) | 0.55% (0.49%, 0.61%) | 0.424 | 4.242 |
| UC | 6 | High | 0.10% (0.08%, 0.12%) | 0.61% (0.54%, 0.68%) | 0.514 | 5.139 |
| UC | 9 | Low | 0.23% (0.20%, 0.25%) | 0.71% (0.63%, 0.78%) | 0.481 | 4.812 |
| UC | 9 | High | 0.14% (0.12%, 0.16%) | 0.83% (0.74%, 0.92%) | 0.694 | 6.942 |
| UC | 12 | Low | 0.31% (0.27%, 0.34%) | 0.87% (0.78%, 0.95%) | 0.562 | 5.617 |
| UC | 12 | High | 0.19% (0.16%, 0.22%) | 1.02% (0.91%, 1.13%) | 0.829 | 8.289 |

Absolute risks were estimated for participants with and without hospital-treated infection within each IIS stratum at 1, 3, 6, 9, and 12 years. The binary IIS groups were defined as low IIS (IIS ≤0) and high IIS (IIS >0) based on each disease-specific IIS. Absolute risk difference was calculated as the difference in cumulative risk between infected and uninfected participants within the same IIS stratum. Excess cases per 1,000 participants were calculated as the absolute risk difference multiplied by 10. IBD, inflammatory bowel disease; CD, Crohn’s disease; UC, ulcerative colitis; IIS, Infection IBD Score.

**Table S18.** Absolute risks and infection-associated absolute risk differences for IBD, CD, and UC according to binary-based PRS strata.

| Outcome | Time horizon (years) | PRS group | Absolute risk in uninfected participants | Absolute risk after hospital-treated infection | Absolute risk difference, percentage points | Excess cases per 1,000 participants |
| --- | --- | --- | --- | --- | --- | --- |
| IBD | 1 | Low | 0.02% (0.01%, 0.03%) | 0.20% (0.16%, 0.24%) | 0.175 | 1.754 |
| IBD | 1 | High | 0.04% (0.03%, 0.05%) | 0.30% (0.25%, 0.35%) | 0.262 | 2.615 |
| IBD | 3 | Low | 0.05% (0.04%, 0.07%) | 0.40% (0.35%, 0.46%) | 0.353 | 3.529 |
| IBD | 3 | High | 0.11% (0.09%, 0.13%) | 0.57% (0.50%, 0.64%) | 0.465 | 4.652 |
| IBD | 6 | Low | 0.12% (0.09%, 0.14%) | 0.64% (0.57%, 0.72%) | 0.528 | 5.280 |
| IBD | 6 | High | 0.19% (0.16%, 0.22%) | 0.97% (0.88%, 1.07%) | 0.783 | 7.832 |
| IBD | 9 | Low | 0.17% (0.14%, 0.19%) | 0.86% (0.77%, 0.96%) | 0.694 | 6.942 |
| IBD | 9 | High | 0.30% (0.27%, 0.34%) | 1.29% (1.17%, 1.40%) | 0.983 | 9.829 |
| IBD | 12 | Low | 0.23% (0.19%, 0.26%) | 1.08% (0.96%, 1.19%) | 0.849 | 8.494 |
| IBD | 12 | High | 0.42% (0.38%, 0.46%) | 1.48% (1.35%, 1.61%) | 1.057 | 10.567 |
| CD | 1 | Low | 0.01% (0.00%, 0.01%) | 0.08% (0.05%, 0.10%) | 0.074 | 0.737 |
| CD | 1 | High | 0.01% (0.00%, 0.02%) | 0.17% (0.13%, 0.20%) | 0.155 | 1.546 |
| CD | 3 | Low | 0.02% (0.01%, 0.02%) | 0.16% (0.12%, 0.19%) | 0.141 | 1.405 |
| CD | 3 | High | 0.03% (0.02%, 0.04%) | 0.27% (0.23%, 0.32%) | 0.242 | 2.417 |
| CD | 6 | Low | 0.03% (0.02%, 0.04%) | 0.27% (0.22%, 0.32%) | 0.239 | 2.392 |
| CD | 6 | High | 0.06% (0.04%, 0.07%) | 0.46% (0.39%, 0.53%) | 0.403 | 4.029 |
| CD | 9 | Low | 0.04% (0.03%, 0.06%) | 0.36% (0.29%, 0.42%) | 0.314 | 3.140 |
| CD | 9 | High | 0.10% (0.08%, 0.12%) | 0.63% (0.54%, 0.71%) | 0.526 | 5.257 |
| CD | 12 | Low | 0.06% (0.04%, 0.07%) | 0.43% (0.35%, 0.50%) | 0.369 | 3.689 |
| CD | 12 | High | 0.13% (0.11%, 0.16%) | 0.71% (0.62%, 0.80%) | 0.579 | 5.793 |
| UC | 1 | Low | 0.02% (0.01%, 0.02%) | 0.13% (0.09%, 0.16%) | 0.111 | 1.110 |
| UC | 1 | High | 0.03% (0.02%, 0.04%) | 0.21% (0.17%, 0.25%) | 0.184 | 1.845 |
| UC | 3 | Low | 0.04% (0.03%, 0.05%) | 0.31% (0.26%, 0.36%) | 0.268 | 2.681 |
| UC | 3 | High | 0.07% (0.06%, 0.09%) | 0.40% (0.34%, 0.46%) | 0.328 | 3.279 |
| UC | 6 | Low | 0.08% (0.06%, 0.10%) | 0.49% (0.42%, 0.56%) | 0.407 | 4.066 |
| UC | 6 | High | 0.14% (0.12%, 0.16%) | 0.68% (0.60%, 0.76%) | 0.536 | 5.356 |
| UC | 9 | Low | 0.12% (0.10%, 0.14%) | 0.65% (0.57%, 0.74%) | 0.533 | 5.331 |
| UC | 9 | High | 0.22% (0.19%, 0.26%) | 0.89% (0.79%, 0.98%) | 0.661 | 6.608 |
| UC | 12 | Low | 0.18% (0.15%, 0.21%) | 0.80% (0.71%, 0.90%) | 0.624 | 6.238 |
| UC | 12 | High | 0.30% (0.27%, 0.34%) | 1.05% (0.94%, 1.16%) | 0.751 | 7.509 |

Absolute risks were estimated for participants with and without hospital-treated infection within each PRS stratum at 1, 3, 6, 9, and 12 years. The binary PRS groups were defined using the median value of each disease-specific PRS. Absolute risk difference was calculated as the difference in cumulative risk between infected and uninfected participants within the same PRS stratum. Excess cases per 1,000 participants were calculated as the absolute risk difference multiplied by 10. IBD, inflammatory bowel disease; CD, Crohn’s disease; UC, ulcerative colitis; PRS, polygenic risk score.

**Table S19**. Stratification of post-infection IBD, CD, and UC risk by bacterial-specific IIS.

| **Exposure** | **IBD** | | | **CD** | | | **UC** | | |
| --- | --- | --- | --- | --- | --- | --- | --- | --- | --- |
|  | **Low** | **High** | ***P*-interaction** | **Low** | **High** | ***P*-interaction** | **Low** | **High** | ***P*-interaction** |
|  | **HR (95%CI)** | **HR (95%CI)** |  | **HR (95%CI)** | **HR (95%CI)** |  | **HR (95%CI)** | **HR (95%CI)** |  |
| **Bacterial infections** | 3.11 (2.78, 3.47) | 4.59 (3.90, 5.39) | <0.001 | 4.93 (4.18, 5.82) | 8.48 (5.25, 13.72) | 0.018 | 3.02 (2.67, 3.41) | 4.81 (3.87, 5.97) | <0.001 |
| **By severity** |  |  |  |  |  |  |  |  |  |
| Invasive bacterial infections | 2.09 (1.85, 2.37) | 3.04 (2.57, 3.59) | <0.001 | 2.60 (2.19, 3.09) | 4.89 (3.23, 7.39) | 0.003 | 2.18 (1.90, 2.51) | 3.11 (2.49, 3.89) | 0.003 |
| Localized bacterial infections | 3.52 (3.15, 3.93) | 4.28 (3.65, 5.01) | 0.028 | 4.94 (4.20, 5.79) | 9.02 (5.75, 14.13) | 0.006 | 3.28 (2.90, 3.70) | 4.85 (3.92, 5.99) | <0.001 |
| Bacterial infections with sepsis | 2.56 (2.06, 3.18) | 3.87 (2.97, 5.04) | 0.011 | 3.19 (2.40, 4.23) | 4.70 (2.52, 8.74) | 0.167 | 2.13 (1.65, 2.77) | 4.82 (3.48, 6.68) | <0.001 |
| Bacterial infections without sepsis | 3.11 (2.79, 3.48) | 4.60 (3.91, 5.40) | <0.001 | 4.90 (4.15, 5.78) | 8.12 (5.05, 13.04) | 0.024 | 3.04 (2.69, 3.44) | 4.87 (3.92, 6.06) | <0.001 |
| **By location** |  |  |  |  |  |  |  |  |  |
| Extracellular bacterial infections | 2.26 (1.91, 2.69) | 3.40 (2.75, 4.21) | 0.002 | 2.47 (1.95, 3.12) | 5.82 (3.63, 9.32) | 0.001 | 2.57 (2.14, 3.09) | 3.37 (2.53, 4.48) | 0.060 |
| Intracellular bacterial infections | 3.88 (2.63, 5.73) | 4.98 (2.93, 8.46) | 0.427 | 6.71 (4.29, 10.50) | 3.98 (0.98, 16.25) | 0.550 | 3.38 (2.12, 5.39) | 5.99 (3.19, 11.27) | 0.104 |
| **By Gram stain** |  |  |  |  |  |  |  |  |  |
| Gram-positive infections | 2.16 (1.71, 2.73) | 3.02 (2.26, 4.03) | 0.056 | 1.98 (1.40, 2.79) | 4.74 (2.55, 8.79) | 0.010 | 2.75 (2.17, 3.48) | 2.69 (1.81, 4.01) | 0.826 |
| Gram-negative infections | 2.61 (2.17, 3.13) | 4.00 (3.18, 5.02) | 0.003 | 3.42 (2.70, 4.34) | 6.05 (3.64, 10.07) | 0.025 | 2.74 (2.24, 3.36) | 4.22 (3.14, 5.68) | 0.008 |

CI, confidence interval; HR, hazard ratio; IBD, inflammatory bowel disease; CD, Crohn's disease; UC, ulcerative colitis; IIS, Infection IBD Score.

Based on the fully-adjusted model, adjusted for age, sex, ethnic background, education level, TDI, BMI, smoking status, alcohol consumption, physical activity.

**Table S20.** Stratification of post-infection IBD, CD, and UC risk by viral-specific IIS.

| **Exposure** | **IBD** | | | **CD** | | | **UC** | | |
| --- | --- | --- | --- | --- | --- | --- | --- | --- | --- |
|  | **Low** | **High** | ***P*-interaction** | **Low** | **High** | ***P*-interaction** | **Low** | **High** | ***P*-interaction** |
|  | **HR (95%CI)** | **HR (95%CI)** |  | **HR (95%CI)** | **HR (95%CI)** |  | **HR (95%CI)** | **HR (95%CI)** |  |
| **Viral infections** | 2.21 (1.88, 2.59) | 5.55 (2.34, 13.18) | 0.013 | 1.61 (1.14, 2.26) | 4.41 (2.99, 6.50) | <0.001 | 2.36 (1.96, 2.84) | 4.89 (2.25, 10.60) | 0.065 |
| **By type of viral infection** | |  |  |  |  |  |  |  |  |
| Acute viral infections | 2.29 (1.92, 2.73) | 3.56 (1.22, 10.40) | 0.244 | 1.82 (1.27, 2.61) | 3.00 (1.84, 4.89) | 0.117 | 2.47 (2.02, 3.02) | 5.12 (2.26, 11.61) | 0.071 |
| Herpesvirus (persistent) infections | 2.59 (1.77, 3.79) | 13.31 (3.96, 44.65) | 0.006 | 0.98 (0.31, 3.04) | 11.65 (6.47, 20.99) | <0.001 | 2.82 (1.83, 4.34) | 11.98 (3.68, 38.95) | 0.019 |
| Other persistent viral infections | 1.11 (0.61, 2.01) | 8.12 (1.00, 65.89) | 0.047 | 0.68 (0.17, 2.73) | 4.87 (1.98, 11.93) | 0.022 | 1.00 (0.48, 2.11) | 0.00 (0.00, Inf) | 0.982 |

CI, confidence interval; HR, hazard ratio; IBD, inflammatory bowel disease; CD, Crohn's disease; UC, ulcerative colitis; IIS, Infection IBD Score.

Based on the fully-adjusted model, adjusted for age, sex, ethnic background, education level, TDI, BMI, smoking status, alcohol consumption, physical activity.

**Table S21.** Stratification of post-infection IBD, CD, and UC risk by fungal-specific IIS.

| **Exposure** | **IBD** | | | **CD** | | | **UC** | | |
| --- | --- | --- | --- | --- | --- | --- | --- | --- | --- |
|  | **Low** | **High** | **P-interaction** | **Low** | **High** | **P-interaction** | **Low** | **High** | **P-interaction** |
|  | **HR (95%CI)** | **HR (95%CI)** |  | **HR (95%CI)** | **HR (95%CI)** |  | **HR (95%CI)** | **HR (95%CI)** |  |
| **Fungal infections** | 2.30 (1.79, 2.95) | 4.44 (2.89, 6.83) | 0.010 | 2.43 (1.49, 3.96) | 5.24 (3.58, 7.67) | 0.016 | 2.17 (1.63, 2.89) | 6.67 (3.30, 13.46) | 0.003 |
| **By location** | |  |  |  |  |  |  |  |  |
| Superficial fungal infections | 2.25 (1.60, 3.16) | 4.13 (2.31, 7.39) | 0.079 | 2.55 (1.35, 4.79) | 4.65 (2.76, 7.84) | 0.153 | 1.96 (1.31, 2.94) | 7.96 (3.42, 18.50) | 0.002 |
| Deep fungal infections | 2.29 (1.62, 3.23) | 4.72 (2.70, 8.26) | 0.031 | 2.25 (1.11, 4.54) | 5.25 (3.16, 8.71) | 0.055 | 2.30 (1.57, 3.37) | 5.29 (1.92, 14.55) | 0.109 |

CI, confidence interval; HR, hazard ratio; IBD, inflammatory bowel disease; CD, Crohn's disease; UC, ulcerative colitis; IIS, Infection IBD Score.

Based on the fully-adjusted model, adjusted for age, sex, ethnic background, education level, TDI, BMI, smoking status, alcohol consumption, physical activity.

**Table S22.** Dose–response relationship between infection types and risk of IBD and subtypes according to IIS and PRS quintiles.

|  | **IIS** | | | | |  | **PRS** | | | | |  |
| --- | --- | --- | --- | --- | --- | --- | --- | --- | --- | --- | --- | --- |
|  | **0-20%** | **20-40%** | **40-60%** | **60-80%** | **80-100%** | ***P*-trend** | **0-20%** | **20-40%** | **40-60%** | **60-80%** | **80-100%** | ***P*-trend** |
| **IBD** |  |  |  |  |  |  |  |  |  |  |  |  |
| **All infectious** | 2.43 (2.07, 2.85) | 3.27 (2.58, 4.16) | 3.31 (2.63, 4.17) | 4.20 (3.48, 5.06) | 6.35 (4.83, 8.35) | <0.001 | 4.51 (3.39, 5.99) | 3.47 (2.70, 4.46) | 3.47 (2.75, 4.36) | 3.51 (2.82, 4.36) | 3.20 (2.65, 3.86) | 0.064 |
| **Bacterial infections** | 2.50 (2.13, 2.93) | 3.37 (2.65, 4.28) | 3.53 (2.81, 4.44) | 4.27 (3.55, 5.14) | 6.18 (4.73, 8.08) | <0.001 | 4.25 (3.21, 5.62) | 3.59 (2.80, 4.61) | 3.36 (2.67, 4.22) | 3.72 (3.00, 4.62) | 3.33 (2.76, 4.02) | 0.178 |
| **Viral infections** | 1.57 (1.13, 2.19) | 2.17 (1.43, 3.28) | 2.33 (1.58, 3.45) | 2.39 (1.75, 3.28) | 3.72 (2.61, 5.30) | <0.001 | 2.09 (1.28, 3.40) | 2.28 (1.48, 3.51) | 2.42 (1.64, 3.58) | 1.94 (1.29, 2.92) | 2.51 (1.83, 3.43) | 0.803 |
| **Fungal infections** | 2.04 (1.32, 3.17) | 2.61 (1.49, 4.56) | 2.12 (1.16, 3.89) | 3.17 (2.11, 4.76) | 3.70 (2.25, 6.09) | 0.028 | 2.28 (1.17, 4.47) | 2.03 (1.07, 3.84) | 3.06 (1.84, 5.09) | 3.18 (1.94, 5.19) | 2.30 (1.43, 3.69) | 0.820 |
| **Parasitic infections** | 1.11 (0.28, 4.45) | 1.69 (0.24, 12.07) | 4.54 (1.45, 14.22) | 3.61 (1.16, 11.25) | 9.05 (3.36, 24.36) | 0.008 | 2.46 (0.34, 17.54) | 6.37 (2.04, 19.88) | / | 3.36 (0.84, 13.52) | 5.71 (2.13, 15.30) | 0.596 |
| **CD** |  |  |  |  |  |  |  |  |  |  |  |  |
| **All infectious** | 3.77 (2.78, 5.10) | 4.84 (3.66, 6.39) | 6.02 (3.96, 9.15) | 5.73 (3.38, 9.72) | 7.59 (5.13, 11.22) | 0.004 | 6.29 (3.75, 10.52) | 5.73 (3.67, 8.95) | 4.79 (3.19, 7.18) | 5.23 (3.69, 7.41) | 4.21 (3.10, 5.72) | 0.147 |
| **Bacterial infections** | 3.81 (2.82, 5.14) | 4.71 (3.58, 6.20) | 6.16 (4.07, 9.31) | 5.93 (3.52, 9.99) | 8.48 (5.73, 12.54) | <0.001 | 6.17 (3.73, 10.19) | 5.76 (3.71, 8.93) | 4.72 (3.17, 7.04) | 5.27 (3.74, 7.44) | 4.47 (3.29, 6.06) | 0.233 |
| **Viral infections** | 1.35 (0.73, 2.49) | 2.22 (1.40, 3.52) | 2.83 (1.54, 5.20) | 3.11 (1.47, 6.57) | 2.88 (1.70, 4.87) | 0.064 | 2.38 (1.14, 5.00) | 2.10 (1.01, 4.35) | 2.56 (1.36, 4.81) | 1.71 (0.90, 3.27) | 1.95 (1.15, 3.33) | 0.503 |
| **Fungal infections** | 2.36 (1.16, 4.81) | 3.06 (1.70, 5.51) | 6.40 (3.39, 12.06) | 2.41 (0.75, 7.74) | 5.31 (2.97, 9.47) | 0.086 | 4.74 (2.16, 10.42) | 3.52 (1.52, 8.12) | 3.26 (1.42, 7.48) | 3.13 (1.53, 6.42) | 2.66 (1.35, 5.23) | 0.245 |
| **Parasitic infections** | 5.11 (1.27, 20.65) | 6.34 (2.02, 19.86) | / | 13.34 (3.21, 55.37) | 6.03 (1.48, 24.63) | 0.836 | / | 5.30 (0.74, 38.16) | 13.19 (4.16, 41.82) | 3.89 (0.54, 27.88) | 9.96 (3.18, 31.23) | 0.378 |
| **UC** |  |  |  |  |  |  |  |  |  |  |  |  |
| **All infectious** | 2.09 (1.70, 2.56) | 3.15 (2.37, 4.18) | 3.72 (3.05, 4.54) | 4.98 (3.64, 6.80) | 4.52 (3.48, 5.88) | <0.001 | 4.72 (3.41, 6.54) | 4.00 (3.01, 5.32) | 3.14 (2.39, 4.14) | 3.34 (2.60, 4.30) | 2.97 (2.36, 3.73) | 0.017 |
| **Bacterial infections** | 2.15 (1.75, 2.64) | 3.22 (2.42, 4.27) | 3.78 (3.10, 4.61) | 4.89 (3.59, 6.65) | 4.69 (3.61, 6.08) | <0.001 | 4.09 (2.98, 5.62) | 4.07 (3.07, 5.40) | 3.23 (2.45, 4.25) | 3.43 (2.67, 4.40) | 3.08 (2.45, 3.88) | 0.084 |
| **Viral infections** | 1.32 (0.84, 2.07) | 2.28 (1.40, 3.72) | 2.91 (2.12, 3.99) | 2.57 (1.57, 4.20) | 3.59 (2.47, 5.23) | <0.001 | 2.70 (1.63, 4.48) | 2.96 (1.89, 4.62) | 1.69 (0.96, 2.97) | 2.80 (1.90, 4.14) | 2.41 (1.62, 3.59) | 0.739 |
| **Fungal infections** | 1.22 (0.61, 2.47) | 2.83 (1.49, 5.37) | 1.90 (1.09, 3.31) | 3.77 (2.03, 6.99) | 4.01 (2.40, 6.69) | 0.005 | 3.85 (2.07, 7.14) | 1.44 (0.59, 3.52) | 1.79 (0.79, 4.05) | 2.18 (1.19, 4.01) | 2.72 (1.58, 4.67) | 0.755 |
| **Parasitic infections** | 0.94 (0.13, 6.73) | / | 2.61 (0.65, 10.49) | 6.90 (2.18, 21.86) | 4.02 (1.00, 16.17) | 0.049 | / | / | 11.99 (4.45, 32.28) | / | 3.98 (0.99, 16.02) | 0.443 |

CI, confidence interval; HR, hazard ratio; IBD, inflammatory bowel disease; CD, Crohn's disease; UC, ulcerative colitis; IIS, Infection IBD Score; PRS, Polygenic Risk Score.

Based on the fully-adjusted model, adjusted for age, sex, ethnic background, education level, TDI, BMI, smoking status, alcohol consumption, physical activity.

**Table S23.** Dose–response relationship between infection types and risk of IBD and subtypes according to pathogen-specific IIS and PRS quintiles.

|  | **IIS** | | | |  | **PRS** | | | |  |
| --- | --- | --- | --- | --- | --- | --- | --- | --- | --- | --- |
|  | **0-25%** | **25-50%** | **50-75%** | **75-100%** | ***P*-trend** | **0-25%** | **25-50%** | **50-75%** | **75-100%** | ***P*-trend** |
| **IBD** |  |  |  |  |  |  |  |  |  |  |
| **Bacterial infections** | 2.32 (1.96, 2.75) | 3.87 (3.34, 4.49) | 4.49 (3.56, 5.65) | 4.69 (3.74, 5.88) | <0.001 | 4.11 (3.21, 5.27) | 3.54 (2.85, 4.40) | 3.47 (2.85, 4.23) | 3.42 (2.88, 4.07) | 0.156 |
| **Viral infections** | 1.32 (0.91, 1.93) | 1.55 (1.06, 2.26) | 2.75 (2.05, 3.68) | 3.77 (2.88, 4.95) | <0.001 | 2.09 (1.36, 3.22) | 2.18 (1.50, 3.18) | 2.30 (1.63, 3.27) | 2.38 (1.78, 3.20) | 0.752 |
| **Fungal infections** | 1.08 (0.61, 1.91) | 2.46 (1.62, 3.75) | 3.89 (2.65, 5.69) | 4.44 (2.89, 6.83) | <0.001 | 2.28 (1.17, 4.47) | 2.54 (1.71, 3.78) | 3.18 (1.94, 5.19) | 2.30 (1.43, 3.69) | 0.968 |
| **CD** |  |  |  |  |  |  |  |  |  |  |
| **Bacterial infections** | 3.61 (2.75, 4.74) | 4.10 (2.98, 5.65) | 7.64 (5.75, 10.16) | 8.48 (5.25, 13.72) | <0.001 | 6.68 (4.28, 10.42) | 5.53 (3.73, 8.18) | 4.55 (3.33, 6.22) | 4.71 (3.56, 6.23) | 0.164 |
| **Viral infections** | 1.56 (1.02, 2.39) | 1.70 (0.96, 2.99) | 3.25 (1.88, 5.63) | 6.63 (3.78, 11.62) | <0.001 | 2.36 (1.22, 4.57) | 1.80 (0.90, 3.56) | 1.92 (1.09, 3.39) | 2.21 (1.39, 3.51) | 0.951 |
| **Fungal infections** | 1.46 (0.46, 4.62) | 2.83 (1.64, 4.86) | 4.11 (2.59, 6.51) | 11.72 (5.81, 23.64) | <0.001 | 4.74 (2.16, 10.42) | 3.37 (1.87, 6.08) | 3.13 (1.53, 6.42) | 2.66 (1.35, 5.23) | 0.267 |
| **UC** |  |  |  |  |  |  |  |  |  |  |
| **Bacterial infections** | 2.23 (1.87, 2.66) | 3.69 (2.91, 4.68) | 4.57 (3.77, 5.56) | 4.87 (3.57, 6.63) | <0.001 | 3.93 (2.98, 5.19) | 3.59 (2.79, 4.61) | 3.74 (2.97, 4.71) | 3.01 (2.45, 3.71) | 0.144 |
| **Viral infections** | 1.54 (1.06, 2.23) | 2.82 (2.07, 3.86) | 3.13 (2.21, 4.42) | 2.88 (1.80, 4.60) | 0.018 | 2.70 (1.63, 4.48) | 2.96 (1.89, 4.62) | 2.32 (1.68, 3.19) | 2.41 (1.62, 3.59) | 0.462 |
| **Fungal infections** | 1.28 (0.78, 2.10) | 2.97 (1.89, 4.67) | 3.96 (2.24, 6.99) | 6.67 (3.30, 13.46) | <0.001 | 3.85 (2.07, 7.14) | 1.60 (0.88, 2.92) | 2.18 (1.19, 4.01) | 2.72 (1.58, 4.67) | 0.785 |

CI, confidence interval; HR, hazard ratio; IBD, inflammatory bowel disease; CD, Crohn's disease; UC, ulcerative colitis; IIS, Infection IBD Score; PRS, Polygenic Risk Score.

Based on the fully-adjusted model, adjusted for age, sex, ethnic background, education level, TDI, BMI, smoking status, alcohol consumption, physical activity.

**Table S24.** Stratification of post-infection IBD, CD, and UC risk by reduced IIS after excluding low-evidence SNPs across infection types.

| **Exposure** | **IBD** | | | **CD** | | | **UC** | | |
| --- | --- | --- | --- | --- | --- | --- | --- | --- | --- |
|  | **Low** | **High** | ***P*-interaction** | **Low** | **High** | ***P*-interaction** | **Low** | **High** | ***P*-interaction** |
|  | **HR (95%CI)** | **HR (95%CI)** |  | **HR (95%CI)** | **HR (95%CI)** |  | **HR (95%CI)** | **HR (95%CI)** |  |
| **All infectious** | 2.89 (2.59, 3.23) | 4.82 (4.11, 5.66) | <0.001 | 4.81 (3.95, 5.85) | 5.81 (4.45, 7.58) | 0.194 | 3.13 (2.79, 3.50) | 5.48 (3.90, 7.68) | 0.001 |
| **Bacterial infections** | 2.99 (2.68, 3.35) | 4.86 (4.15, 5.70) | <0.001 | 4.72 (3.88, 5.72) | 6.36 (4.88, 8.29) | 0.052 | 3.20 (2.86, 3.58) | 5.43 (3.89, 7.58) | 0.002 |
| **By severity** |  |  |  |  |  |  |  |  |  |
| Invasive bacterial infections | 1.99 (1.75, 2.26) | 3.23 (2.75, 3.79) | <0.001 | 2.51 (2.05, 3.08) | 3.44 (2.67, 4.42) | 0.044 | 2.27 (2.00, 2.57) | 3.62 (2.61, 5.03) | 0.004 |
| Localized bacterial infections | 3.35 (2.99, 3.75) | 4.65 (3.98, 5.42) | <0.001 | 5.02 (4.15, 6.06) | 5.84 (4.55, 7.49) | 0.253 | 3.46 (3.09, 3.87) | 5.16 (3.75, 7.11) | 0.01 |
| Bacterial infections with sepsis | 2.41 (1.92, 3.02) | 4.07 (3.17, 5.22) | 0.001 | 3.60 (2.61, 4.97) | 3.04 (1.98, 4.66) | 0.732 | 2.49 (1.99, 3.12) | 5.26 (3.27, 8.46) | 0.002 |
| Bacterial infections without sepsis | 3.02 (2.70, 3.38) | 4.80 (4.10, 5.63) | <0.001 | 4.70 (3.87, 5.70) | 6.23 (4.79, 8.10) | 0.064 | 3.24 (2.90, 3.62) | 5.35 (3.84, 7.46) | 0.002 |
| **By location** |  |  |  |  |  |  |  |  |  |
| Extracellular bacterial infections | 2.20 (1.85, 2.62) | 3.46 (2.82, 4.25) | <0.001 | 2.36 (1.78, 3.13) | 3.70 (2.70, 5.06) | 0.024 | 2.59 (2.20, 3.06) | 4.27 (2.86, 6.37) | 0.011 |
| Intracellular bacterial infections | 3.37 (2.19, 5.20) | 5.76 (3.64, 9.11) | 0.071 | 8.34 (5.12, 13.57) | 3.41 (1.40, 8.27) | 0.109 | 4.20 (2.85, 6.20) | 2.59 (0.64, 10.50) | 0.589 |
| **By Gram stain** |  |  |  |  |  |  |  |  |  |
| Gram-positive infections | 2.09 (1.64, 2.66) | 3.09 (2.35, 4.08) | 0.022 | 2.24 (1.52, 3.29) | 2.47 (1.54, 3.96) | 0.648 | 2.67 (2.15, 3.32) | 3.20 (1.83, 5.60) | 0.372 |
| Gram-negative infections | 2.46 (2.03, 2.99) | 4.19 (3.38, 5.20) | <0.001 | 3.13 (2.34, 4.18) | 4.76 (3.45, 6.56) | 0.038 | 2.97 (2.48, 3.55) | 4.24 (2.72, 6.63) | 0.087 |
| **Viral infections** | 1.93 (1.56, 2.38) | 2.91 (2.28, 3.71) |  | 1.96 (1.40, 2.76) | 2.80 (1.91, 4.12) |  | 2.26 (1.85, 2.75) | 3.85 (2.46, 6.04) |  |
| **By type of viral infection** |  |  | 0.006 |  |  | 0.162 |  |  | 0.016 |
| Acute viral infections | 1.91 (1.51, 2.42) | 3.07 (2.37, 3.98) | 0.004 | 2.17 (1.51, 3.12) | 2.01 (1.24, 3.26) | 0.884 | 2.41 (1.95, 2.98) | 3.68 (2.24, 6.05) | 0.073 |
| Herpesvirus (persistent) infections | 2.60 (1.63, 4.15) | 3.20 (1.81, 5.68) | 0.512 | 2.64 (1.25, 5.57) | 5.73 (2.83, 11.60) | 0.13 | 2.80 (1.78, 4.40) | 5.70 (2.33, 13.95) | 0.142 |
| Other persistent viral infections | 1.15 (0.57, 2.31) | 1.31 (0.49, 3.52) | 0.78 | 0.38 (0.05, 2.69) | 4.35 (1.93, 9.85) | 0.032 | 0.94 (0.42, 2.10) | 1.21 (0.17, 8.74) | 0.739 |
| **Parasitic infections** | 1.90 (0.85, 4.25) | 5.97 (2.83, 12.59) | 0.031 | 7.85 (3.72, 16.60) | 2.84 (0.70, 11.48) | 0.211 | 2.47 (1.17, 5.20) | 3.01 (0.42, 21.54) | 0.795 |
| **Fungal infections** | 2.16 (1.61, 2.90) | 3.56 (2.58, 4.91) | 0.016 | 3.02 (1.99, 4.56) | 4.80 (3.11, 7.41) | 0.12 | 2.21 (1.65, 2.96) | 4.14 (2.22, 7.70) | 0.055 |
| **By location** |  |  |  |  |  |  |  |  |  |
| Superficial fungal infections | 1.84 (1.21, 2.82) | 3.93 (2.61, 5.93) | 0.008 | 2.95 (1.69, 5.13) | 4.40 (2.45, 7.89) | 0.322 | 2.17 (1.46, 3.23) | 3.45 (1.41, 8.46) | 0.299 |
| Deep fungal infections | 2.52 (1.73, 3.67) | 2.97 (1.85, 4.75) | 0.51 | 3.15 (1.81, 5.49) | 4.34 (2.37, 7.98) | 0.414 | 2.26 (1.52, 3.36) | 4.18 (1.83, 9.52) | 0.162 |

CI, confidence interval; HR, hazard ratio; IBD, inflammatory bowel disease; CD, Crohn's disease; UC, ulcerative colitis; IIS, Infection IBD Score.

Based on the fully-adjusted model, adjusted for age, sex, ethnic background, education level, TDI, BMI, smoking status, alcohol consumption, physical activity.

**Subgroup analysis**

**Table S25.** Subgroup analysis for associations of infection with incident of inflammatory bowel disease stratified by age, sex, body mass index, and smoking status.

| **Exposure** | **Age<60** | **Age>=60** | ***P*-interaction** | **BMI<25** | **BMI>=25** | ***P*-interaction** | **Female** | **Male** | ***P*-interaction** | **Never Somke** | **Previous Somke** | **Current Somke** | ***P*-interaction** |
| --- | --- | --- | --- | --- | --- | --- | --- | --- | --- | --- | --- | --- | --- |
|  | **HR (95%CI)** | **HR (95%CI)** |  | **HR (95%CI)** | **HR (95%CI)** |  | **HR (95%CI)** | **HR (95%CI)** |  | **HR (95%CI)** | **HR (95%CI)** | **HR (95%CI)** |  |
| **All infectious** | 3.49 (3.09, 3.95) | 3.38 (2.96, 3.87) | 0.463 | 3.41 (2.90, 4.01) | 3.46 (3.10, 3.86) | 0.803 | 3.55 (3.12, 4.03) | 3.37 (2.96, 3.83) | 0.201 | 3.36 (2.93, 3.85) | 3.41 (2.97, 3.93) | 3.85 (3.01, 4.93) | 0.835 |
| **Bacterial infections** | 3.62 (3.21, 4.10) | 3.45 (3.02, 3.94) | 0.344 | 3.51 (2.98, 4.13) | 3.56 (3.20, 3.96) | 0.793 | 3.70 (3.26, 4.20) | 3.41 (3.00, 3.88) | 0.108 | 3.42 (2.99, 3.92) | 3.54 (3.08, 4.07) | 3.99 (3.13, 5.09) | 0.807 |
| **By severity** |  |  |  |  |  |  |  |  |  |  |  |  |  |
| Invasive bacterial infections | 2.32 (2.01, 2.67) | 2.43 (2.11, 2.78) | 0.920 | 2.42 (1.99, 2.93) | 2.36 (2.10, 2.65) | 0.836 | 2.47 (2.15, 2.84) | 2.27 (1.97, 2.62) | 0.110 | 2.25 (1.92, 2.64) | 2.48 (2.14, 2.88) | 2.39 (1.88, 3.05) | 0.707 |
| Localized bacterial infections | 4.01 (3.54, 4.53) | 3.53 (3.10, 4.02) | 0.090 | 3.80 (3.23, 4.49) | 3.76 (3.38, 4.18) | 0.968 | 3.88 (3.42, 4.40) | 3.71 (3.26, 4.21) | 0.212 | 3.63 (3.17, 4.17) | 3.76 (3.28, 4.32) | 4.30 (3.40, 5.43) | 0.710 |
| Bacterial infections with sepsis | 3.56 (2.77, 4.57) | 2.73 (2.20, 3.39) | 0.078 | 4.21 (3.09, 5.74) | 2.73 (2.25, 3.31) | 0.024 | 3.23 (2.54, 4.10) | 2.89 (2.30, 3.62) | 0.228 | 3.10 (2.36, 4.07) | 2.96 (2.32, 3.76) | 3.18 (2.14, 4.73) | 0.866 |
| Bacterial infections without sepsis | 3.60 (3.19, 4.07) | 3.49 (3.06, 3.99) | 0.454 | 3.54 (3.01, 4.16) | 3.56 (3.20, 3.96) | 0.860 | 3.70 (3.26, 4.19) | 3.43 (3.02, 3.90) | 0.123 | 3.46 (3.02, 3.96) | 3.54 (3.08, 4.07) | 3.92 (3.08, 5.00) | 0.892 |
| **By location** |  |  |  |  |  |  |  |  |  |  |  |  |  |
| Extracellular bacterial infections | 2.81 (2.31, 3.42) | 2.49 (2.08, 2.97) | 0.252 | 3.27 (2.55, 4.20) | 2.44 (2.09, 2.85) | 0.064 | 2.54 (2.10, 3.06) | 2.70 (2.24, 3.24) | 0.897 | 2.61 (2.11, 3.23) | 2.76 (2.28, 3.35) | 2.32 (1.66, 3.23) | 0.436 |
| Intracellular bacterial infections | 4.66 (3.05, 7.12) | 3.58 (2.24, 5.71) | 0.336 | 4.88 (2.61, 9.14) | 3.92 (2.73, 5.64) | 0.466 | 5.07 (3.29, 7.82) | 3.38 (2.14, 5.33) | 0.163 | 4.65 (2.87, 7.53) | 3.86 (2.39, 6.25) | 3.72 (1.66, 8.37) | 0.778 |
| **By Gram stain** |  |  |  |  |  |  |  |  |  |  |  |  |  |
| Gram-positive infections | 2.66 (2.06, 3.45) | 2.23 (1.74, 2.86) | 0.238 | 2.78 (1.94, 3.98) | 2.34 (1.91, 2.88) | 0.372 | 2.40 (1.84, 3.14) | 2.45 (1.92, 3.13) | 0.723 | 2.88 (2.18, 3.81) | 2.07 (1.56, 2.75) | 2.45 (1.61, 3.74) | 0.205 |
| Gram-negative infections | 3.41 (2.76, 4.21) | 2.82 (2.33, 3.40) | 0.130 | 3.65 (2.77, 4.80) | 2.89 (2.46, 3.40) | 0.197 | 3.30 (2.73, 4.00) | 2.77 (2.24, 3.41) | 0.088 | 2.95 (2.34, 3.72) | 3.44 (2.81, 4.21) | 2.29 (1.56, 3.36) | 0.086 |
| **Viral infections** | 2.33 (1.88, 2.89) | 2.15 (1.71, 2.69) | 0.464 | 2.11 (1.54, 2.90) | 2.29 (1.92, 2.75) | 0.681 | 1.88 (1.50, 2.37) | 2.65 (2.14, 3.28) | 0.072 | 2.57 (2.02, 3.27) | 1.89 (1.46, 2.44) | 2.44 (1.71, 3.46) | 0.211 |
| **By type of viral infection** |  |  |  |  |  |  |  |  |  |  |  |  |  |
| Acute viral infections | 2.41 (1.90, 3.07) | 2.15 (1.67, 2.75) | 0.397 | 2.02 (1.40, 2.93) | 2.37 (1.95, 2.88) | 0.473 | 1.87 (1.44, 2.42) | 2.72 (2.16, 3.44) | 0.069 | 2.84 (2.20, 3.66) | 1.67 (1.24, 2.26) | 2.60 (1.77, 3.83) | 0.024 |
| Herpesvirus (persistent) infections | 3.77 (2.37, 6.02) | 2.23 (1.32, 3.79) | 0.130 | 3.64 (1.95, 6.80) | 2.66 (1.75, 4.06) | 0.444 | 3.04 (1.91, 4.85) | 2.73 (1.61, 4.64) | 0.675 | 1.34 (0.60, 3.00) | 4.11 (2.63, 6.40) | 3.50 (1.56, 7.86) | 0.036 |
| Other persistent viral infections | 1.01 (0.50, 2.02) | 1.41 (0.53, 3.78) | 0.692 | 1.54 (0.69, 3.47) | 0.88 (0.40, 1.97) | 0.319 | 0.84 (0.35, 2.03) | 1.52 (0.72, 3.21) | 0.335 | 1.87 (0.84, 4.19) | 0.77 (0.25, 2.41) | 0.88 (0.28, 2.74) | 0.398 |
| **Parasitic infections** | 3.05 (1.52, 6.14) | 2.61 (1.08, 6.30) | 0.733 | 1.91 (0.48, 7.65) | 3.20 (1.76, 5.80) | 0.517 | 3.48 (1.65, 7.34) | 2.36 (1.05, 5.27) | 0.511 | 3.92 (1.95, 7.90) | 1.18 (0.29, 4.73) | 3.71 (1.19, 11.59) | 0.236 |
| **Fungal infections** | 2.69 (1.93, 3.74) | 2.60 (1.97, 3.45) | 0.756 | 2.92 (1.95, 4.38) | 2.54 (1.97, 3.27) | 0.598 | 2.86 (2.15, 3.80) | 2.37 (1.71, 3.29) | 0.238 | 2.59 (1.81, 3.72) | 2.60 (1.88, 3.59) | 2.78 (1.74, 4.45) | 0.998 |
| **By type of Fungal infections** |  |  |  |  |  |  |  |  |  |  |  |  |  |
| Superficial fungal infections | 2.45 (1.55, 3.86) | 2.52 (1.71, 3.70) | 0.965 | 2.67 (1.47, 4.85) | 2.45 (1.75, 3.43) | 0.812 | 2.81 (1.94, 4.08) | 2.06 (1.27, 3.34) | 0.199 | 2.29 (1.37, 3.81) | 2.37 (1.50, 3.74) | 3.06 (1.71, 5.48) | 0.825 |
| Deep fungal infections | 3.08 (1.99, 4.75) | 2.53 (1.72, 3.71) | 0.449 | 2.98 (1.78, 4.98) | 2.63 (1.86, 3.72) | 0.745 | 2.97 (2.01, 4.40) | 2.49 (1.63, 3.80) | 0.414 | 2.93 (1.84, 4.69) | 2.74 (1.79, 4.19) | 2.36 (1.16, 4.77) | 0.73 |

CI, confidence interval; HR, hazard ratio.

Based on the fully-adjusted model, adjusted for age, sex, ethnic background, education level, TDI, BMI, smoking status, alcohol consumption, physical activity.

**Table S26**. Subgroup analysis for associations of infection with incident of crohn's disease stratified by age, sex, body mass index, and smoking status.

| **Exposure** | **Age<60** | **Age>=60** | ***P*-interaction** | **BMI<25** | **BMI>=25** | ***P*-interaction** | **Female** | **Male** | ***P*-interaction** | **Never Somke** | **Previous Somke** | **Current Somke** | ***P*-interaction** |
| --- | --- | --- | --- | --- | --- | --- | --- | --- | --- | --- | --- | --- | --- |
|  | **HR (95%CI)** | **HR (95%CI)** |  | **HR (95%CI)** | **HR (95%CI)** |  | **HR (95%CI)** | **HR (95%CI)** |  | **HR (95%CI)** | **HR (95%CI)** | **HR (95%CI)** |  |
| **All infectious** | 4.76 (3.88, 5.84) | 5.53 (4.35, 7.03) | 0.820 | 5.34 (4.04, 7.05) | 5.01 (4.16, 6.04) | 0.787 | 4.89 (3.98, 6.01) | 5.37 (4.24, 6.81) | 0.751 | 4.35 (3.45, 5.48) | 6.43 (4.96, 8.35) | 4.59 (3.18, 6.63) | 0.085 |
| **Bacterial infections** | 4.87 (3.97, 5.96) | 5.65 (4.46, 7.16) | 0.845 | 5.48 (4.16, 7.22) | 5.11 (4.25, 6.14) | 0.759 | 5.10 (4.15, 6.25) | 5.36 (4.24, 6.77) | 0.971 | 4.48 (3.56, 5.63) | 6.44 (4.99, 8.32) | 4.81 (3.35, 6.90) | 0.126 |
| **By severity** |  |  |  |  |  |  |  |  |  |  |  |  |  |
| Invasive bacterial infections | 2.53 (2.03, 3.16) | 3.17 (2.54, 3.95) | 0.645 | 2.88 (2.13, 3.91) | 2.82 (2.35, 3.38) | 0.909 | 3.03 (2.46, 3.73) | 2.56 (2.02, 3.24) | 0.175 | 2.67 (2.07, 3.44) | 3.37 (2.65, 4.29) | 2.17 (1.53, 3.08) | 0.065 |
| Localized bacterial infections | 5.30 (4.35, 6.47) | 5.28 (4.23, 6.59) | 0.461 | 5.96 (4.55, 7.81) | 5.08 (4.26, 6.05) | 0.395 | 5.00 (4.10, 6.09) | 5.75 (4.60, 7.20) | 0.540 | 4.74 (3.78, 5.95) | 5.80 (4.57, 7.36) | 5.69 (4.02, 8.05) | 0.588 |
| Bacterial infections with sepsis | 3.70 (2.54, 5.40) | 3.42 (2.45, 4.77) | 0.401 | 4.46 (2.72, 7.29) | 3.29 (2.47, 4.39) | 0.283 | 3.53 (2.48, 5.02) | 3.43 (2.40, 4.89) | 0.726 | 3.82 (2.54, 5.75) | 3.43 (2.34, 5.02) | 3.12 (1.78, 5.49) | 0.614 |
| Bacterial infections without sepsis | 4.73 (3.86, 5.79) | 5.75 (4.54, 7.28) | 0.631 | 5.57 (4.23, 7.33) | 5.01 (4.17, 6.02) | 0.606 | 5.01 (4.09, 6.15) | 5.36 (4.25, 6.76) | 0.887 | 4.55 (3.62, 5.72) | 6.32 (4.91, 8.15) | 4.55 (3.18, 6.51) | 0.139 |
| **By location** |  |  |  |  |  |  |  |  |  |  |  |  |  |
| Extracellular bacterial infections | 2.95 (2.20, 3.97) | 2.75 (2.07, 3.66) | 0.344 | 3.19 (2.11, 4.81) | 2.76 (2.17, 3.49) | 0.550 | 2.67 (2.01, 3.55) | 2.98 (2.21, 4.02) | 0.779 | 2.30 (1.59, 3.31) | 3.48 (2.58, 4.71) | 2.56 (1.62, 4.03) | 0.210 |
| Intracellular bacterial infections | 5.99 (3.28, 10.93) | 6.26 (3.43, 11.45) | 0.928 | 8.32 (3.68, 18.79) | 5.60 (3.40, 9.22) | 0.393 | 8.80 (5.24, 14.77) | 3.61 (1.71, 7.66) | 0.041 | 8.53 (4.65, 15.64) | 5.34 (2.64, 10.80) | 3.87 (1.23, 12.17) | 0.316 |
| **By Gram stain** |  |  |  |  |  |  |  |  |  |  |  |  |  |
| Gram-positive infections | 2.59 (1.73, 3.87) | 2.03 (1.31, 3.13) | 0.184 | 1.80 (0.88, 3.66) | 2.48 (1.79, 3.43) | 0.470 | 2.31 (1.52, 3.50) | 2.25 (1.48, 3.43) | 0.781 | 2.12 (1.25, 3.57) | 2.01 (1.24, 3.26) | 3.04 (1.77, 5.23) | 0.579 |
| Gram-negative infections | 3.91 (2.87, 5.33) | 3.63 (2.73, 4.84) | 0.373 | 4.44 (2.92, 6.74) | 3.56 (2.79, 4.54) | 0.395 | 4.08 (3.10, 5.36) | 3.22 (2.30, 4.49) | 0.218 | 3.28 (2.28, 4.72) | 5.10 (3.79, 6.88) | 2.10 (1.20, 3.68) | 0.006 |
| **Viral infections** | 2.59 (1.88, 3.58) | 1.97 (1.34, 2.91) | 0.138 | 2.13 (1.28, 3.56) | 2.38 (1.80, 3.17) | 0.705 | 2.12 (1.51, 2.97) | 2.54 (1.76, 3.67) | 0.572 | 2.85 (1.95, 4.16) | 2.06 (1.37, 3.12) | 1.94 (1.13, 3.32) | 0.365 |
| **By type of viral infection** |  |  |  |  |  |  |  |  |  |  |  |  |  |
| Acute viral infections | 2.55 (1.77, 3.67) | 1.61 (1.01, 2.57) | 0.056 | 1.59 (0.81, 3.11) | 2.28 (1.66, 3.14) | 0.332 | 2.05 (1.40, 3.00) | 2.13 (1.38, 3.29) | 0.997 | 2.83 (1.86, 4.31) | 1.61 (0.97, 2.68) | 1.84 (0.99, 3.42) | 0.202 |
| Herpesvirus (persistent) infections | 4.60 (2.37, 8.91) | 3.54 (1.76, 7.15) | 0.498 | 5.84 (2.59, 13.16) | 3.43 (1.89, 6.24) | 0.315 | 4.03 (2.15, 7.56) | 3.89 (1.84, 8.24) | 0.902 | 1.80 (0.58, 5.61) | 6.30 (3.44, 11.53) | 3.58 (1.14, 11.25) | 0.115 |
| Other persistent viral infections | 1.52 (0.63, 3.68) | 1.89 (0.47, 7.59) | 0.914 | 2.05 (0.65, 6.46) | 1.43 (0.53, 3.83) | 0.688 | 0.39 (0.05, 2.78) | 3.68 (1.63, 8.34) | 0.013 | 3.49 (1.30, 9.38) | 1.47 (0.36, 5.90) | 0.61 (0.09, 4.40) | 0.205 |
| **Parasitic infections** | 6.00 (2.67, 13.49) | 4.29 (1.37, 13.41) | 0.620 | 2.68 (0.38, 19.16) | 6.16 (3.06, 12.43) | 0.405 | 4.85 (1.81, 13.02) | 5.84 (2.40, 14.18) | 0.728 | 5.62 (2.08, 15.18) | 3.47 (0.86, 13.97) | 7.57 (2.40, 23.83) | 0.695 |
| **Fungal infections** | 3.15 (1.98, 5.02) | 4.12 (2.81, 6.04) | 0.647 | 4.39 (2.54, 7.59) | 3.45 (2.43, 4.89) | 0.488 | 3.42 (2.29, 5.10) | 3.93 (2.54, 6.09) | 0.757 | 3.82 (2.33, 6.26) | 3.94 (2.52, 6.18) | 2.96 (1.55, 5.66) | 0.558 |
| **By type of Fungal infections** |  |  |  |  |  |  |  |  |  |  |  |  |  |
| Superficial fungal infections | 3.18 (1.74, 5.80) | 3.61 (2.11, 6.18) | 0.952 | 3.79 (1.68, 8.58) | 3.33 (2.10, 5.27) | 0.771 | 3.61 (2.18, 5.97) | 2.99 (1.53, 5.82) | 0.581 | 3.61 (1.85, 7.04) | 3.41 (1.81, 6.44) | 3.01 (1.32, 6.85) | 0.875 |
| Deep fungal infections | 3.04 (1.56, 5.90) | 4.20 (2.54, 6.95) | 0.579 | 4.22 (2.07, 8.60) | 3.47 (2.13, 5.63) | 0.695 | 2.93 (1.61, 5.35) | 4.58 (2.67, 7.85) | 0.330 | 3.93 (2.02, 7.65) | 4.21 (2.36, 7.53) | 2.44 (0.90, 6.63) | 0.424 |

CI, confidence interval; HR, hazard ratio.

Based on the fully-adjusted model, adjusted for age, sex, ethnic background, education level, TDI, BMI, smoking status, alcohol consumption, physical activity.

**Table S27.** Subgroup analysis for associations of infection with incident of ulcerative colitis stratified by age, sex, body mass index, and smoking status.

| **Exposure** | **Age<60** | **Age>=60** | ***P*-interaction** | **BMI<25** | **BMI>=25** | ***P*-interaction** | **Female** | **Male** | ***P*-interaction** | **Never Somke** | **Previous Somke** | **Current Somke** | ***P*-interaction** |
| --- | --- | --- | --- | --- | --- | --- | --- | --- | --- | --- | --- | --- | --- |
|  | **HR (95%CI)** | **HR (95%CI)** |  | **HR (95%CI)** | **HR (95%CI)** |  | **HR (95%CI)** | **HR (95%CI)** |  | **HR (95%CI)** | **HR (95%CI)** | **HR (95%CI)** |  |
| **All infectious** | 3.49 (3.02, 4.04) | 3.17 (2.72, 3.70) | 0.336 | 3.18 (2.63, 3.85) | 3.40 (3.00, 3.86) | 0.558 | 3.58 (3.08, 4.16) | 3.16 (2.72, 3.66) | 0.097 | 3.40 (2.90, 3.99) | 3.04 (2.59, 3.56) | 4.51 (3.30, 6.16) | 0.126 |
| **Bacterial infections** | 3.58 (3.10, 4.14) | 3.23 (2.78, 3.76) | 0.299 | 3.25 (2.68, 3.93) | 3.48 (3.07, 3.94) | 0.537 | 3.70 (3.19, 4.31) | 3.19 (2.76, 3.70) | 0.058 | 3.42 (2.92, 4.01) | 3.19 (2.72, 3.74) | 4.46 (3.28, 6.05) | 0.247 |
| **By severity** |  |  |  |  |  |  |  |  |  |  |  |  |  |
| Invasive bacterial infections | 2.52 (2.13, 2.98) | 2.29 (1.95, 2.69) | 0.323 | 2.36 (1.89, 2.96) | 2.41 (2.11, 2.76) | 0.931 | 2.48 (2.10, 2.93) | 2.34 (1.99, 2.75) | 0.291 | 2.26 (1.87, 2.72) | 2.45 (2.07, 2.91) | 2.70 (2.01, 3.63) | 0.875 |
| Localized bacterial infections | 3.94 (3.41, 4.56) | 3.36 (2.89, 3.91) | 0.139 | 3.52 (2.90, 4.27) | 3.69 (3.26, 4.18) | 0.634 | 4.04 (3.47, 4.69) | 3.35 (2.89, 3.89) | 0.025 | 3.59 (3.06, 4.22) | 3.47 (2.96, 4.07) | 4.65 (3.48, 6.22) | 0.336 |
| Bacterial infections with sepsis | 3.81 (2.84, 5.11) | 2.29 (1.75, 2.99) | 0.014 | 3.79 (2.60, 5.53) | 2.57 (2.04, 3.24) | 0.097 | 2.85 (2.10, 3.86) | 2.84 (2.18, 3.68) | 0.674 | 3.07 (2.23, 4.24) | 2.67 (2.00, 3.56) | 2.89 (1.74, 4.79) | 0.679 |
| Bacterial infections without sepsis | 3.61 (3.12, 4.18) | 3.27 (2.81, 3.80) | 0.313 | 3.27 (2.70, 3.95) | 3.52 (3.10, 3.99) | 0.511 | 3.74 (3.22, 4.35) | 3.22 (2.78, 3.73) | 0.056 | 3.45 (2.94, 4.04) | 3.21 (2.74, 3.77) | 4.52 (3.33, 6.14) | 0.235 |
| **By location** |  |  |  |  |  |  |  |  |  |  |  |  |  |
| Extracellular bacterial infections | 3.14 (2.51, 3.93) | 2.49 (2.03, 3.06) | 0.134 | 3.60 (2.71, 4.77) | 2.51 (2.10, 3.01) | 0.045 | 2.87 (2.31, 3.56) | 2.67 (2.16, 3.30) | 0.381 | 2.87 (2.25, 3.66) | 2.76 (2.21, 3.45) | 2.57 (1.73, 3.82) | 0.693 |
| Intracellular bacterial infections | 5.10 (3.15, 8.27) | 2.89 (1.59, 5.24) | 0.118 | 3.94 (1.76, 8.84) | 3.94 (2.58, 6.01) | 0.908 | 4.19 (2.37, 7.42) | 3.76 (2.29, 6.17) | 0.727 | 3.74 (2.00, 7.01) | 3.89 (2.24, 6.75) | 4.71 (1.93, 11.47) | 0.956 |
| **By Gram stain** |  |  |  |  |  |  |  |  |  |  |  |  |  |
| Gram-positive infections | 3.09 (2.31, 4.14) | 2.44 (1.85, 3.22) | 0.249 | 3.35 (2.28, 4.93) | 2.56 (2.02, 3.23) | 0.216 | 2.81 (2.08, 3.80) | 2.68 (2.05, 3.51) | 0.556 | 3.35 (2.46, 4.55) | 2.29 (1.67, 3.13) | 2.71 (1.64, 4.48) | 0.182 |
| Gram-negative infections | 3.83 (3.01, 4.88) | 2.65 (2.12, 3.31) | 0.030 | 3.70 (2.68, 5.09) | 2.94 (2.43, 3.56) | 0.274 | 3.46 (2.76, 4.33) | 2.78 (2.19, 3.54) | 0.105 | 3.11 (2.38, 4.06) | 3.26 (2.57, 4.13) | 2.75 (1.76, 4.28) | 0.656 |
| **Viral infections** | 2.45 (1.90, 3.16) | 2.29 (1.78, 2.96) | 0.672 | 2.28 (1.60, 3.26) | 2.41 (1.95, 2.96) | 0.873 | 2.00 (1.53, 2.62) | 2.77 (2.17, 3.53) | 0.124 | 2.75 (2.09, 3.61) | 1.93 (1.44, 2.59) | 2.78 (1.83, 4.22) | 0.183 |
| **By type of viral infection** |  |  |  |  |  |  |  |  |  |  |  |  |  |
| Acute viral infections | 2.64 (2.00, 3.49) | 2.38 (1.81, 3.13) | 0.583 | 2.52 (1.70, 3.73) | 2.50 (1.99, 3.13) | 0.913 | 2.03 (1.50, 2.74) | 2.98 (2.30, 3.86) | 0.093 | 3.16 (2.38, 4.21) | 1.86 (1.34, 2.58) | 2.84 (1.78, 4.53) | 0.049 |
| Herpesvirus (persistent) infections | 4.53 (2.71, 7.56) | 1.91 (0.99, 3.69) | 0.039 | 2.49 (1.03, 6.03) | 3.19 (2.02, 5.03) | 0.591 | 2.94 (1.66, 5.20) | 3.10 (1.75, 5.48) | 0.957 | 1.54 (0.64, 3.72) | 3.86 (2.27, 6.57) | 4.46 (1.83, 10.86) | 0.140 |
| Other persistent viral infections | 0.54 (0.17, 1.67) | 1.92 (0.72, 5.14) | 0.123 | 1.40 (0.52, 3.76) | 0.63 (0.20, 1.94) | 0.254 | 1.23 (0.51, 2.98) | 0.57 (0.14, 2.28) | 0.332 | 1.28 (0.41, 3.99) | 0.34 (0.05, 2.41) | 1.33 (0.42, 4.18) | 0.373 |
| **Parasitic infections** | 2.63 (1.09, 6.36) | 2.12 (0.68, 6.59) | 0.691 | 2.57 (0.64, 10.33) | 2.38 (1.06, 5.32) | 0.875 | 5.05 (2.39, 10.66) | 0.51 (0.07, 3.63) | 0.008 | 3.33 (1.38, 8.07) | 1.55 (0.39, 6.21) | 1.91 (0.27, 13.64) | 0.649 |
| **Fungal infections** | 2.89 (1.97, 4.26) | 2.11 (1.48, 3.01) | 0.232 | 2.38 (1.42, 4.00) | 2.43 (1.79, 3.29) | 0.931 | 2.87 (2.04, 4.04) | 1.96 (1.30, 2.95) | 0.106 | 2.43 (1.57, 3.76) | 2.22 (1.49, 3.33) | 2.91 (1.65, 5.13) | 0.823 |
| **By type of Fungal infections** |  |  |  |  |  |  |  |  |  |  |  |  |  |
| Superficial fungal infections | 2.68 (1.58, 4.56) | 1.97 (1.20, 3.24) | 0.411 | 1.99 (0.89, 4.47) | 2.35 (1.57, 3.53) | 0.715 | 2.71 (1.71, 4.28) | 1.76 (0.97, 3.21) | 0.193 | 2.12 (1.13, 3.97) | 2.01 (1.13, 3.57) | 3.20 (1.57, 6.52) | 0.682 |
| Deep fungal infections | 3.20 (1.92, 5.35) | 2.11 (1.30, 3.42) | 0.235 | 2.70 (1.44, 5.08) | 2.42 (1.58, 3.70) | 0.806 | 3.17 (2.01, 5.01) | 1.92 (1.11, 3.33) | 0.129 | 2.69 (1.52, 4.77) | 2.33 (1.37, 3.97) | 2.61 (1.15, 5.91) | 0.914 |

CI, confidence interval; HR, hazard ratio.

Based on the fully-adjusted model, adjusted for age, sex, ethnic background, education level, TDI, BMI, smoking status, alcohol consumption, physical activity.

**Table S28**. Subgroup analysis for the associations between infection types and CD Montreal phenotypes.

| **Exposure** | Non-stricturing, non-penetrating (B1) | | Stricturing (B2) | | P-heterogeneity | Penetrating (B3) | | P-heterogeneity | Perianal disease modifier (P) | | P-heterogeneity |
| --- | --- | --- | --- | --- | --- | --- | --- | --- | --- | --- | --- |
|  | HR (95%CI) | P | HR (95%CI) | P |  | HR (95%CI) | P |  | HR (95%CI) | P |  |
| All infectious | 4.60 (3.90, 5.42) | <0.001 | 10.69 (6.15, 18.57) | <0.001 | 0.004 | 17.45 (3.97, 76.74) | <0.001 | 0.079 | 26.70 (8.15, 87.48) | <0.001 | 0.004 |
| Bacterial infections | 4.75 (4.04, 5.59) | <0.001 | 10.19 (6.00, 17.31) | <0.001 | 0.007 | 12.11 (3.46, 42.33) | <0.001 | 0.146 | 21.57 (7.59, 61.31) | <0.001 | 0.005 |
| Viral infections | 2.16 (1.64, 2.84) | <0.001 | 3.11 (1.64, 5.89) | <0.001 | 0.304 | 2.08 (0.48, 9.08) | 0.331 | 0.961 | 2.78 (1.08, 7.16) | 0.035 | 0.616 |
| Parasitic infections | 6.25 (3.23, 12.11) | <0.001 | / | 0.993 | / | / | 0.997 | / | / | 0.998 | / |
| Fungal infections | 3.45 (2.48, 4.78) | <0.001 | 4.89 (2.34, 10.22) | <0.001 | 0.397 | 7.52 (2.14, 26.36) | 0.002 | 0.239 | 8.19 (3.37, 19.89) | <0.001 | 0.073 |

CI, confidence interval; HR, hazard ratio.

Based on the fully-adjusted model, adjusted for age, sex, ethnic background, education level, TDI, BMI, smoking status, alcohol consumption, physical activity.

P-heterogeneity values were calculated using Wald Z-tests comparing log-transformed HRs and standard errors between each phenotype and the B1 reference group.

**Table S29**. Associations between infection subtypes and incident CD according to disease location.

| **Exposure** | Ileal CD (L1) | | Colonic CD (L2) | | Ileocolonic or unspecified CD | | P- heterogeneity | | |
| --- | --- | --- | --- | --- | --- | --- | --- | --- | --- |
|  | HR (95% CI) | P | HR (95% CI) | P | HR (95% CI) | P | L2 vs L1 | Ileocolonic/unspecified vs L1 | Ileocolonic/unspecified vs L2 |
| All infectious | 3.90 (2.70, 5.63) | <0.001 | 4.59 (2.89, 7.28) | <0.001 | 5.53 (4.59, 6.66) | <0.001 | 0.589 | 0.097 | 0.463 |
| Bacterial infections | 4.34 (3.01, 6.27) | <0.001 | 4.86 (3.07, 7.70) | <0.001 | 5.50 (4.58, 6.60) | <0.001 | 0.706 | 0.257 | 0.624 |
| Viral infections | 1.84 (0.93, 3.64) | 0.08 | 2.83 (1.40, 5.70) | 0.004 | 2.34 (1.76, 3.13) | <0.001 | 0.389 | 0.525 | 0.623 |
| Parasitic infections | 4.10 (0.57, 29.39) | 0.161 | 11.11 (2.71, 45.65) | 0.001 | 4.87 (2.17, 10.91) | <0.001 | 0.420 | 0.874 | 0.320 |
| Fungal infections | 1.43 (0.45, 4.52) | 0.543 | 5.46 (2.49, 12.00) | <0.001 | 3.79 (2.71, 5.32) | <0.001 | 0.060 | 0.112 | 0.403 |

CI, confidence interval; HR, hazard ratio; TDI, Townsend deprivation index; BMI, body mass index; CD, Crohn’s disease.

Based on the fully adjusted model, adjusted for age, sex, ethnic background, education level, TDI, BMI, smoking status, alcohol consumption, and physical activity.

P- heterogeneity were calculated using Wald Z-tests comparing log-transformed HRs and standard errors between each pair of disease-location groups. L1 denotes ileal CD, and L2 denotes colonic CD.

**Sensitivity analyses**

**Table S30.** Sensitivity analysis of the associations between infection and incident IBD, CD, and UC using a complete-case approach.

| **Exposure** | **IBD** | | | **CD** | | | **UC** | | |
| --- | --- | --- | --- | --- | --- | --- | --- | --- | --- |
|  | **Case/person-year** | **HR (95%CI)** | ***P*** | **Case/person-year** | **HR (95%CI)** | ***P*** | **Case/person-year** | **HR (95%CI)** | ***P*** |
| **All infectious** | 840/2,995,702 | Ref |  | 242/2,991,442 | Ref |  | 626/2,994,159 | Ref |  |
|  | 1127/1,205,644 | 3.46 (3.15, 3.80) | <0.001 | 498/1,202,883 | 5.13 (4.38, 6.01) | <0.001 | 807/1,204,280 | 3.36 (3.01, 3.74) | <0.001 |
| **Bacterial infections** | 877/3,084,935 | Ref |  | 256/3,080,535 | Ref |  | 655/3,083,336 | Ref |  |
|  | 1090/1,116,411 | 3.55 (3.23, 3.89) | <0.001 | 484/1,113,791 | 5.23 (4.47, 6.11) | <0.001 | 778/1,115,102 | 3.42 (3.07, 3.81) | <0.001 |
| **By severity** |  |  |  |  |  |  |  |  |  |
| Invasive bacterial infections | 1417/3,622,294 | Ref |  | 502/3,616,745 | Ref |  | 1030/3,620,033 | Ref |  |
|  | 550/579,052 | 2.34 (2.12, 2.59) | <0.001 | 238/577,581 | 2.75 (2.34, 3.22) | <0.001 | 403/578,406 | 2.39 (2.12, 2.69) | <0.001 |
| Localized bacterial infections | 1025/3,368,442 | Ref |  | 314/3,363,581 | Ref |  | 765/3,366,636 | Ref |  |
|  | 942/832,904 | 3.78 (3.45, 4.14) | <0.001 | 426/830,744 | 5.39 (4.63, 6.27) | <0.001 | 668/831,802 | 3.62 (3.25, 4.04) | <0.001 |
| Bacterial infections with sepsis | 1817/4,109,331 | Ref |  | 673/4,102,647 | Ref |  | 1332/4,106,568 | Ref |  |
|  | 150/92,015 | 2.98 (2.51, 3.53) | <0.001 | 67/91,679 | 3.43 (2.65, 4.44) | <0.001 | 101/91,871 | 2.74 (2.23, 3.37) | <0.001 |
| Bacterial infections without sepsis | 883/3,095,586 | Ref |  | 260/3,091,182 | Ref |  | 657/3,093,977 | Ref |  |
|  | 1084/1,105,760 | 3.55 (3.24, 3.90) | <0.001 | 480/1,103,143 | 5.18 (4.43, 6.05) | <0.001 | 776/1,104,462 | 3.45 (3.10, 3.84) | <0.001 |
| **By location** |  |  |  |  |  |  |  |  |  |
| Extracellular bacterial infections | 1713/4,003,177 | Ref |  | 633/3,996,784 | Ref |  | 1242/4,000,539 | Ref |  |
|  | 254/198,169 | 2.66 (2.32, 3.05) | <0.001 | 107/197,541 | 2.88 (2.33, 3.55) | <0.001 | 191/197,900 | 2.79 (2.39, 3.26) | <0.001 |
| Intracellular bacterial infections | 1929/4,181,456 | Ref |  | 720/4,174,499 | Ref |  | 1405/4,178,574 | Ref |  |
|  | 38/19,890 | 4.16 (3.02, 5.75) | <0.001 | 20/19,826 | 5.85 (3.74, 9.13) | <0.001 | 28/19,864 | 4.21 (2.89, 6.13) | <0.001 |
| **By Gram stain** |  |  |  |  |  |  |  |  |  |
| Gram-positive infections | 1843/4,100,709 | Ref |  | 692/4,094,038 | Ref |  | 1334/4,097,904 | Ref |  |
|  | 124/100,637 | 2.47 (2.06, 2.97) | <0.001 | 48/100,287 | 2.43 (1.80, 3.27) | <0.001 | 99/100,535 | 2.76 (2.24, 3.39) | <0.001 |
| Gram-negative infections | 1752/4,060,245 | Ref |  | 640/4,053,683 | Ref |  | 1275/4,057,555 | Ref |  |
|  | 215/141,101 | 3.08 (2.66, 3.56) | <0.001 | 100/140,642 | 3.73 (3.00, 4.63) | <0.001 | 158/140,884 | 3.14 (2.65, 3.72) | <0.001 |
| **Viral infections** | 1801/4,039,891 | Ref |  | 673/4,033,432 | Ref |  | 1307/4,037,184 | Ref |  |
|  | 166/161,455 | 2.28 (1.94, 2.68) | <0.001 | 67/160,894 | 2.32 (1.80, 3.00) | <0.001 | 126/161,255 | 2.42 (2.01, 2.91) | <0.001 |
| **By type of viral infection** |  |  |  |  |  |  |  |  |  |
| Acute viral infections | 1832/4,077,656 | Ref |  | 691/4,071,110 | Ref |  | 1327/4,074,894 | Ref |  |
|  | 135/123,690 | 2.34 (1.96, 2.79) | <0.001 | 49/123,215 | 2.12 (1.58, 2.84) | <0.001 | 106/123,545 | 2.57 (2.11, 3.14) | <0.001 |
| Herpesvirus (persistent) infections | 1937/4,179,644 | Ref |  | 723/4,172,698 | Ref |  | 1411/4,176,782 | Ref |  |
|  | 30/21,702 | 2.91 (2.02, 4.17) | <0.001 | 17/21,628 | 4.22 (2.60, 6.83) | <0.001 | 22/21,657 | 2.96 (1.94, 4.52) | <0.001 |
| Other persistent viral infections | 1957/4,177,352 | Ref |  | 734/4,170,353 | Ref |  | 1427/4,174,455 | Ref |  |
|  | 10/23,994 | 1.02 (0.55, 1.90) | 0.955 | 6/23,972 | 1.52 (0.68, 3.41) | 0.3058 | 6/23,983 | 0.85 (0.38, 1.91) | 0.6995 |
| **Parasitic infections** | 1955/4,191,837 | Ref |  | 731/4,184,828 | Ref |  | 1426/4,188,934 | Ref |  |
|  | 12/9,509 | 2.87 (1.62, 5.07) | 0 | 9/9,497 | 5.85 (3.03, 11.32) | <0.001 | 7/9,505 | 2.28 (1.08, 4.80) | 0.0298 |
| **Fungal infections** | 1880/4,141,018 | Ref |  | 693/4,134,190 | Ref |  | 1376/4,138,249 | Ref |  |
|  | 87/60,328 | 2.76 (2.22, 3.42) | <0.001 | 47/60,135 | 3.80 (2.82, 5.12) | <0.001 | 57/60,190 | 2.50 (1.92, 3.27) | <0.001 |
| **By type of Fungal infections** |  |  |  |  |  |  |  |  |  |
| Superficial fungal infections | 1923/4,168,818 | Ref |  | 716/4,161,891 | Ref |  | 1405/4,165,969 | Ref |  |
|  | 44/32,528 | 2.54 (1.88, 3.43) | <0.001 | 24/32,435 | 3.43 (2.28, 5.17) | <0.001 | 28/32,470 | 2.26 (1.55, 3.29) | <0.001 |
| Deep fungal infections | 1920/4,170,769 | Ref |  | 715/4,163,852 | Ref |  | 1402/4,167,948 | Ref |  |
|  | 47/30,577 | 2.87 (2.14, 3.84) | <0.001 | 25/30,473 | 3.91 (2.62, 5.84) | <0.001 | 31/30,490 | 2.61 (1.82, 3.73) | <0.001 |

CI, confidence interval; HR, hazard ratio; IBD, inflammatory bowel disease; CD, Crohn's disease; UC, ulcerative colitis; CCI, Charlson Comorbidity Index; INFLA score, low-grade inflammation score.

Based on the fully-adjusted model, adjusted for age, sex, ethnic background, education level, TDI, BMI, smoking status, alcohol consumption, physical activity, CCI or INFLA score.

**Table S31.** Sensitivity analyses of the associations between infection and incident IBD, CD, and UC after further adjustment for CCI, INFLA score, and baseline use of proton pump inhibitors and nonsteroidal anti-inflammatory drugs.

| **Exposure** | **IBD HR (95%CI)** | | | **CD HR (95%CI)** | | | **UC HR (95%CI)** | | |
| --- | --- | --- | --- | --- | --- | --- | --- | --- | --- |
|  | **Further adjusted for CCI** | **Further adjusted for INFLA score** | **Further adjusted for PPIs and NSAIDS** | **Further adjusted for CCI** | **Further adjusted for INFLA score** | **Further adjusted for PPIs and NSAIDS** | **Further adjusted for CCI** | **Further adjusted for INFLA score** | **Further adjusted for PPIs and NSAIDS** |
| **All infectious** | 2.94 (2.67, 3.23) | 3.36 (3.06, 3.70) | 3.36 (3.06, 3.68) | 4.42 (3.76, 5.20) | 5.08 (4.31, 5.99) | 4.92 (4.20, 5.75) | 2.84 (2.54, 3.17) | 3.22 (2.89, 3.60) | 3.27 (2.94, 3.64) |
| **Bacterial infections** | 3.02 (2.75, 3.32) | 3.44 (3.13, 3.78) | 3.45 (3.15, 3.78) | 4.53 (3.86, 5.31) | 5.14 (4.37, 6.05) | 5.03 (4.31, 5.87) | 2.90 (2.60, 3.24) | 3.28 (2.93, 3.66) | 3.34 (3.01, 3.72) |
| **By severity** |  |  |  |  |  |  |  |  |  |
| Invasive bacterial infections | 1.78 (1.59, 1.98) | 2.32 (2.09, 2.58) | 2.27 (2.05, 2.51) | 2.13 (1.80, 2.52) | 2.86 (2.43, 3.37) | 2.66 (2.27, 3.11) | 1.80 (1.58, 2.04) | 2.32 (2.06, 2.63) | 2.31 (2.06, 2.60) |
| Localized bacterial infections | 3.21 (2.92, 3.53) | 3.67 (3.34, 4.03) | 3.68 (3.36, 4.03) | 4.60 (3.94, 5.37) | 5.17 (4.43, 6.05) | 5.12 (4.41, 5.95) | 3.09 (2.76, 3.45) | 3.52 (3.15, 3.94) | 3.57 (3.22, 3.97) |
| Bacterial infections with sepsis | 1.72 (1.44, 2.05) | 2.93 (2.47, 3.48) | 2.87 (2.44, 3.39) | 1.96 (1.50, 2.57) | 3.47 (2.68, 4.48) | 3.24 (2.52, 4.17) | 1.58 (1.28, 1.95) | 2.68 (2.17, 3.31) | 2.70 (2.21, 3.29) |
| Bacterial infections without sepsis | 3.04 (2.76, 3.34) | 3.44 (3.13, 3.78) | 3.46 (3.16, 3.79) | 4.49 (3.83, 5.26) | 5.08 (4.32, 5.97) | 4.98 (4.27, 5.82) | 2.93 (2.63, 3.28) | 3.30 (2.96, 3.69) | 3.38 (3.04, 3.75) |
| **By location** |  |  |  |  |  |  |  |  |  |
| Extracellular bacterial infections | 1.79 (1.55, 2.06) | 2.61 (2.28, 2.99) | 2.49 (2.18, 2.84) | 1.90 (1.53, 2.37) | 2.83 (2.29, 3.50) | 2.63 (2.14, 3.24) | 1.89 (1.61, 2.22) | 2.74 (2.34, 3.22) | 2.64 (2.27, 3.08) |
| Intracellular bacterial infections | 2.97 (2.16, 4.07) | 3.81 (2.74, 5.31) | 3.79 (2.77, 5.20) | 4.39 (2.86, 6.74) | 5.53 (3.54, 8.65) | 5.52 (3.60, 8.47) | 2.82 (1.93, 4.12) | 3.68 (2.48, 5.48) | 3.65 (2.51, 5.32) |
| **By Gram stain** |  |  |  |  |  |  |  |  |  |
| Gram-positive infections | 1.54 (1.28, 1.86) | 2.38 (1.98, 2.87) | 2.28 (1.91, 2.73) | 1.42 (1.04, 1.93) | 2.36 (1.75, 3.17) | 2.11 (1.57, 2.84) | 1.75 (1.42, 2.15) | 2.68 (2.17, 3.30) | 2.59 (2.11, 3.17) |
| Gram-negative infections | 2.07 (1.78, 2.40) | 3.02 (2.61, 3.50) | 2.89 (2.51, 3.33) | 2.53 (2.02, 3.17) | 3.62 (2.91, 4.50) | 3.46 (2.79, 4.28) | 2.10 (1.77, 2.51) | 3.09 (2.60, 3.67) | 2.96 (2.51, 3.50) |
| **Viral infections** | 1.64 (1.39, 1.93) | 2.22 (1.88, 2.62) | 2.13 (1.82, 2.49) | 1.66 (1.29, 2.15) | 2.31 (1.79, 2.99) | 2.16 (1.68, 2.77) | 1.73 (1.44, 2.09) | 2.35 (1.94, 2.84) | 2.26 (1.89, 2.71) |
| **By type of viral infection** |  |  |  |  |  |  |  |  |  |
| Acute viral infections | 1.64 (1.38, 1.96) | 2.24 (1.86, 2.68) | 2.15 (1.81, 2.56) | 1.49 (1.11, 1.99) | 2.03 (1.50, 2.73) | 1.95 (1.46, 2.60) | 1.81 (1.48, 2.21) | 2.47 (2.01, 3.04) | 2.37 (1.95, 2.89) |
| Herpesvirus (persistent) infections | 2.02 (1.42, 2.87) | 2.99 (2.08, 4.29) | 2.76 (1.95, 3.92) | 2.75 (1.70, 4.48) | 4.30 (2.65, 6.97) | 3.76 (2.32, 6.09) | 2.09 (1.39, 3.14) | 3.07 (2.01, 4.68) | 2.88 (1.92, 4.31) |
| Other persistent viral infections | 0.86 (0.49, 1.52) | 1.09 (0.58, 2.03) | 1.10 (0.62, 1.94) | 1.26 (0.60, 2.66) | 1.90 (0.90, 4.00) | 1.60 (0.76, 3.37) | 0.69 (0.33, 1.46) | 0.76 (0.32, 1.84) | 0.90 (0.43, 1.89) |
| **Parasitic infections** | 2.43 (1.41, 4.20) | 2.53 (1.36, 4.72) | 2.79 (1.61, 4.82) | 4.53 (2.34, 8.75) | 4.74 (2.25, 10.00) | 5.21 (2.69, 10.07) | 2.03 (1.01, 4.07) | 2.09 (0.93, 4.66) | 2.34 (1.17, 4.69) |
| **Fungal infections** | 1.64 (1.31, 2.04) | 2.50 (1.99, 3.13) | 2.45 (1.98, 3.04) | 2.28 (1.68, 3.10) | 3.51 (2.58, 4.77) | 3.35 (2.49, 4.51) | 1.49 (1.13, 1.94) | 2.24 (1.69, 2.96) | 2.26 (1.74, 2.94) |
| **By location** |  |  |  |  |  |  |  |  |  |
| Superficial fungal infections | 1.45 (1.07, 1.95) | 2.38 (1.75, 3.25) | 2.35 (1.75, 3.15) | 1.95 (1.29, 2.95) | 3.40 (2.25, 5.12) | 3.14 (2.10, 4.69) | 1.31 (0.90, 1.89) | 2.06 (1.38, 3.06) | 2.15 (1.49, 3.09) |
| Deep fungal infections | 1.76 (1.32, 2.35) | 2.46 (1.81, 3.35) | 2.50 (1.87, 3.34) | 2.35 (1.57, 3.54) | 3.36 (2.21, 5.10) | 3.29 (2.20, 4.93) | 1.60 (1.12, 2.28) | 2.22 (1.51, 3.26) | 2.31 (1.62, 3.28) |

CI, confidence interval; HR, hazard ratio; IBD, inflammatory bowel disease; CD, Crohn's disease; UC, ulcerative colitis; CCI, Charlson Comorbidity Index; INFLA score, low-grade inflammation score; PPIs, proton pump inhibitors; NSAIDs, nonsteroidal anti-inflammatory drugs.

Based on the fully-adjusted model, adjusted for age, sex, ethnic background, education level, TDI, BMI, smoking status, alcohol consumption, physical activity, CCI or INFLA score.

**Table S32**. Sensitivity analyses of the associations between infection and incident IBD, CD, and UC with further adjustment for baseline antibiotic use.

| **Exposure** | **IBD** | | | **CD** | | | **UC** | | |
| --- | --- | --- | --- | --- | --- | --- | --- | --- | --- |
|  | **Case/person-year** | **HR (95%CI)** | ***P*** | **Case/person-year** | **HR (95%CI)** | ***P*** | **Case/person-year** | **HR (95%CI)** | ***P*** |
| **All infectious** | 881/3,103,147 | Ref |  | 252/3,098,594 | Ref |  | 659/3,101,536 | Ref |  |
|  | 1187/1,260,774 | 3.43 (3.13, 3.75) | <0.001 | 519/1,257,816 | 5.06 (4.33, 5.91) | <0.001 | 852/1,259,369 | 3.34 (3.00, 3.71) | <0.001 |
| **Bacterial infections** | 918/3,196,023 | Ref |  | 266/3,191,329 | Ref |  | 688/3,194,356 | Ref |  |
|  | 1150/1,167,898 | 3.53 (3.22, 3.86) | <0.001 | 505/1,165,080 | 5.17 (4.44, 6.04) | <0.001 | 823/1,166,548 | 3.42 (3.08, 3.80) | <0.001 |
| **By severity** |  |  |  |  |  |  |  |  |  |
| Invasive bacterial infections | 1482/3,755,688 | Ref |  | 518/3,749,766 | Ref |  | 1082/3,753,348 | Ref |  |
|  | 586/608,233 | 2.35 (2.13, 2.59) | <0.001 | 253/606,643 | 2.78 (2.38, 3.25) | <0.001 | 429/607,557 | 2.39 (2.13, 2.68) | <0.001 |
| Localized bacterial infections | 1072/3,490,197 | Ref |  | 328/3,485,039 | Ref |  | 800/3,488,314 | Ref |  |
|  | 996/873,724 | 3.76 (3.44, 4.12) | <0.001 | 443/871,370 | 5.28 (4.55, 6.12) | <0.001 | 711/872,590 | 3.65 (3.29, 4.06) | <0.001 |
| Bacterial infections with sepsis | 1907/4,266,272 | Ref |  | 700/4,259,144 | Ref |  | 1401/4,263,402 | Ref |  |
|  | 161/97,650 | 2.98 (2.53, 3.51) | <0.001 | 71/97,266 | 3.41 (2.65, 4.38) | <0.001 | 110/97,502 | 2.80 (2.30, 3.42) | <0.001 |
| Bacterial infections without sepsis | 924/3,207,050 | Ref |  | 270/3,202,352 | Ref |  | 690/3,205,371 | Ref |  |
|  | 1144/1,156,872 | 3.54 (3.23, 3.87) | <0.001 | 501/1,154,057 | 5.13 (4.40, 5.98) | <0.001 | 821/1,155,533 | 3.45 (3.10, 3.83) | <0.001 |
| **By location** |  |  |  |  |  |  |  |  |  |
| Extracellular bacterial infections | 1801/4,153,610 | Ref |  | 660/4,146,766 | Ref |  | 1309/4,150,864 | Ref |  |
|  | 267/210,311 | 2.58 (2.26, 2.95) | <0.001 | 111/209,644 | 2.77 (2.25, 3.41) | <0.001 | 202/210,040 | 2.74 (2.36, 3.19) | <0.001 |
| Intracellular bacterial infections | 2028/4,342,896 | Ref |  | 749/4,335,448 | Ref |  | 1483/4,339,910 | Ref |  |
|  | 40/21,025 | 4.06 (2.97, 5.56) | <0.001 | 22/20,961 | 6.01 (3.93, 9.21) | <0.001 | 28/20,995 | 3.90 (2.68, 5.67) | <0.001 |
| **By Gram stain** |  |  |  |  |  |  |  |  |  |
| Gram-positive infections | 1938/4,256,622 | Ref |  | 723/4,249,492 | Ref |  | 1406/4,253,707 | Ref |  |
|  | 130/107,300 | 2.38 (1.99, 2.85) | <0.001 | 48/106,918 | 2.23 (1.66, 3.00) | <0.001 | 105/107,197 | 2.70 (2.21, 3.30) | <0.001 |
| Gram-negative infections | 1841/4,214,083 | Ref |  | 666/4,207,062 | Ref |  | 1344/4,211,287 | Ref |  |
|  | 227/149,838 | 3.01 (2.61, 3.46) | <0.001 | 105/149,347 | 3.64 (2.95, 4.51) | <0.001 | 167/149,617 | 3.09 (2.62, 3.64) | <0.001 |
| **Viral infections** | 1894/4,193,830 | Ref |  | 701/4,186,916 | Ref |  | 1379/4,191,021 | Ref |  |
|  | 174/170,091 | 2.22 (1.90, 2.60) | <0.001 | 70/169,493 | 2.28 (1.77, 2.92) | <0.001 | 132/169,883 | 2.36 (1.97, 2.83) | <0.001 |
| **By type of viral infection** |  |  |  |  |  |  |  |  |  |
| Acute viral infections | 1928/4,233,714 | Ref |  | 720/4,226,697 | Ref |  | 1401/4,230,850 | Ref |  |
|  | 140/130,207 | 2.25 (1.89, 2.68) | <0.001 | 51/129,713 | 2.07 (1.55, 2.75) | <0.001 | 110/130,054 | 2.48 (2.04, 3.02) | <0.001 |
| Herpesvirus (persistent) infections | 2036/4,340,897 | Ref |  | 754/4,333,472 | Ref |  | 1487/4,337,925 | Ref |  |
|  | 32/23,025 | 2.85 (2.01, 4.04) | <0.001 | 17/22,937 | 3.91 (2.41, 6.33) | <0.001 | 24/22,979 | 2.98 (1.99, 4.46) | <0.001 |
| Other persistent viral infections | 2056/4,338,562 | Ref |  | 764/4,331,082 | Ref |  | 1504/4,335,556 | Ref |  |
|  | 12/25,359 | 1.13 (0.64, 2.00) | 0.666 | 7/25,327 | 1.65 (0.78, 3.49) | 0.186 | 7/25,348 | 0.93 (0.44, 1.95) | 0.837 |
| **Parasitic infections** | 2055/4,353,919 | Ref |  | 762/4,346,422 | Ref |  | 1503/4,350,906 | Ref |  |
|  | 13/10,002 | 2.83 (1.64, 4.89) | <0.001 | 9/9,987 | 5.31 (2.75, 10.27) | <0.001 | 8/9,999 | 2.38 (1.19, 4.78) | 0.014 |
| **Fungal infections** | 1979/4,299,651 | Ref |  | 723/4,292,340 | Ref |  | 1452/4,296,772 | Ref |  |
|  | 89/64,270 | 2.58 (2.08, 3.20) | <0.001 | 48/64,069 | 3.57 (2.65, 4.80) | <0.001 | 59/64,132 | 2.39 (1.84, 3.10) | <0.001 |
| **By type of Fungal infections** |  |  |  |  |  |  |  |  |  |
| Superficial fungal infections | 2020/4,331,172 | Ref |  | 746/4,323,772 | Ref |  | 1479/4,328,242 | Ref |  |
|  | 48/32,749 | 2.67 (2.00, 3.56) | <0.001 | 25/32,637 | 3.58 (2.39, 5.34) | <0.001 | 32/32,662 | 2.48 (1.74, 3.52) | <0.001 |
| Deep fungal infections | 2022/4,329,336 | Ref |  | 746/4,321,926 | Ref |  | 1481/4,326,378 | Ref |  |
|  | 46/34,585 | 2.44 (1.82, 3.27) | <0.001 | 25/34,483 | 3.30 (2.21, 4.93) | <0.001 | 30/34,527 | 2.24 (1.56, 3.22) | <0.001 |

CI, confidence interval; HR, hazard ratio; IBD, inflammatory bowel disease; CD, Crohn's disease; UC, ulcerative colitis.

Based on the fully-adjusted model, adjusted for age, sex, ethnic background, education level, TDI, BMI, smoking status, alcohol consumption, physical activity, baseline antibiotic use.

**Table S33**. Sensitivity analysis of the associations between infectious diseases and risk of IBD, CD, and UC after excluding participants with post-baseline infections (N = 270,749).

| **Exposure** | **IBD** | | | **CD** | | | **UC** | | |
| --- | --- | --- | --- | --- | --- | --- | --- | --- | --- |
|  | **Case/person-year** | **HR (95%CI)** | ***P*** | **Case/person-year** | **HR (95%CI)** | ***P*** | **Case/person-year** | **HR (95%CI)** | ***P*** |
| **All infectious** | 881/3,103,147 | Ref |  | 252/3,098,594 | Ref |  | 659/3,098,594 | Ref |  |
|  | 433/527,697 | 2.63 (2.34, 2.96) | <0.001 | 186/526,074 | 3.78 (3.11, 4.59) | <0.001 | 326/526,074 | 13.18 (8.58, 20.25) | <0.001 |
| **Bacterial infections** | 916/3,155,877 | Ref |  | 269/3,151,169 | Ref |  | 683/3,151,169 | Ref |  |
|  | 398/474,967 | 2.62 (2.33, 2.96) | <0.001 | 169/473,499 | 3.62 (2.97, 4.42) | <0.001 | 302/473,499 | 11.41 (7.57, 17.18) | <0.001 |
| **By severity** |  |  |  |  |  |  |  |  |  |
| Invasive bacterial infections | 1138/3,417,635 | Ref |  | 362/3,412,085 | Ref |  | 851/3,412,085 | Ref |  |
|  | 176/213,209 | 2.20 (1.87, 2.58) | <0.001 | 76/212,583 | 2.85 (2.22, 3.66) | <0.001 | 134/212,583 | 5.78 (3.81, 8.75) | <0.001 |
| Localized bacterial infections | 1042/3,318,468 | Ref |  | 320/3,313,313 | Ref |  | 778/3,313,313 | Ref |  |
|  | 272/312,375 | 2.51 (2.19, 2.88) | <0.001 | 118/311,355 | 3.35 (2.70, 4.16) | <0.001 | 207/311,355 | 8.28 (5.62, 12.19) | <0.001 |
| Bacterial infections with sepsis | 1307/3,619,354 | Ref |  | 436/3,613,200 | Ref |  | 980/3,613,200 | Ref |  |
|  | 7/11,490 | 1.43 (0.68, 3.02) | 0.342 | 2/11,467 | 1.19 (0.30, 4.80) | 0.802 | 5/11,467 | / | / |
| Bacterial infections without sepsis | 920/3,160,414 | Ref |  | 271/3,155,695 | Ref |  | 685/3,155,695 | Ref |  |
|  | 394/470,430 | 2.61 (2.32, 2.95) | <0.001 | 167/468,972 | 3.59 (2.94, 4.38) | <0.001 | 300/468,972 | 11.54 (7.66, 17.38) | <0.001 |
| **By location** |  |  |  |  |  |  |  |  |  |
| Extracellular bacterial infections | 1266/3,581,956 | Ref |  | 419/3,575,958 | Ref |  | 947/3,575,958 | Ref |  |
|  | 48/48,888 | 2.44 (1.83, 3.26) | <0.001 | 19/48,710 | 2.75 (1.73, 4.38) | <0.001 | 38/48,710 | 4.98 (2.50, 9.93) | <0.001 |
| Intracellular bacterial infections | 1305/3,623,777 | Ref |  | 434/3,617,619 | Ref |  | 978/3,617,619 | Ref |  |
|  | 9/7,067 | 3.13 (1.62, 6.04) | 0.001 | 4/7,049 | 4.09 (1.53, 10.97) | 0.005 | 7/7,049 | 7.86 (1.93, 31.98) | 0.004 |
| **By Gram stain** |  |  |  |  |  |  |  |  |  |
| Gram-positive infections | 1280/3,597,231 | Ref |  | 426/3,591,186 | Ref |  | 957/3,591,186 | Ref |  |
|  | 34/33,613 | 2.46 (1.75, 3.46) | <0.001 | 12/33,481 | 2.47 (1.39, 4.40) | 0.002 | 28/33,481 | 4.59 (2.00, 10.53) | <0.001 |
| Gram-negative infections | 1287/3,605,841 | Ref |  | 425/3,599,748 | Ref |  | 964/3,599,748 | Ref |  |
|  | 27/25,002 | 2.70 (1.84, 3.96) | <0.001 | 13/24,920 | 3.70 (2.12, 6.44) | <0.001 | 21/24,920 | 7.69 (3.55, 16.66) | <0.001 |
| **Viral infections** | 1280/3,578,465 | Ref |  | 423/3,572,456 | Ref |  | 960/3,572,456 | Ref |  |
|  | 34/52,379 | 1.66 (1.18, 2.33) | 0.004 | 15/52,212 | 2.03 (1.21, 3.41) | 0.007 | 25/52,212 | 3.08 (1.35, 7.06) | 0.008 |
| **By type of viral infection** |  |  |  |  |  |  |  |  |  |
| Acute viral infections | 1291/3,595,022 | Ref |  | 427/3,588,955 | Ref |  | 969/3,588,955 | Ref |  |
|  | 23/35,822 | 1.65 (1.09, 2.49) | 0.018 | 11/35,712 | 2.23 (1.22, 4.06) | 0.009 | 16/35,712 | 3.03 (1.11, 8.27) | 0.030 |
| Herpesvirus (persistent) infections | 1308/3,624,350 | Ref |  | 435/3,618,201 | Ref |  | 980/3,618,201 | Ref |  |
|  | 6/6,494 | 2.31 (1.03, 5.15) | 0.041 | 3/6,466 | 3.23 (1.04, 10.07) | 0.043 | 5/6,466 | 8.09 (1.99, 32.87) | 0.003 |
| Other persistent viral infections | 1308/3,619,182 | Ref |  | 436/3,613,035 | Ref |  | 980/3,613,035 | Ref |  |
|  | 6/11,662 | 1.25 (0.56, 2.80) | 0.582 | 2/11,632 | 1.08 (0.27, 4.36) | 0.909 | 5/11,632 | 1.99 (0.28, 14.34) | 0.496 |
| **Parasitic infections** | 1314/3,627,480 | Ref |  | 438/3,621,304 | Ref |  | 985/3,621,304 | Ref |  |
|  | 0/3,364 | / | / | 0/3,364 | 0.00 (0.00, Inf) | 0.984 | 0/3,364 | / | / |
| **Fungal infections** | 1301/3,619,498 | Ref |  | 431/3,613,362 | Ref |  | 977/3,613,362 | Ref |  |
|  | 13/11,345 | 2.82 (1.63, 4.87) | <0.001 | 7/11,306 | 4.10 (1.94, 8.67) | <0.001 | 8/11,306 | 4.29 (1.05, 17.47) | 0.042 |
| **By type of Fungal infections** |  |  |  |  |  |  |  |  |  |
| Superficial fungal infections | 1304/3,624,656 | Ref |  | 434/3,618,520 | Ref |  | 107/3,616,042 | Ref |  |
|  | 10/6,188 | 4.11 (2.20, 7.66) | <0.001 | 4/6,148 | 4.30 (1.60, 11.53) | 0.004 | 2/6,127 | 8.04 (1.97, 32.78) | 0.004 |
| Deep fungal infections | 1311/3,625,553 | Ref |  | 435/3,619,377 | Ref |  | 109/3,616,897 | Ref |  |
|  | 3/5,291 | 1.34 (0.43, 4.15) | 0.617 | 3/5,291 | 3.71 (1.19, 11.58) | 0.024 | 0/5,272 | / | / |

CI, confidence interval; HR, hazard ratio; IBD, inflammatory bowel disease; CD, Crohn's disease; UC, ulcerative colitis.

Based on the fully-adjusted model, adjusted for age, sex, ethnic background, education level, TDI, BMI, smoking status, alcohol consumption, physical activity.

**Table S34.** Sensitivity analysis of the associations between infectious diseases and risk of IBD, CD, and UC after including both hospital-treated and self-reported infections (N = 498,107).

| **Exposure** | **IBD** | | | **CD** | | | **UC** | | |
| --- | --- | --- | --- | --- | --- | --- | --- | --- | --- |
|  | **Case/person-year** | **HR (95%CI)** | ***P*** | **Case/person-year** | **HR (95%CI)** | ***P*** | **Case/person-year** | **HR (95%CI)** | ***P*** |
| **All infectious** | 1625/4,772,118 | Ref |  | 599/4,765,049 | Ref |  | 1255/4,769,483 | Ref |  |
|  | 1274/1,334,015 | 2.85 (2.64, 3.08) | <0.001 | 555/1,330,827 | 3.27 (2.90, 3.69) | <0.001 | 910/1,332,434 | 2.65 (2.42, 2.89) | <0.001 |
| **Bacterial infections** | 1664/4,869,642 | Ref |  | 615/4,862,432 | Ref |  | 1284/4,866,942 | Ref |  |
|  | 1235/1,236,491 | 2.96 (2.74, 3.20) | <0.001 | 539/1,233,444 | 3.40 (3.01, 3.83) | <0.001 | 881/1,234,975 | 2.74 (2.51, 3.00) | <0.001 |
| **By severity** |  |  |  |  |  |  |  |  |  |
| Invasive bacterial infections | 2237/5,445,271 | Ref |  | 869/5,436,814 | Ref |  | 1685/5,441,891 | Ref |  |
|  | 662/660,862 | 2.34 (2.14, 2.56) | <0.001 | 285/659,062 | 2.52 (2.20, 2.90) | <0.001 | 480/660,027 | 2.26 (2.04, 2.51) | <0.001 |
| Localized bacterial infections | 1828/5,178,393 | Ref |  | 681/5,170,696 | Ref |  | 1402/5,175,458 | Ref |  |
|  | 1071/927,740 | 3.27 (3.02, 3.53) | <0.001 | 473/925,180 | 3.76 (3.33, 4.25) | <0.001 | 763/926,460 | 3.05 (2.78, 3.34) | <0.001 |
| Bacterial infections with sepsis | 2701/5,990,678 | Ref |  | 1066/5,980,873 | Ref |  | 2031/5,986,683 | Ref |  |
|  | 198/115,455 | 3.07 (2.65, 3.56) | <0.001 | 88/115,003 | 3.37 (2.69, 4.21) | <0.001 | 134/115,234 | 2.77 (2.32, 3.31) | <0.001 |
| Bacterial infections without sepsis | 1670/4,881,147 | Ref |  | 619/4,873,934 | Ref |  | 1286/4,878,437 | Ref |  |
|  | 1229/1,224,986 | 2.97 (2.75, 3.21) | <0.001 | 535/1,221,942 | 3.40 (3.01, 3.83) | <0.001 | 879/1,223,481 | 2.77 (2.53, 3.03) | <0.001 |
| **By location** |  |  |  |  |  |  |  |  |  |
| Extracellular bacterial infections | 2585/5,872,484 | Ref |  | 1022/5,863,003 | Ref |  | 1932/5,868,629 | Ref |  |
|  | 314/233,649 | 2.69 (2.39, 3.04) | <0.001 | 132/232,873 | 2.76 (2.29, 3.33) | <0.001 | 233/233,288 | 2.70 (2.35, 3.10) | <0.001 |
| Intracellular bacterial infections | 2855/6,081,735 | Ref |  | 1130/6,071,545 | Ref |  | 2135/6,077,565 | Ref |  |
|  | 44/24,398 | 3.80 (2.82, 5.12) | <0.001 | 24/24,331 | 5.25 (3.50, 7.89) | <0.001 | 30/24,353 | 3.46 (2.41, 4.97) | <0.001 |
| **By Gram stain** |  |  |  |  |  |  |  |  |  |
| Gram-positive infections | 2746/5,985,689 | Ref |  | 1097/5,975,859 | Ref |  | 2044/5,981,614 | Ref |  |
|  | 153/120,444 | 2.46 (2.09, 2.90) | <0.001 | 57/120,017 | 2.22 (1.70, 2.91) | <0.001 | 121/120,304 | 2.65 (2.20, 3.19) | <0.001 |
| Gram-negative infections | 2632/5,939,133 | Ref |  | 1031/5,929,462 | Ref |  | 1970/5,935,197 | Ref |  |
|  | 267/167,000 | 3.13 (2.75, 3.56) | <0.001 | 123/166,414 | 3.56 (2.94, 4.31) | <0.001 | 195/166,721 | 3.08 (2.65, 3.58) | <0.001 |
| **Viral infections** | 2697/5,916,415 | Ref |  | 1069/5,906,828 | Ref |  | 2018/5,912,483 | Ref |  |
|  | 202/189,718 | 2.28 (1.97, 2.63) | <0.001 | 85/189,048 | 2.32 (1.86, 2.91) | <0.001 | 147/189,435 | 2.25 (1.90, 2.66) | <0.001 |
| **By type of vural infection** |  |  |  |  |  |  |  |  |  |
| Acute viral infections | 2736/5,962,593 | Ref |  | 1091/5,952,891 | Ref |  | 2043/5,958,594 | Ref |  |
|  | 163/143,540 | 2.34 (2.00, 2.75) | <0.001 | 63/142,984 | 2.18 (1.69, 2.82) | <0.001 | 122/143,323 | 2.38 (1.98, 2.87) | <0.001 |
| Herpesvirus (persistent) infections | 2860/6,080,126 | Ref |  | 1133/6,069,968 | Ref |  | 2136/6,075,969 | Ref |  |
|  | 39/26,007 | 3.07 (2.23, 4.21) | <0.001 | 21/25,908 | 4.06 (2.63, 6.25) | <0.001 | 29/25,949 | 3.08 (2.14, 4.45) | <0.001 |
| Other persistent viral infections | 2885/6,075,166 | Ref |  | 1147/6,064,957 | Ref |  | 2156/6,070,961 | Ref |  |
|  | 14/30,967 | 1.06 (0.62, 1.79) | 0.842 | 7/30,919 | 1.27 (0.60, 2.67) | 0.533 | 9/30,956 | 0.93 (0.48, 1.80) | 0.835 |
| **Parasitic infections** | 2884/6,094,647 | Ref |  | 1145/6,084,409 | Ref |  | 2155/6,090,436 | Ref |  |
|  | 15/11,486 | 2.87 (1.73, 4.77) | <0.001 | 9/11,467 | 4.35 (2.25, 8.40) | <0.001 | 10/11,482 | 2.57 (1.38, 4.78) | 0.003 |
| **Fungal infections** | 2790/6,033,715 | Ref |  | 1100/6,023,706 | Ref |  | 2091/6,029,647 | Ref |  |
|  | 109/72,418 | 2.81 (2.31, 3.40) | <0.001 | 54/72,169 | 3.38 (2.56, 4.45) | <0.001 | 74/72,271 | 2.58 (2.04, 3.25) | <0.001 |
| **By type of Fungal infections** |  |  |  |  |  |  |  |  |  |
| Superficial fungal infections | 2839/6,067,153 | Ref |  | 1125/6,057,033 | Ref |  | 2124/6,063,003 | Ref |  |
|  | 60/38,980 | 2.83 (2.19, 3.67) | <0.001 | 29/38,843 | 3.27 (2.25, 4.73) | <0.001 | 41/38,915 | 2.64 (1.94, 3.60) | <0.001 |
| Deep fungal infections | 2845/6,068,881 | Ref |  | 1127/6,058,750 | Ref |  | 2129/6,064,756 | Ref |  |
|  | 54/37,252 | 2.63 (2.01, 3.45) | <0.001 | 27/37,126 | 3.22 (2.19, 4.72) | <0.001 | 36/37,162 | 2.36 (1.70, 3.29) | <0.001 |

CI, confidence interval; HR, hazard ratio; IBD, inflammatory bowel disease; CD, Crohn's disease; UC, ulcerative colitis.

Based on the fully-adjusted model, adjusted for age, sex, ethnic background, education level, TDI, BMI, smoking status, alcohol consumption, physical activity.

**Table S35**. Sensitivity analyses of the associations between infection and incident IBD, CD, and UC after exclusion of individuals diagnosed with both CD and UC.

| **Exposure** | **IBD** | | | **CD** | | | **UC** | | |
| --- | --- | --- | --- | --- | --- | --- | --- | --- | --- |
|  | **Case/person-year** | **HR (95%CI)** | ***P*** | **Case/person-year** | **HR (95%CI)** | ***P*** | **Case/person-year** | **HR (95%CI)** | ***P*** |
| **All infectious** | 851/3,102,978 | Ref |  | 222/3,098,383 | Ref |  | 629/3,101,326 | Ref |  |
|  | 1003/1,260,270 | 3.07 (2.79, 3.37) | <0.001 | 335/1,257,181 | 3.81 (3.20, 4.54) | <0.001 | 668/1,258,778 | 2.81 (2.51, 3.14) | <0.001 |
| **Bacterial infections** | 882/3,195,834 | Ref |  | 230/3,191,094 | Ref |  | 652/3,194,127 | Ref |  |
|  | 972/1,167,413 | 3.17 (2.89, 3.49) | <0.001 | 327/1,164,469 | 3.98 (3.34, 4.75) | <0.001 | 645/1,165,977 | 2.89 (2.58, 3.23) | <0.001 |
| **By severity** |  |  |  |  |  |  |  |  |  |
| Invasive bacterial infections | 1364/3,755,317 | Ref |  | 400/3,749,281 | Ref |  | 964/3,752,910 | Ref |  |
|  | 490/607,930 | 2.19 (1.96, 2.43) | <0.001 | 157/606,283 | 2.31 (1.91, 2.79) | <0.001 | 333/607,194 | 2.14 (1.88, 2.43) | <0.001 |
| Localized bacterial infections | 1016/3,489,944 | Ref |  | 272/3,484,733 | Ref |  | 744/3,488,010 | Ref |  |
|  | 838/873,303 | 3.41 (3.10, 3.75) | <0.001 | 285/870,831 | 4.21 (3.54, 5.00) | <0.001 | 553/872,094 | 3.12 (2.79, 3.50) | <0.001 |
| Bacterial infections with sepsis | 1713/4,265,665 | Ref |  | 506/4,258,371 | Ref |  | 1207/4,262,687 | Ref |  |
|  | 141/97,583 | 3.03 (2.54, 3.61) | <0.001 | 51/97,192 | 3.62 (2.70, 4.87) | <0.001 | 90/97,417 | 2.78 (2.24, 3.46) | <0.001 |
| Bacterial infections without sepsis | 888/3,206,860 | Ref |  | 234/3,202,117 | Ref |  | 654/3,205,142 | Ref |  |
|  | 966/1,156,387 | 3.18 (2.89, 3.49) | <0.001 | 323/1,153,446 | 3.92 (3.29, 4.66) | <0.001 | 643/1,154,962 | 2.91 (2.60, 3.26) | <0.001 |
| **By location** |  |  |  |  |  |  |  |  |  |
| Extracellular bacterial infections | 1633/4,153,080 | Ref |  | 492/4,146,094 | Ref |  | 1141/4,150,248 | Ref |  |
|  | 221/210,167 | 2.44 (2.11, 2.82) | <0.001 | 65/209,470 | 2.28 (1.75, 2.97) | <0.001 | 156/209,857 | 2.52 (2.12, 2.99) | <0.001 |
| Intracellular bacterial infections | 1824/4,342,237 | Ref |  | 545/4,334,642 | Ref |  | 1279/4,339,126 | Ref |  |
|  | 30/21,011 | 3.44 (2.39, 4.94) | <0.001 | 12/20,922 | 4.63 (2.61, 8.21) | <0.001 | 18/20,979 | 2.95 (1.85, 4.70) | <0.001 |
| **By Gram stain** |  |  |  |  |  |  |  |  |  |
| Gram-positive infections | 1747/4,256,022 | Ref |  | 532/4,248,738 | Ref |  | 1215/4,253,011 | Ref |  |
|  | 107/107,225 | 2.24 (1.84, 2.73) | <0.001 | 25/106,826 | 1.65 (1.10, 2.47) | 0.016 | 82/107,093 | 2.52 (2.01, 3.16) | <0.001 |
| Gram-negative infections | 1672/4,213,531 | Ref |  | 497/4,206,376 | Ref |  | 1175/4,210,640 | Ref |  |
|  | 182/149,716 | 2.75 (2.35, 3.21) | <0.001 | 60/149,187 | 2.93 (2.23, 3.85) | <0.001 | 122/149,464 | 2.67 (2.21, 3.24) | <0.001 |
| **Viral infections** | 1708/4,193,233 | Ref |  | 515/4,186,158 | Ref |  | 1193/4,190,304 | Ref |  |
|  | 146/170,015 | 2.12 (1.79, 2.52) | <0.001 | 42/169,406 | 1.93 (1.40, 2.64) | <0.001 | 104/169,801 | 2.22 (1.81, 2.71) | <0.001 |
| **By type of viral infection** |  |  |  |  |  |  |  |  |  |
| Acute viral infections | 1735/4,233,096 | Ref |  | 527/4,225,916 | Ref |  | 1208/4,230,112 | Ref |  |
|  | 119/130,152 | 2.19 (1.82, 2.64) | <0.001 | 30/129,648 | 1.73 (1.19, 2.50) | 0.004 | 89/129,992 | 2.41 (1.94, 2.99) | <0.001 |
| Herpesvirus (persistent) infections | 1831/4,340,252 | Ref |  | 549/4,332,659 | Ref |  | 1282/4,337,154 | Ref |  |
|  | 23/22,996 | 2.35 (1.56, 3.55) | <0.001 | 8/22,904 | 2.64 (1.31, 5.32) | 0.006 | 15/22,950 | 2.23 (1.34, 3.72) | 0.002 |
| Other persistent viral infections | 1844/4,337,892 | Ref |  | 552/4,330,240 | Ref |  | 1292/4,334,760 | Ref |  |
|  | 10/25,356 | 1.07 (0.57, 1.99) | 0.832 | 5/25,324 | 1.66 (0.69, 4.03) | 0.259 | 5/25,344 | 0.79 (0.33, 1.90) | 0.596 |
| **Parasitic infections** | 1845/4,353,258 | Ref |  | 552/4,345,589 | Ref |  | 1293/4,350,121 | Ref |  |
|  | 9/9,990 | 2.22 (1.15, 4.28) | 0.017 | 5/9,974 | 4.16 (1.72, 10.07) | 0.002 | 4/9,983 | 1.40 (0.52, 3.74) | 0.503 |
| **Fungal infections** | 1783/4,299,033 | Ref |  | 527/4,291,561 | Ref |  | 1256/4,296,033 | Ref |  |
|  | 71/64,214 | 2.39 (1.88, 3.03) | <0.001 | 30/64,003 | 3.25 (2.24, 4.71) | <0.001 | 41/64,071 | 2.01 (1.47, 2.75) | <0.001 |
| **By type of Fungal infections** |  |  |  |  |  |  |  |  |  |
| Superficial fungal infections | 1817/4,328,693 | Ref |  | 541/4,321,117 | Ref |  | 1276/4,325,613 | Ref |  |
|  | 37/34,555 | 2.28 (1.65, 3.17) | <0.001 | 16/34,447 | 3.08 (1.87, 5.09) | <0.001 | 21/34,492 | 1.91 (1.24, 2.95) | 0.003 |
| Deep fungal infections | 1815/4,330,524 | Ref |  | 541/4,322,957 | Ref |  | 1274/4,327,468 | Ref |  |
|  | 39/32,723 | 2.53 (1.84, 3.48) | <0.001 | 16/32,607 | 3.38 (2.05, 5.57) | <0.001 | 23/32,636 | 2.16 (1.43, 3.27) | <0.001 |

CI, confidence interval; HR, hazard ratio; IBD, inflammatory bowel disease; CD, Crohn's disease; UC, ulcerative colitis.

Based on the fully-adjusted model, adjusted for age, sex, ethnic background, education level, TDI, BMI, smoking status, alcohol consumption, physical activity.

**Table S36.** Sensitivity analyses of the associations between infection and incident IBD, CD, and UC restricting the outcome definition to participants with at least two IBD diagnoses.

| **Exposure** | **IBD** | | | **CD** | | | **UC** | | |
| --- | --- | --- | --- | --- | --- | --- | --- | --- | --- |
|  | **Case/person-year** | **HR (95%CI)** | ***P*** | **Case/person-year** | **HR (95%CI)** | ***P*** | **Case/person-year** | **HR (95%CI)** | ***P*** |
| **All infectious** | 43/3,108,564 | Ref |  | 15/3,108,420 | Ref |  | 32/3,108,513 | Ref |  |
|  | 139/1,267,472 | 7.41 (5.23, 10.52) | <0.001 | 72/1,267,305 | 10.73 (6.09, 18.91) | <0.001 | 112/1,267,410 | 8.17 (5.47, 12.20) | <0.001 |
| **Bacterial infections** | 45/3,201,611 | Ref |  | 16/3,201,463 | Ref |  | 34/3,201,560 | Ref |  |
|  | 137/1,174,425 | 7.81 (5.53, 11.03) | <0.001 | 71/1,174,262 | 11.08 (6.37, 19.26) | <0.001 | 110/1,174,363 | 8.45 (5.70, 12.51) | <0.001 |
| **By severity** |  |  |  |  |  |  |  |  |  |
| Invasive bacterial infections | 110/3,764,100 | Ref |  | 50/3,763,874 | Ref |  | 85/3,764,015 | Ref |  |
|  | 72/611,936 | 3.66 (2.69, 4.97) | <0.001 | 37/611,851 | 3.98 (2.56, 6.19) | <0.001 | 59/611,908 | 3.95 (2.80, 5.57) | <0.001 |
| Localized bacterial infections | 67/3,496,823 | Ref |  | 24/3,496,636 | Ref |  | 54/3,496,775 | Ref |  |
|  | 115/879,213 | 6.32 (4.64, 8.61) | <0.001 | 63/879,089 | 9.39 (5.80, 15.20) | <0.001 | 90/879,148 | 6.24 (4.41, 8.82) | <0.001 |
| Bacterial infections with sepsis | 168/4,277,476 | Ref |  | 78/4,277,173 | Ref |  | 133/4,277,363 | Ref |  |
|  | 14/98,560 | 2.59 (1.49, 4.51) | 0.001 | 9/98,552 | 3.40 (1.68, 6.90) | 0.001 | 11/98,560 | 2.65 (1.42, 4.96) | 0.002 |
| Bacterial infections without sepsis | 45/3,212,669 | Ref |  | 16/3,212,521 | Ref |  | 34/3,212,618 | Ref |  |
|  | 137/1,163,367 | 7.95 (5.63, 11.22) | <0.001 | 71/1,163,204 | 11.27 (6.49, 19.60) | <0.001 | 110/1,163,305 | 8.59 (5.80, 12.73) | <0.001 |
| **By location** |  |  |  |  |  |  |  |  |  |
| Extracellular bacterial infections | 146/4,164,066 | Ref |  | 64/4,163,774 | Ref |  | 119/4,163,994 | Ref |  |
|  | 36/211,970 | 3.98 (2.74, 5.79) | <0.001 | 23/211,951 | 5.54 (3.38, 9.08) | <0.001 | 25/211,929 | 3.46 (2.22, 5.38) | <0.001 |
| Intracellular bacterial infections | 174/4,354,688 | Ref |  | 82/4,354,391 | Ref |  | 140/4,354,590 | Ref |  |
|  | 8/21,348 | 9.19 (4.50, 18.74) | <0.001 | 5/21,334 | 11.50 (4.63, 28.57) | <0.001 | 4/21,333 | 5.85 (2.16, 15.87) | 0.001 |
| **By Gram stain** |  |  |  |  |  |  |  |  |  |
| Gram-positive infections | 170/4,267,826 | Ref |  | 80/4,267,522 | Ref |  | 135/4,267,732 | Ref |  |
|  | 12/108,211 | 2.37 (1.31, 4.29) | 0.004 | 7/108,203 | 2.77 (1.27, 6.07) | 0.011 | 9/108,191 | 2.28 (1.15, 4.52) | 0.018 |
| Gram-negative infections | 150/4,224,812 | Ref |  | 66/4,224,525 | Ref |  | 124/4,224,740 | Ref |  |
|  | 32/151,225 | 4.76 (3.22, 7.06) | <0.001 | 21/151,200 | 6.76 (4.06, 11.24) | <0.001 | 20/151,183 | 3.66 (2.26, 5.94) | <0.001 |
| **Viral infections** | 162/4,204,954 | Ref |  | 75/4,204,684 | Ref |  | 129/4,204,860 | Ref |  |
|  | 20/171,083 | 2.76 (1.72, 4.40) | <0.001 | 12/171,041 | 3.42 (1.85, 6.34) | <0.001 | 15/171,063 | 2.63 (1.53, 4.50) | <0.001 |
| **By type of viral infection** |  |  |  |  |  |  |  |  |  |
| Acute viral infections | 166/4,245,062 | Ref |  | 79/4,244,794 | Ref |  | 132/4,244,961 | Ref |  |
|  | 16/130,974 | 2.75 (1.64, 4.62) | <0.001 | 8/130,931 | 2.74 (1.31, 5.71) | 0.007 | 12/130,962 | 2.64 (1.45, 4.79) | 0.001 |
| Herpesvirus (persistent) infections | 179/4,352,842 | Ref |  | 84/4,352,529 | Ref |  | 141/4,352,728 | Ref |  |
|  | 3/23,195 | 2.86 (0.91, 8.95) | 0.072 | 3/23,196 | 5.82 (1.83, 18.47) | 0.003 | 3/23,195 | 3.71 (1.18, 11.65) | 0.025 |
| Other persistent viral infections | 180/4,350,591 | Ref |  | 85/4,350,279 | Ref |  | 144/4,350,487 | Ref |  |
|  | 2/25,446 | 2.03 (0.50, 8.23) | 0.322 | 2/25,446 | 4.36 (1.06, 17.94) | 0.041 | 0/25,436 | / | 0.992 |
| **Parasitic infections** | 179/4,365,960 | Ref |  | 84/4,365,649 | Ref |  | 142/4,365,848 | Ref |  |
|  | 3/10,076 | 7.20 (2.29, 22.67) | 0.001 | 3/10,076 | 14.79 (4.62, 47.32) | <0.001 | 2/10,075 | 6.27 (1.55, 25.44) | 0.010 |
| **Fungal infections** | 172/4,311,227 | Ref |  | 80/4,310,918 | Ref |  | 137/4,311,119 | Ref |  |
|  | 10/64,809 | 3.04 (1.60, 5.78) | 0.001 | 7/64,807 | 4.33 (1.98, 9.47) | <0.001 | 7/64,804 | 2.71 (1.26, 5.82) | 0.011 |
| **By type of Fungal infections** |  |  |  |  |  |  |  |  |  |
| Superficial fungal infections | 178/4,341,126 | Ref |  | 85/4,340,821 | Ref |  | 141/4,341,013 | Ref |  |
|  | 4/34,910 | 2.20 (0.81, 5.94) | 0.121 | 2/34,904 | 2.12 (0.52, 8.68) | 0.296 | 3/34,910 | 2.11 (0.67, 6.66) | 0.202 |
| Deep fungal infections | 175/4,343,043 | Ref |  | 82/4,342,730 | Ref |  | 139/4,342,934 | Ref |  |
|  | 7/32,994 | 4.11 (1.92, 8.78) | <0.001 | 5/32,995 | 6.03 (2.43, 14.99) | <0.001 | 5/32,989 | 3.75 (1.53, 9.20) | 0.004 |

CI, confidence interval; HR, hazard ratio; IBD, inflammatory bowel disease; CD, Crohn's disease; UC, ulcerative colitis.

Based on the fully-adjusted model, adjusted for age, sex, ethnic background, education level, TDI, BMI, smoking status, alcohol consumption, physical activity.

**Table S37**. Sensitivity analyses of the associations between infection and incident IBD by time since infection.

| **Exposure** | **In one year** | | | **In 2-5 year** | | | **>5 year** | | |
| --- | --- | --- | --- | --- | --- | --- | --- | --- | --- |
|  | **Case/person-year** | **HR (95%CI)** | **P** | **Case/person-year** | **HR (95%CI)** | **P** | **Case/person-year** | **HR (95%CI)** | **P** |
| **All infectious** | 63/229,990 | Ref |  | 213/1,149,313 | Ref |  | 605/3,102,503 | Ref |  |
|  | 303/129,426 | 8.71 (6.61, 11.47) | <0.001 | 458/645,582 | 3.58 (3.03, 4.23) | <0.001 | 426/1,259,372 | 2.21 (1.94, 2.51) | <0.001 |
| **Bacterial infections** | 68/240,008 | Ref |  | 225/1,199,369 | Ref |  | 625/3,195,342 | Ref |  |
|  | 298/119,409 | 9.04 (6.91, 11.81) | <0.001 | 446/595,526 | 3.74 (3.18, 4.41) | <0.001 | 406/1,166,533 | 2.23 (1.96, 2.54) | <0.001 |
| **By severity** |  |  |  |  |  |  |  |  |  |
| Invasive bacterial infections | 247/296,902 | Ref |  | 429/1,483,067 | Ref |  | 806/3,754,404 | Ref |  |
|  | 119/62,514 | 2.15 (1.72, 2.70) | <0.001 | 242/311,828 | 2.41 (2.05, 2.83) | <0.001 | 225/607,471 | 1.99 (1.71, 2.32) | <0.001 |
| Localized bacterial infections | 93/270,532 | Ref |  | 289/1,351,787 | Ref |  | 690/3,489,320 | Ref |  |
|  | 273/88,884 | 9.22 (7.25, 11.72) | <0.001 | 382/443,108 | 3.80 (3.25, 4.44) | <0.001 | 341/872,555 | 2.42 (2.11, 2.77) | <0.001 |
| Bacterial infections with sepsis | 320/345,396 | Ref |  | 604/1,725,021 | Ref |  | 983/4,264,449 | Ref |  |
|  | 46/14,021 | 3.30 (2.41, 4.53) | <0.001 | 67/69,874 | 2.36 (1.83, 3.05) | <0.001 | 48/97,426 | 2.44 (1.81, 3.27) | <0.001 |
| Bacterial infections without sepsis | 71/242,328 | Ref |  | 227/1,210,961 | Ref |  | 626/3,206,360 | Ref |  |
|  | 295/117,088 | 8.80 (6.76, 11.46) | <0.001 | 444/583,934 | 3.81 (3.23, 4.49) | <0.001 | 405/1,155,515 | 2.24 (1.97, 2.55) | <0.001 |
| **By location** |  |  |  |  |  |  |  |  |  |
| Extracellular bacterial infections | 307/336,227 | Ref |  | 557/1,679,307 | Ref |  | 937/4,151,915 | Ref |  |
|  | 59/23,189 | 2.62 (1.97, 3.48) | <0.001 | 114/115,587 | 2.64 (2.15, 3.24) | <0.001 | 94/209,961 | 2.20 (1.77, 2.73) | <0.001 |
| Intracellular bacterial infections | 356/357,711 | Ref |  | 651/1,786,426 | Ref |  | 1021/4,340,913 | Ref |  |
|  | 10/1,705 | 5.28 (2.81, 9.92) | <0.001 | 20/8,469 | 5.80 (3.71, 9.06) | <0.001 | 10/20,963 | 2.19 (1.17, 4.08) | 0.014 |
| **By Gram stain** |  |  |  |  |  |  |  |  |  |
| Gram-positive infections | 339/348,830 | Ref |  | 616/1,742,142 | Ref |  | 983/4,254,745 | Ref |  |
|  | 27/10,586 | 2.38 (1.60, 3.54) | <0.001 | 55/52,753 | 2.59 (1.96, 3.42) | <0.001 | 48/107,130 | 2.01 (1.50, 2.69) | <0.001 |
| Gram-negative infections | 312/341,947 | Ref |  | 573/1,707,857 | Ref |  | 956/4,212,340 | Ref |  |
|  | 54/17,469 | 3.22 (2.40, 4.32) | <0.001 | 98/87,038 | 2.96 (2.38, 3.69) | <0.001 | 75/149,535 | 2.51 (1.97, 3.18) | <0.001 |
| **Viral infections** | 333/343,378 | Ref |  | 607/1,714,891 | Ref |  | 954/4,191,983 | Ref |  |
|  | 33/16,038 | 1.97 (1.38, 2.82) | <0.001 | 64/80,003 | 2.06 (1.59, 2.68) | <0.001 | 77/169,892 | 2.28 (1.80, 2.88) | <0.001 |
| **By type of viral infection** |  |  |  |  |  |  |  |  |  |
| Acute viral infections | 341/346,593 | Ref |  | 619/1,730,926 | Ref |  | 968/4,231,830 | Ref |  |
|  | 25/12,823 | 1.83 (1.22, 2.76) | 0.004 | 52/63,969 | 2.07 (1.56, 2.75) | <0.001 | 63/130,045 | 2.41 (1.86, 3.11) | <0.001 |
| Herpesvirus (persistent) infections | 359/357,164 | Ref |  | 660/1,783,674 | Ref |  | 1017/4,338,885 | Ref |  |
|  | 7/2,252 | 2.98 (1.41, 6.30) | 0.004 | 11/11,221 | 2.42 (1.33, 4.40) | 0.004 | 14/22,991 | 2.96 (1.75, 5.03) | <0.001 |
| Other persistent viral infections | 362/357,834 | Ref |  | 666/1,787,001 | Ref |  | 1028/4,336,531 | Ref |  |
|  | 4/1,582 | 2.20 (0.82, 5.93) | 0.118 | 5/7,894 | 1.59 (0.66, 3.83) | 0.305 | 3/25,344 | 0.54 (0.17, 1.67) | 0.285 |
| **Parasitic infections** | 361/358,589 | Ref |  | 665/1,790,782 | Ref |  | 1029/4,351,894 | Ref |  |
|  | 5/828 | 5.78 (2.38, 14.01) | <0.001 | 6/4,113 | 3.80 (1.70, 8.50) | 0.001 | 2/9,982 | 0.93 (0.23, 3.72) | 0.917 |
| **Fungal infections** | 343/351,698 | Ref |  | 638/1,756,407 | Ref |  | 998/4,297,722 | Ref |  |
|  | 23/7,718 | 2.85 (1.86, 4.35) | <0.001 | 33/38,488 | 2.05 (1.44, 2.92) | <0.001 | 33/64,153 | 2.43 (1.71, 3.44) | <0.001 |
| **By type of Fungal infections** |  |  |  |  |  |  |  |  |  |
| Superficial fungal infections | 351/355,016 | Ref |  | 660/1,772,946 | Ref |  | 1011/4,327,331 | Ref |  |
|  | 15/4,401 | 3.22 (1.91, 5.41) | <0.001 | 11/21,949 | 1.16 (0.64, 2.12) | 0.618 | 20/34,544 | 2.72 (1.74, 4.24) | <0.001 |
| Deep fungal infections | 357/355,774 | Ref |  | 647/1,776,742 | Ref |  | 1016/4,329,207 | Ref |  |
|  | 9/3,642 | 2.26 (1.16, 4.39) | 0.016 | 24/18,153 | 3.15 (2.09, 4.74) | <0.001 | 15/32,668 | 2.11 (1.27, 3.52) | 0.004 |

CI, confidence interval; HR, hazard ratio; IBD, inflammatory bowel disease.

Based on the fully-adjusted model, adjusted for age, sex, ethnic background, education level, TDI, BMI, smoking status, alcohol consumption, physical activity.

**Table S38.** Sensitivity analyses of the associations between infection and incident CD by time since infection.

| **Exposure** | **In one year** | | | **In 2-5 year** | | | **>5 year** | | |
| --- | --- | --- | --- | --- | --- | --- | --- | --- | --- |
|  | **Case/person-year** | **HR (95%CI)** | **P** | **Case/person-year** | **HR (95%CI)** | **P** | **Case/person-year** | **HR (95%CI)** | **P** |
| **All infectious** | 16/229,380 | Ref |  | 63/1,146,714 | Ref |  | 173/3,098,417 | Ref |  |
|  | 143/128,856 | 16.00 (9.50, 26.97) | <0.001 | 195/643,628 | 5.07 (3.79, 6.78) | <0.001 | 181/1,257,204 | 3.19 (2.57, 3.97) | <0.001 |
| **Bacterial infections** | 19/239,376 | Ref |  | 66/1,196,686 | Ref |  | 181/3,191,139 | Ref |  |
|  | 140/118,860 | 14.99 (9.23, 24.35) | <0.001 | 192/593,656 | 5.43 (4.08, 7.23) | <0.001 | 173/1,164,482 | 3.20 (2.58, 3.97) | <0.001 |
| **By severity** |  |  |  |  |  |  |  |  |  |
| Invasive bacterial infections | 106/296,018 | Ref |  | 149/1,479,571 | Ref |  | 263/3,749,323 | Ref |  |
|  | 53/62,218 | 2.17 (1.54, 3.05) | <0.001 | 109/310,771 | 3.11 (2.41, 4.01) | <0.001 | 91/606,298 | 2.38 (1.86, 3.04) | <0.001 |
| Localized bacterial infections | 25/269,817 | Ref |  | 98/1,348,816 | Ref |  | 205/3,484,746 | Ref |  |
|  | 134/88,419 | 16.73 (10.85, 25.79) | <0.001 | 160/441,526 | 4.59 (3.55, 5.94) | <0.001 | 149/870,875 | 3.45 (2.77, 4.30) | <0.001 |
| Bacterial infections with sepsis | 136/344,294 | Ref |  | 227/1,720,734 | Ref |  | 337/4,258,457 | Ref |  |
|  | 23/13,942 | 3.80 (2.42, 5.98) | <0.001 | 31/69,608 | 2.94 (2.01, 4.32) | <0.001 | 17/97,164 | 2.39 (1.46, 3.91) | 0.001 |
| Bacterial infections without sepsis | 21/241,695 | Ref |  | 67/1,208,275 | Ref |  | 182/3,202,157 | Ref |  |
|  | 138/116,541 | 13.73 (8.62, 21.86) | <0.001 | 191/582,067 | 5.48 (4.12, 7.28) | <0.001 | 172/1,153,464 | 3.19 (2.57, 3.97) | <0.001 |
| **By location** |  |  |  |  |  |  |  |  |  |
| Extracellular bacterial infections | 136/335,183 | Ref |  | 213/1,675,205 | Ref |  | 311/4,146,117 | Ref |  |
|  | 23/23,053 | 2.23 (1.42, 3.50) | <0.001 | 45/115,138 | 2.65 (1.91, 3.69) | <0.001 | 43/209,504 | 2.89 (2.09, 4.01) | <0.001 |
| Intracellular bacterial infections | 153/356,545 | Ref |  | 247/1,781,927 | Ref |  | 349/4,334,693 | Ref |  |
|  | 6/1,691 | 7.54 (3.32, 17.10) | <0.001 | 11/8,415 | 8.47 (4.62, 15.54) | <0.001 | 5/20,928 | 3.16 (1.30, 7.66) | 0.011 |
| **By Gram stain** |  |  |  |  |  |  |  |  |  |
| Gram-positive infections | 153/347,721 | Ref |  | 234/1,737,832 | Ref |  | 336/4,248,771 | Ref |  |
|  | 6/10,515 | 1.13 (0.50, 2.57) | 0.770 | 24/52,510 | 2.92 (1.91, 4.47) | <0.001 | 18/106,850 | 2.09 (1.29, 3.38) | 0.003 |
| Gram-negative infections | 133/340,872 | Ref |  | 215/1,703,653 | Ref |  | 318/4,206,413 | Ref |  |
|  | 26/17,364 | 3.55 (2.31, 5.46) | <0.001 | 43/86,689 | 3.40 (2.44, 4.76) | <0.001 | 36/149,208 | 3.44 (2.42, 4.90) | <0.001 |
| **Viral infections** | 142/342,290 | Ref |  | 231/1,710,698 | Ref |  | 328/4,186,219 | Ref |  |
|  | 17/15,946 | 2.37 (1.43, 3.93) | 0.001 | 27/79,645 | 2.20 (1.47, 3.28) | <0.001 | 26/169,402 | 2.10 (1.40, 3.15) | <0.001 |
| **By type of viral infection** |  |  |  |  |  |  |  |  |  |
| Acute viral infections | 149/345,491 | Ref |  | 238/1,726,672 | Ref |  | 333/4,225,978 | Ref |  |
|  | 10/12,745 | 1.66 (0.87, 3.16) | 0.123 | 20/63,670 | 1.99 (1.26, 3.15) | 0.003 | 21/129,643 | 2.20 (1.41, 3.44) | 0.001 |
| Herpesvirus (persistent) infections | 155/355,997 | Ref |  | 251/1,779,175 | Ref |  | 348/4,332,703 | Ref |  |
|  | 4/2,239 | 3.87 (1.43, 10.46) | 0.008 | 7/11,168 | 4.01 (1.89, 8.51) | <0.001 | 6/22,918 | 3.53 (1.57, 7.92) | 0.002 |
| Other persistent viral infections | 155/356,659 | Ref |  | 256/1,782,467 | Ref |  | 353/4,330,301 | Ref |  |
|  | 4/1,577 | 5.40 (1.99, 14.70) | 0.001 | 2/7,876 | 1.46 (0.36, 5.91) | 0.593 | 1/25,321 | 0.48 (0.07, 3.40) | 0.460 |
| **Parasitic infections** | 155/357,412 | Ref |  | 254/1,786,241 | Ref |  | 353/4,345,647 | Ref |  |
|  | 4/824 | 11.57 (4.27, 31.33) | <0.001 | 4/4,102 | 6.59 (2.44, 17.75) | <0.001 | 1/9,974 | 1.34 (0.19, 9.58) | 0.769 |
| **Fungal infections** | 146/350,553 | Ref |  | 239/1,751,987 | Ref |  | 338/4,291,617 | Ref |  |
|  | 13/7,683 | 3.68 (2.07, 6.52) | <0.001 | 19/38,355 | 3.06 (1.91, 4.90) | <0.001 | 16/64,004 | 3.24 (1.95, 5.38) | <0.001 |
| **By type of Fungal infections** |  |  |  |  |  |  |  |  |  |
| Superficial fungal infections | 149/353,854 | Ref |  | 253/1,768,465 | Ref |  | 344/4,321,154 | Ref |  |
|  | 10/4,382 | 4.89 (2.56, 9.33) | <0.001 | 5/21,877 | 1.32 (0.54, 3.20) | 0.542 | 10/34,467 | 3.66 (1.94, 6.90) | <0.001 |
| Deep fungal infections | 155/354,614 | Ref |  | 244/1,772,259 | Ref |  | 347/4,323,033 | Ref |  |
|  | 4/3,622 | 2.27 (0.84, 6.15) | 0.106 | 14/18,083 | 4.79 (2.78, 8.23) | <0.001 | 7/32,588 | 2.74 (1.29, 5.80) | 0.009 |

CI, confidence interval; HR, hazard ratio; CD, Crohn's disease.

Based on the fully-adjusted model, adjusted for age, sex, ethnic background, education level, TDI, BMI, smoking status, alcohol consumption, physical activity.

**Table S39**. Sensitivity analyses of the associations between infection and incident UC by time since infection.

| **Exposure** | **In one year** | | | **In 2-5 year** | | | **>5 year** | | |
| --- | --- | --- | --- | --- | --- | --- | --- | --- | --- |
|  | **Case/person-year** | **HR (95%CI)** | **P** | **Case/person-year** | **HR (95%CI)** | **P** | **Case/person-year** | **HR (95%CI)** | **P** |
| **All infectious** | 47/229,776 | Ref |  | 156/1,148,417 | Ref |  | 456/3,101,051 | Ref |  |
|  | 207/129,152 | 8.03 (5.82, 11.08) | <0.001 | 335/644,646 | 3.61 (2.97, 4.38) | <0.001 | 310/1,258,358 | 2.15 (1.85, 2.49) | <0.001 |
| **Bacterial infections** | 51/239,786 | Ref |  | 167/1,198,438 | Ref |  | 470/3,193,839 | Ref |  |
|  | 203/119,142 | 8.26 (6.04, 11.29) | <0.001 | 324/594,625 | 3.69 (3.05, 4.47) | <0.001 | 296/1,165,570 | 2.18 (1.87, 2.53) | <0.001 |
| **By severity** |  |  |  |  |  |  |  |  |  |
| Invasive bacterial infections | 169/296,550 | Ref |  | 321/1,481,707 | Ref |  | 592/3,752,383 | Ref |  |
|  | 85/62,378 | 2.27 (1.74, 2.97) | <0.001 | 170/311,355 | 2.26 (1.87, 2.74) | <0.001 | 174/607,025 | 2.12 (1.78, 2.52) | <0.001 |
| Localized bacterial infections | 73/270,271 | Ref |  | 209/1,350,701 | Ref |  | 518/3,487,671 | Ref |  |
|  | 181/88,658 | 7.82 (5.93, 10.32) | <0.001 | 282/442,361 | 3.92 (3.26, 4.71) | <0.001 | 248/871,737 | 2.36 (2.02, 2.76) | <0.001 |
| Bacterial infections with sepsis | 227/344,946 | Ref |  | 448/1,723,303 | Ref |  | 726/4,262,049 | Ref |  |
|  | 27/13,982 | 2.74 (1.82, 4.11) | <0.001 | 43/69,760 | 2.03 (1.48, 2.79) | <0.001 | 40/97,359 | 2.77 (2.00, 3.82) | <0.001 |
| Bacterial infections without sepsis | 52/242,104 | Ref |  | 168/1,210,020 | Ref |  | 470/3,204,851 | Ref |  |
|  | 202/116,825 | 8.29 (6.08, 11.30) | <0.001 | 323/583,042 | 3.77 (3.11, 4.57) | <0.001 | 296/1,154,557 | 2.20 (1.89, 2.56) | <0.001 |
| **By location** |  |  |  |  |  |  |  |  |  |
| Extracellular bacterial infections | 211/335,794 | Ref |  | 404/1,677,661 | Ref |  | 694/4,149,637 | Ref |  |
|  | 43/23,135 | 2.81 (2.01, 3.93) | <0.001 | 87/115,401 | 2.79 (2.21, 3.54) | <0.001 | 72/209,771 | 2.30 (1.79, 2.94) | <0.001 |
| Intracellular bacterial infections | 248/357,233 | Ref |  | 479/1,784,618 | Ref |  | 756/4,338,450 | Ref |  |
|  | 6/1,695 | 4.58 (2.04, 10.33) | <0.001 | 12/8,444 | 4.70 (2.65, 8.36) | <0.001 | 10/20,959 | 2.95 (1.58, 5.51) | 0.001 |
| **By Gram stain** |  |  |  |  |  |  |  |  |  |
| Gram-positive infections | 232/348,364 | Ref |  | 449/1,740,381 | Ref |  | 725/4,252,343 | Ref |  |
|  | 22/10,565 | 2.87 (1.84, 4.47) | <0.001 | 42/52,681 | 2.73 (1.98, 3.75) | <0.001 | 41/107,065 | 2.35 (1.71, 3.24) | <0.001 |
| Gram-negative infections | 216/341,509 | Ref |  | 421/1,706,189 | Ref |  | 707/4,210,004 | Ref |  |
|  | 38/17,419 | 3.31 (2.33, 4.71) | <0.001 | 70/86,874 | 2.89 (2.24, 3.74) | <0.001 | 59/149,405 | 2.69 (2.06, 3.53) | <0.001 |
| **Viral infections** | 230/342,927 | Ref |  | 442/1,713,199 | Ref |  | 707/4,189,671 | Ref |  |
|  | 24/16,001 | 2.09 (1.37, 3.20) | 0.001 | 49/79,863 | 2.19 (1.63, 2.95) | <0.001 | 59/169,738 | 2.39 (1.83, 3.13) | <0.001 |
| **By type of viral infection** |  |  |  |  |  |  |  |  |  |
| Acute viral infections | 233/346,133 | Ref |  | 451/1,729,205 | Ref |  | 717/4,229,474 | Ref |  |
|  | 21/12,795 | 2.28 (1.46, 3.57) | <0.001 | 40/63,858 | 2.21 (1.59, 3.05) | <0.001 | 49/129,935 | 2.56 (1.91, 3.43) | <0.001 |
| Herpesvirus (persistent) infections | 249/356,683 | Ref |  | 483/1,781,865 | Ref |  | 755/4,336,454 | Ref |  |
|  | 5/2,245 | 3.11 (1.28, 7.54) | 0.012 | 8/11,197 | 2.42 (1.20, 4.86) | 0.013 | 11/22,955 | 3.17 (1.75, 5.76) | <0.001 |
| Other persistent viral infections | 253/357,349 | Ref |  | 487/1,785,176 | Ref |  | 764/4,334,072 | Ref |  |
|  | 1/1,579 | 0.78 (0.11, 5.56) | 0.803 | 4/7,886 | 1.78 (0.66, 4.78) | 0.252 | 2/25,336 | 0.50 (0.12, 2.00) | 0.327 |
| **Parasitic infections** | 253/358,104 | Ref |  | 486/1,788,951 | Ref |  | 764/4,349,427 | Ref |  |
|  | 1/825 | 1.61 (0.23, 11.53) | 0.633 | 5/4,111 | 4.30 (1.78, 10.40) | 0.001 | 2/9,982 | 1.26 (0.31, 5.04) | 0.747 |
| **Fungal infections** | 240/351,235 | Ref |  | 469/1,754,659 | Ref |  | 743/4,295,355 | Ref |  |
|  | 14/7,693 | 2.50 (1.45, 4.29) | 0.001 | 22/38,403 | 1.88 (1.22, 2.88) | 0.004 | 23/64,054 | 2.30 (1.52, 3.50) | <0.001 |
| **By type of Fungal infections** |  |  |  |  |  |  |  |  |  |
| Superficial fungal infections | 247/354,540 | Ref |  | 483/1,771,148 | Ref |  | 751/4,324,909 | Ref |  |
|  | 7/4,388 | 2.15 (1.01, 4.58) | 0.046 | 8/21,915 | 1.17 (0.58, 2.37) | 0.652 | 15/34,500 | 2.80 (1.68, 4.68) | <0.001 |
| Deep fungal infections | 247/355,301 | Ref |  | 475/1,774,964 | Ref |  | 757/4,326,802 | Ref |  |
|  | 7/3,628 | 2.56 (1.20, 5.43) | 0.015 | 16/18,098 | 2.86 (1.74, 4.72) | <0.001 | 9/32,606 | 1.71 (0.89, 3.31) | 0.110 |

CI, confidence interval; HR, hazard ratio; UC, ulcerative colitis.

Based on the fully-adjusted model, adjusted for age, sex, ethnic background, education level, TDI, BMI, smoking status, alcohol consumption, physical activity.

**Table S40.** Sensitivity analyses assessing the associations between infectious diseases and IBD, CD, and UC accounting for the competing risk of death.

| **Exposure** | **IBD** | | **CD** | | **UC** | |
| --- | --- | --- | --- | --- | --- | --- |
|  | **HR (95%CI)** | ***P*** | **HR (95%CI)** | ***P*** | **HR (95%CI)** | ***P*** |
| **All infectious** | 3.76 (3.46, 4.08) | <0.001 | 5.78 (5.01, 6.68) | <0.001 | 3.71 (3.37, 4.08) | <0.001 |
| **Bacterial infections** | 3.69 (3.40, 4.00) | <0.001 | 5.53 (4.81, 6.34) | <0.001 | 3.61 (3.29, 3.96) | <0.001 |
| **By severity** |  |  |  |  |  |  |
| Invasive bacterial infections | 2.07 (1.91, 2.25) | <0.001 | 2.50 (2.20, 2.84) | <0.001 | 2.04 (1.85, 2.24) | <0.001 |
| Localized bacterial infections | 3.61 (3.34, 3.90) | <0.001 | 4.76 (4.20, 5.40) | <0.001 | 3.61 (3.30, 3.95) | <0.001 |
| Bacterial infections with sepsis | 2.26 (1.98, 2.57) | <0.001 | 2.55 (2.10, 3.11) | <0.001 | 2.13 (1.83, 2.49) | <0.001 |
| Bacterial infections without sepsis | 3.63 (3.35, 3.93) | <0.001 | 5.32 (4.64, 6.10) | <0.001 | 3.59 (3.27, 3.94) | <0.001 |
| **By location** |  |  |  |  |  |  |
| Extracellular bacterial infections | 2.12 (1.90, 2.37) | <0.001 | 2.30 (1.94, 2.72) | <0.001 | 2.20 (1.94, 2.49) | <0.001 |
| Intracellular bacterial infections | 3.65 (2.78, 4.79) | <0.001 | 4.70 (3.19, 6.91) | <0.001 | 3.46 (2.50, 4.78) | <0.001 |
| **By Gram stain** |  |  |  |  |  |  |
| Gram-positive infections | 2.03 (1.75, 2.37) | <0.001 | 1.99 (1.57, 2.53) | <0.001 | 2.20 (1.86, 2.61) | <0.001 |
| Gram-negative infections | 2.33 (2.08, 2.63) | <0.001 | 2.69 (2.25, 3.21) | <0.001 | 2.39 (2.09, 2.74) | <0.001 |
| **Viral infections** | 1.93 (1.69, 2.19) | <0.001 | 1.98 (1.62, 2.42) | <0.001 | 1.99 (1.71, 2.31) | <0.001 |
| **By type of viral infection** |  |  |  |  |  |  |
| Acute viral infections | 1.89 (1.64, 2.18) | <0.001 | 1.78 (1.41, 2.24) | <0.001 | 2.03 (1.73, 2.39) | <0.001 |
| Herpesvirus (persistent) infections | 2.64 (1.99, 3.50) | <0.001 | 3.18 (2.12, 4.77) | <0.001 | 2.65 (1.91, 3.68) | <0.001 |
| Other persistent viral infections | 1.23 (0.76, 1.98) | 0.410 | 1.58 (0.81, 3.05) | 0.180 | 0.99 (0.53, 1.85) | 0.970 |
| **Parasitic infections** | 2.36 (1.44, 3.87) | <0.001 | 3.81 (2.04, 7.13) | <0.001 | 2.19 (1.21, 3.97) | 0.010 |
| **Fungal infections** | 2.08 (1.75, 2.47) | <0.001 | 2.91 (2.30, 3.68) | <0.001 | 1.82 (1.47, 2.25) | <0.001 |
| **By type of Fungal infections** |  |  |  |  |  |  |
| Superficial fungal infections | 2.02 (1.61, 2.53) | <0.001 | 2.82 (2.08, 3.82) | <0.001 | 1.72 (1.29, 2.29) | <0.001 |
| Deep fungal infections | 2.11 (1.66, 2.69) | <0.001 | 2.90 (2.08, 4.03) | <0.001 | 1.85 (1.37, 2.50) | <0.001 |

CI, confidence interval; HR, hazard ratio; IBD, inflammatory bowel disease; CD, Crohn's disease; UC, ulcerative colitis.

Based on the fully-adjusted model, adjusted for age, sex, ethnic background, education level, TDI, BMI, smoking status, alcohol consumption, physical activity.
**Table S41.** Sensitivity analysis of the associations between infectious diseases and risk of IBD, CD, and UC, with follow-up restricted to the pre–COVID-19 period**.**

| **Exposure** | **IBD** | | **CD** | | **UC** | |
| --- | --- | --- | --- | --- | --- | --- |
|  | **HR (95%CI)** | ***P*** | **HR (95%CI)** | ***P*** | **HR (95%CI)** | ***P*** |
| **All infectious** | 3.85 (3.47, 4.26) | <0.001 | 6.22 (5.22, 7.43) | <0.001 | 3.69 (3.27, 4.16) | <0.001 |
| **Bacterial infections** | 3.98 (3.59, 4.41) | <0.001 | 6.24 (5.25, 7.43) | <0.001 | 3.82 (3.39, 4.31) | <0.001 |
| **By severity** |  |  |  |  |  |  |
| Invasive bacterial infections | 2.75 (2.46, 3.07) | <0.001 | 3.44 (2.89, 4.09) | <0.001 | 2.73 (2.40, 3.12) | <0.001 |
| Localized bacterial infections | 4.20 (3.79, 4.66) | <0.001 | 6.08 (5.14, 7.18) | <0.001 | 4.09 (3.62, 4.61) | <0.001 |
| Bacterial infections with sepsis | 3.22 (2.67, 3.88) | <0.001 | 3.77 (2.85, 4.99) | <0.001 | 3.00 (2.39, 3.75) | <0.001 |
| Bacterial infections without sepsis | 3.99 (3.60, 4.42) | <0.001 | 6.16 (5.18, 7.32) | <0.001 | 3.88 (3.44, 4.37) | <0.001 |
| **By location** |  |  |  |  |  |  |
| Extracellular bacterial infections | 2.87 (2.47, 3.34) | <0.001 | 3.29 (2.61, 4.14) | <0.001 | 2.94 (2.47, 3.51) | <0.001 |
| Intracellular bacterial infections | 5.47 (3.94, 7.59) | <0.001 | 8.19 (5.28, 12.68) | <0.001 | 5.00 (3.36, 7.44) | <0.001 |
| **By Gram stain** |  |  |  |  |  |  |
| Gram-positive infections | 2.57 (2.09, 3.15) | <0.001 | 2.56 (1.84, 3.55) | <0.001 | 2.81 (2.23, 3.54) | <0.001 |
| Gram-negative infections | 3.48 (2.97, 4.09) | <0.001 | 4.56 (3.62, 5.76) | <0.001 | 3.46 (2.87, 4.18) | <0.001 |
| **Viral infections** | 2.49 (2.08, 2.99) | <0.001 | 2.87 (2.18, 3.77) | <0.001 | 2.54 (2.06, 3.14) | <0.001 |
| **By type of viral infection** |  |  |  |  |  |  |
| Acute viral infections | 2.64 (2.17, 3.22) | <0.001 | 2.82 (2.08, 3.83) | <0.001 | 2.74 (2.18, 3.44) | <0.001 |
| Herpesvirus (persistent) infections | 2.73 (1.78, 4.21) | <0.001 | 3.64 (2.00, 6.61) | <0.001 | 3.03 (1.87, 4.89) | <0.001 |
| Other persistent viral infections | 1.22 (0.63, 2.36) | 0.550 | 2.00 (0.89, 4.48) | 0.094 | 0.93 (0.39, 2.24) | 0.871 |
| **Parasitic infections** | 4.21 (2.44, 7.28) | <0.001 | 7.68 (3.97, 14.89) | <0.001 | 3.49 (1.74, 7.01) | <0.001 |
| **Fungal infections** | 2.70 (2.11, 3.46) | <0.001 | 3.71 (2.64, 5.22) | <0.001 | 2.52 (1.87, 3.40) | <0.001 |
| **By type of Fungal infections** |  |  |  |  |  |  |
| Superficial fungal infections | 2.62 (1.88, 3.65) | <0.001 | 2.85 (1.73, 4.69) | <0.001 | 2.61 (1.77, 3.86) | <0.001 |
| Deep fungal infections | 2.66 (1.88, 3.76) | <0.001 | 4.09 (2.61, 6.41) | <0.001 | 2.41 (1.58, 3.69) | <0.001 |

CI, confidence interval; HR, hazard ratio; IBD, inflammatory bowel disease; CD, Crohn's disease; UC, ulcerative colitis.

Based on the fully-adjusted model, adjusted for age, sex, ethnic background, education level, TDI, BMI, smoking status, alcohol consumption, physical activity.

## Table S42. Sensitivity analysis of the associations between infection and incident IBD, CD, and UC using E-values.

| **Disease** | IBD | | | | CD | | | | UC | | | |
| --- | --- | --- | --- | --- | --- | --- | --- | --- | --- | --- | --- | --- |
|  | aHR Point Estimate | aHR 95% CI Lower limit | E value (aHR Point Estimate) | E value (aHR 95% CI Lower limit) | aHR Point Estimate | aHR 95% CI Lower limit | E value (aHR Point Estimate) | E value (aHR 95% CI Lower limit) | aHR Point Estimate | aHR 95% CI Lower limit | E value (aHR Point Estimate) | E value (aHR 95% CI Lower limit) |
| Any infectious diseases | 3.44 | 3.14 | 6.34 | 5.73 | 5.08 | 4.35 | 9.63 | 8.17 | 3.34 | 3.01 | 6.14 | 5.47 |
| Any bacterial infections | 3.54 | 3.24 | 6.54 | 5.93 | 5.20 | 4.46 | 9.87 | 8.39 | 3.42 | 3.08 | 6.30 | 5.61 |
| Invasive bacterial infections | 2.36 | 2.14 | 4.15 | 3.70 | 2.80 | 2.4 | 5.04 | 4.23 | 2.40 | 2.14 | 4.23 | 3.70 |
| Localized bacterial infections | 3.78 | 3.45 | 7.02 | 6.36 | 5.30 | 4.57 | 10.07 | 8.61 | 3.66 | 3.29 | 6.78 | 6.03 |
| Bacterial infections with sepsis | 3.01 | 2.56 | 5.47 | 4.56 | 3.45 | 2.69 | 6.36 | 4.82 | 2.82 | 2.31 | 5.09 | 4.05 |
| Bacterial infections without sepsis | 3.55 | 3.25 | 6.56 | 5.95 | 5.15 | 4.42 | 9.77 | 8.31 | 3.45 | 3.11 | 6.36 | 5.67 |
| Extracellular bacterial infections | 2.61 | 2.29 | 4.66 | 4.01 | 2.81 | 2.28 | 5.07 | 3.99 | 2.76 | 2.37 | 4.96 | 4.17 |
| Intracellular bacterial infections | 4.09 | 2.99 | 7.65 | 5.43 | 6.06 | 3.96 | 11.60 | 7.38 | 3.91 | 2.69 | 7.28 | 4.82 |
| Gram-positive infections | 2.41 | 2.01 | 4.25 | 3.43 | 2.27 | 1.69 | 3.97 | 2.77 | 2.72 | 2.22 | 4.88 | 3.87 |
| Gram-negative infections | 3.04 | 2.64 | 5.53 | 4.72 | 3.70 | 2.99 | 6.86 | 5.43 | 3.11 | 2.64 | 5.67 | 4.72 |
| Any viral infections | 2.24 | 1.91 | 3.91 | 3.23 | 2.30 | 1.57 | 4.03 | 2.52 | 2.37 | 1.98 | 4.17 | 3.37 |
| Acute viral infections | 2.27 | 1.91 | 3.97 | 3.23 | 2.09 | 1.57 | 3.60 | 2.52 | 2.50 | 2.05 | 4.44 | 3.52 |
| Herpesvirus persistent infections | 2.89 | 2.04 | 5.23 | 3.50 | 3.97 | 2.45 | 7.40 | 4.33 | 3.01 | 2.01 | 5.47 | 3.43 |
| Other persistent viral infections | 1.14 | 0.64 | 1.54 | 2.50 | 1.66 | 0.79 | 2.71 | 1.85 | 0.93 | 0.44 | 1.36 | 3.97 |
| Any parasitic infections | 2.87 | 1.66 | 5.19 | 2.71 | 5.38 | 2.78 | 10.23 | 5.00 | 2.40 | 1.20 | 4.23 | 1.69 |
| Any fungal infections | 2.63 | 2.12 | 4.70 | 3.66 | 3.63 | 2.7 | 6.72 | 4.84 | 2.41 | 1.86 | 4.25 | 3.12 |
| Superficial fungal infections | 2.48 | 1.85 | 4.40 | 3.10 | 3.36 | 2.25 | 6.18 | 3.93 | 2.26 | 1.57 | 3.95 | 2.52 |
| Deep fungal infections | 2.73 | 2.05 | 4.90 | 3.52 | 3.66 | 2.45 | 6.78 | 4.33 | 2.51 | 1.76 | 4.46 | 2.92 |

CI, confidence interval; HR, hazard ratio; IBD, inflammatory bowel disease; CD, Crohn's disease; UC, ulcerative colitis.

Based on the fully-adjusted model, adjusted for age, sex, ethnic background, education level, TDI, BMI, smoking status, alcohol consumption, physical activity.

To assess the robustness of the observed associations to potential unmeasured confounding, we calculated E-values for the point estimates and the confidence limits closest to the null according to published methods. For aHRs greater than 1, the lower limit of the 95% CI was used; for aHRs less than 1, the upper limit of the 95% CI was used.

## Table S43. Classification of type-specific infectious diseases in the study on diagnosis level.

| **Infection type** | **ICD10 codes** |
| --- | --- |
| All infection | A00, A00.0, A00.1, A00.9, A01, A02, A02.0, A02.1, A02.2, A02.8, A02.9, A03, A03.0, A03.1, A03.2, A03.3, A03.8, A03.9, A04, A04.0, A04.1, A04.2, A04.3, A04.4, A04.5, A04.6, A04.7, A04.8, A04.9, A05, A05.0, A05.1, A05.2, A05.3, A05.4, A05.8, A05.9, A06, A06.0, A06.1, A06.2, A06.3, A06.4, A06.5, A06.6, A06.7, A06.8, A06.9, A07, A08.0, A08.1, A08.2, A08.3, A08.4, A08.5, A09, A15, A16, A17, A17.0, A17.1, A17.8, A17.9, A18, A19, A20, A20.0, A20.1, A20.2, A20.3, A20.7, A20.8, A20.9, A21, A21.0, A21.1, A21.2, A21.3, A21.7, A21.8, A21.9, A22, A22.0, A22.1, A22.2, A22.7, A22.8, A22.9, A23, A23.0, A23.1, A23.2, A23.3, A23.8, A23.9, A24, A24.0, A24.1, A24.2, A24.3, A25, A25.0, A25.1, A25.9, A26, A26.0, A26.7, A26.8, A26.9, A27, A28, A28.0, A28.1, A28.2, A28.8, A28.9, A30, A31, A32, A32.0, A32.1, A32.7, A32.8, A32.9, A33, A34, A35, A36, A37, A38, A39, A39.0, A39.1, A39.2, A39.3, A39.4, A39.5, A39.8, A39.9, A40, A40.0, A40.1, A40.2, A40.3, A40.8, A40.9, A41, A41.0, A41.1, A41.2, A41.3, A41.4, A41.5, A41.8, A41.9, A42, A42.0, A42.1, A42.2, A42.7, A42.8, A42.9, A43, A43.0, A43.1, A43.8, A44, A44.0, A44.1, A44.8, A44.9, A46, A48.0, A48.1, A48.2, A48.3, A48.4, A48.8, A49, A49.0, A49.1, A49.2, A49.3, A49.8, A49.9, A50, A50.0, A50.1, A50.2, A50.3, A50.4, A50.5, A50.6, A50.7, A50.9, A51, A51.0, A51.1, A51.2, A51.3, A51.4, A51.5, A51.9, A52, A52.0, A52.1, A52.2, A52.3, A52.7, A52.8, A52.9, A53, A54, A54.0, A54.1, A54.2, A54.3, A54.4, A54.5, A54.6, A54.8, A54.9, A55, A56.0, A56.1, A56.2, A56.3, A56.4, A56.8, A57, A58, A59, A60, A63.0, A63.8, A64, A65, A66, A67, A68, A69, A69.0, A69.1, A69.2, A69.8, A69.9, A70, A71, A74.0, A74.8, A74.9, A75, A75.0, A75.1, A75.2, A75.3, A75.9, A77, A77.0, A77.1, A77.2, A77.3, A77.8, A77.9, A78, A79, A79.0, A79.1, A79.8, A79.9, A80, A81.1, A81.2, A83, A84, A85, A86, A87, A88, A89, A90, A91, A92, A92.0, A92.1, A92.2, A92.3, A92.4, A92.8, A92.9, A93, A93.0, A93.1, A93.2, A93.8, A94, A95, A96, A97, A98, A98.0, A98.1, A98.2, A98.3, A98.4, A98.5, A98.8, A99, B00, B00.0, B00.1, B00.2, B00.3, B00.4, B00.5, B00.7, B00.8, B00.9, B01, B01.0, B01.1, B01.2, B01.8, B01.9, B02, B02.0, B02.1, B02.2, B02.3, B02.7, B02.8, B02.9, B04, B05, B05.0, B05.1, B05.2, B05.3, B05.4, B05.8, B05.9, B06, B06.0, B06.8, B06.9, B07, B08.0, B08.1, B08.2, B08.3, B08.4, B08.5, B08.8, B09, B15, B15.0, B15.9, B16, B16.0, B16.1, B16.2, B16.9, B17, B18, B19, B19.0, B19.9, B20, B21, B21.0, B22, B22.0, B22.1, B22.2, B22.7, B23, B24, B25, B26, B26.0, B26.1, B26.2, B26.3, B26.8, B26.9, B27, B27.0, B27.1, B27.8, B27.9, B30, B33, B33.0, B33.1, B33.2, B33.3, B33.4, B33.8, B34, B34.0, B34.1, B34.2, B34.3, B34.4, B34.8, B34.9, B35, B36, B37, B37.0, B37.1, B37.2, B37.3, B37.4, B37.5, B37.6, B37.7, B37.8, B37.9, B38, B38.0, B38.1, B38.2, B38.3, B38.4, B38.7, B38.8, B38.9, B39, B39.0, B39.1, B39.2, B39.3, B39.4, B39.5, B39.9, B40, B40.0, B40.1, B40.2, B40.3, B40.7, B40.8, B40.9, B41, B41.0, B41.7, B41.8, B41.9, B42, B42.0, B42.1, B42.7, B42.8, B42.9, B43, B43.0, B43.1, B43.2, B43.8, B43.9, B44, B45, B45.0, B45.1, B45.2, B45.3, B45.7, B45.8, B45.9, B46, B46.0, B46.1, B46.2, B46.3, B46.4, B46.5, B46.8, B46.9, B47, B47.0, B47.1, B47.9, B48, B48.0, B48.1, B48.2, B48.3, B48.4, B48.7, B48.8, B49, B50, B50.0, B50.8, B50.9, B51, B52, B53, B53.0, B53.1, B53.8, B54, B55.0, B55.1, B55.2, B55.9, B56, B57, B58, B58.0, B58.1, B58.2, B58.3, B58.8, B58.9, B59, B60.0, B60.1, B60.2, B60.8, B64, B65, B65.0, B65.1, B65.2, B65.3, B65.8, B65.9, B66, B66.0, B66.1, B66.2, B66.3, B66.4, B66.5, B66.8, B66.9, B67, B68, B68.0, B68.1, B68.9, B69, B69.0, B69.1, B69.8, B69.9, B70, B70.0, B70.1, B71, B71.0, B71.1, B71.8, B71.9, B72, B73, B74, B75, B76, B77, B78, B79, B80, B81, B81.0, B81.1, B81.2, B81.3, B81.4, B81.8, B82, B82.0, B82.9, B83, B83.0, B83.1, B83.2, B83.3, B83.4, B83.8, B83.9, B85, B86, B87, B88, B88.0, B88.1, B88.2, B88.3, B88.8, B88.9, B89, B95, B96.0, B96.1, B96.2, B96.3, B96.4, B96.5, B96.6, B96.7, B96.8, B97, B97.0, B97.1, B97.2, B97.3, B97.4, B97.5, B97.6, B97.7, B97.8, B98.0, B98.1, B99, C46, D73.3, E32.1, G00, G00.0, G00.1, G00.2, G00.3, G00.8, G00.9, G01, G02.0, G02.1, G02.8, G03, G04.1, G04.2, G05.0, G05.1, G05.2, G06, G07, H00, H01.0, H05.0, H06.1, H10.0, H10.5, H13.0, H19.0, H19.1, H19.2, H22.0, H32.0, H44.0, H60.0, H60.1, H60.2, H60.3, H62.0, H62.1, H62.2, H62.3, H66.0, H67.0, H67.1, H70.0, H75.0, I30.1, I32.0, I32.1, I33.0, I40.0, I41.0, I41.1, I41.2, I43.0, I52.0, I52.1, I68.1, J01.0, J02, J02.0, J02.8, J02.9, J03, J03.0, J03.8, J03.9, J04, J05.1, J09, J10, J11, J12, J13, J14, J15, J15.0, J15.1, J15.2, J15.3, J15.4, J15.5, J15.6, J15.7, J15.8, J15.9, J16, J16.0, J16.8, J17.0, J17.1, J17.2, J17.3, J17.8, J18, J20, J20.0, J20.1, J20.2, J20.3, J20.4, J20.5, J20.6, J20.7, J20.8, J20.9, J21, J21.0, J21.1, J21.8, J21.9, J22, J36, J39.0, J39.1, J85.1, J85.2, J85.3, J86, K02, K04.4, K04.5, K04.6, K04.7, K05.0, K05.2, K05.3, K05.4, K11.3, K12.2, K23.0, K23.1, K35, K57.0, K57.2, K57.4, K57.8, K61, K63.0, K65.0, K67.0, K67.1, K67.2, K67.3, K67.8, K75.0, K77.0, L00, L01, L02, L03, L04, L05, L08, L70.1, M00, M00.0, M00.1, M00.2, M00.8, M00.9, M01.0, M01.1, M01.2, M01.3, M01.4, M01.5, M01.6, M01.8, M46.2, M46.3, M46.5, M49.0, M49.1, M49.2, M49.3, M60.0, M63.0, M63.1, M63.2, M65.0, M65.1, M71.0, M71.1, M72.6, M73.0, M73.1, M86, M86.0, M86.1, M86.2, M86.3, M86.4, M86.5, M86.6, M86.8, M86.9, N08.0, N10, N13.6, N15.1, N16.0, N29.0, N29.1, N30.0, N34.0, N39.0, N41.0, N41.2, N45, N61, N70.0, N71.0, N73.0, N73.3, N74, N74.0, N74.1, N74.2, N74.3, N74.4, N74.8, N75.1, N76.0, N76.4, N77.0, N77.1, O03.0, O03.5, O04.0, O04.5, O05.0, O05.5, O06.0, O06.5, O07.0, O07.5, O08.0, O23.0, O23.1, O23.2, O23.3, O23.4, O23.5, O23.9, O75.3, O85, O86, O91.0, O91.1, O98, O98.0, O98.1, O98.2, O98.3, O98.4, O98.5, O98.6, O98.7, O98.8, O98.9, P23, P23.0, P23.1, P23.2, P23.3, P23.4, P23.5, P23.6, P23.8, P23.9, P35.0, P35.1, P35.2, P35.3, P36, P36.0, P36.1, P36.2, P36.3, P36.4, P36.5, P36.8, P36.9, P37, P37.0, P37.1, P37.2, P37.3, P37.4, P37.5, P37.8, P37.9, P38, P39, P39.0, P39.1, P39.2, P39.3, P39.4, P39.8, P39.9, R57.2, R65.0, R65.1, R78.8 |
| All bacterial infections | A00, A01, A02, A02.0, A02.1, A02.2, A02.8, A02.9, A03, A03.0, A03.1, A03.2, A03.3, A03.8, A03.9, A04, A04.0, A04.1, A04.2, A04.3, A04.4, A04.5, A04.6, A04.7, A04.8, A04.9, A05, A05.0, A05.1, A05.2, A05.3, A05.4, A05.8, A05.9, A09, A15, A16, A17, A17.0, A17.1, A17.8, A17.9, A18, A19, A20, A20.0, A20.1, A20.2, A20.3, A20.7, A20.8, A20.9, A21, A21.0, A21.1, A21.2, A21.3, A21.7, A21.8, A21.9, A22, A22.0, A22.1, A22.2, A22.7, A22.8, A22.9, A23, A23.0, A23.1, A23.2, A23.3, A23.8, A23.9, A24, A24.0, A24.1, A24.2, A24.3, A25, A25.0, A25.1, A25.9, A26, A26.0, A26.7, A26.8, A26.9, A27, A28, A28.0, A28.1, A28.2, A28.8, A28.9, A30, A31, A32, A32.0, A32.1, A32.7, A32.8, A32.9, A33, A34, A35, A36, A37, A38, A39, A39.0, A39.1, A39.2, A39.3, A39.4, A39.5, A39.8, A39.9, A40, A40.0, A40.1, A40.2, A40.3, A40.8, A40.9, A41, A41.0, A41.1, A41.2, A41.3, A41.4, A41.5, A41.8, A41.9, A42, A42.0, A42.1, A42.2, A42.7, A42.8, A42.9, A43, A43.0, A43.1, A43.8, A44, A44.0, A44.1, A44.8, A44.9, A46, A48.0, A48.1, A48.2, A48.3, A48.4, A48.8, A49, A49.0, A49.1, A49.2, A49.3, A49.8, A49.9, A50, A50.0, A50.1, A50.2, A50.3, A50.4, A50.5, A50.6, A50.7, A50.9, A51, A51.0, A51.1, A51.2, A51.3, A51.4, A51.5, A51.9, A52, A52.0, A52.1, A52.2, A52.3, A52.7, A52.8, A52.9, A53, A54, A54.0, A54.1, A54.2, A54.3, A54.4, A54.5, A54.6, A54.8, A54.9, A55, A56.0, A56.1, A56.2, A56.3, A56.4, A56.8, A57, A58, A64, A65, A66, A67, A68, A69, A69.0, A69.1, A69.2, A69.8, A69.9, A70, A71, A74.0, A74.8, A74.9, A75, A75.0, A75.1, A75.2, A75.3, A75.9, A77, A77.0, A77.1, A77.2, A77.3, A77.8, A77.9, A78, A79, A79.0, A79.1, A79.8, A79.9, B47.1, B95, B96.0, B96.1, B96.2, B96.3, B96.4, B96.5, B96.6, B96.7, B96.8, B98.0, B98.1, D73.3, E32.1, G00, G00.0, G00.1, G00.2, G00.3, G00.8, G00.9, G01, G04.2, G05.0, G06, H00, H01.0, H05.0, H10.0, H10.5, H44.0, H60.0, H60.1, H60.2, H60.3, H62.0, H66.0, H67.0, H70.0, I32.0, I33.0, I41.0, I52.0, J01.0, J02, J02.0, J02.8, J02.9, J03, J03.0, J03.8, J03.9, J04, J05.1, J13, J14, J15, J15.0, J15.1, J15.2, J15.3, J15.4, J15.5, J15.6, J15.7, J15.8, J15.9, J16, J16.0, J16.8, J17.0, J17.8, J18, J20.0, J20.1, J20.2, J22, J36, J39.0, J39.1, J85.1, J85.2, J85.3, J86, K02, K04.4, K04.5, K04.6, K04.7, K05.0, K05.2, K05.3, K05.4, K11.3, K12.2, K23.0, K35, K57.0, K57.2, K57.4, K57.8, K61, K63.0, K65.0, K67.0, K67.1, K67.2, K67.3, K75.0, L00, L01, L02, L03, L04, L05, L08, L70.1, M00, M00.0, M00.1, M00.2, M00.8, M00.9, M01.0, M01.1, M01.2, M01.3, M46.2, M46.3, M46.5, M49.0, M49.1, M49.2, M63.0, M65.0, M65.1, M71.0, M71.1, M72.6, M73.0, M73.1, M86, M86.0, M86.1, M86.2, M86.3, M86.4, M86.5, M86.6, M86.8, M86.9, N10, N13.6, N15.1, N29.0, N30.0, N34.0, N39.0, N41.0, N41.2, N45, N61, N70.0, N71.0, N73.0, N73.3, N74.0, N74.1, N74.2, N74.3, N74.4, N75.1, N76.0, N76.4, O03.0, O03.5, O04.0, O04.5, O05.0, O05.5, O06.0, O06.5, O07.0, O07.5, O08.0, O23.0, O23.1, O23.2, O23.3, O23.4, O23.5, O23.9, O75.3, O85, O86, O91.0, O91.1, O98.0, O98.1, O98.2, O98.3, O98.8, O98.9, P23, P23.1, P23.2, P23.3, P23.4, P23.5, P23.6, P23.9, P36, P36.0, P36.1, P36.2, P36.3, P36.4, P36.5, P36.8, P36.9, P37.0, P37.2, P38, P39.0, P39.1, P39.2, P39.3, P39.4 |
| Invasive bacterial infections | A01, A02, A02.0, A02.1, A02.2, A02.8, A02.9, A03, A03.0, A03.1, A03.2, A03.3, A03.8, A03.9, A15, A16, A17, A17.0, A17.1, A17.8, A17.9, A18, A19, A20, A20.0, A20.1, A20.2, A20.3, A20.7, A20.8, A20.9, A21, A21.0, A21.1, A21.2, A21.3, A21.7, A21.8, A21.9, A22, A22.0, A22.1, A22.2, A22.7, A22.8, A22.9, A23, A23.0, A23.1, A23.2, A23.3, A23.8, A23.9, A24, A24.0, A24.1, A24.2, A24.3, A25, A25.0, A25.1, A25.9, A26.7, A26.8, A27, A28, A28.0, A28.1, A28.2, A28.8, A28.9, A30, A31, A32, A32.0, A32.1, A32.7, A32.8, A32.9, A33, A34, A35, A36, A38, A39, A39.0, A39.1, A39.2, A39.3, A39.4, A39.5, A39.8, A39.9, A40, A40.0, A40.1, A40.2, A40.3, A40.8, A40.9, A41, A41.0, A41.1, A41.2, A41.3, A41.4, A41.5, A41.8, A41.9, A42, A42.0, A42.1, A42.2, A42.7, A42.8, A42.9, A43, A43.0, A43.1, A43.8, A44, A44.0, A44.1, A44.8, A44.9, A46, A48.0, A48.1, A48.3, A48.4, A48.8, A49.0, A49.1, A49.2, A49.8, A49.9, A50, A50.0, A50.1, A50.2, A50.3, A50.4, A50.5, A50.6, A50.7, A50.9, A51, A51.0, A51.1, A51.2, A51.3, A51.4, A51.5, A51.9, A52, A52.0, A52.1, A52.2, A52.3, A52.7, A52.8, A52.9, A53, A54.1, A54.2, A54.4, A54.8, A55, A56.1, A65, A66, A67, A68, A69, A69.0, A69.1, A69.2, A69.8, A69.9, A70, A75, A75.0, A75.1, A75.2, A75.3, A75.9, A77, A77.0, A77.1, A77.2, A77.3, A77.8, A77.9, A78, A79, A79.0, A79.1, A79.8, A79.9, B47.1, B95, B96.1, B96.2, B96.3, B96.4, B96.5, B96.6, B96.7, B96.8, B98.1, D73.3, E32.1, G00, G00.0, G00.1, G00.2, G00.3, G00.8, G00.9, G01, G04.2, G05.0, G06, H05.0, H44.0, H60.0, H60.1, H60.2, H70.0, I32.0, I33.0, I41.0, I52.0, J05.1, J13, J14, J15, J15.0, J15.1, J15.2, J15.3, J15.4, J15.5, J15.6, J15.7, J15.8, J15.9, J16, J16.0, J16.8, J17.0, J17.8, J18, J36, J39.0, J39.1, J85.1, J85.2, J85.3, J86, K12.2, K23.0, K35, K57.0, K57.2, K57.4, K57.8, K61, K63.0, K65.0, K67.0, K67.1, K67.2, K67.3, K75.0, M00, M00.0, M00.1, M00.2, M00.8, M00.9, M01.0, M01.1, M01.2, M01.3, M46.2, M46.3, M46.5, M49.0, M49.1, M49.2, M63.0, M65.0, M65.1, M71.0, M71.1, M72.6, M73.0, M73.1, M86, M86.0, M86.1, M86.2, M86.3, M86.4, M86.5, M86.6, M86.8, M86.9, N10, N13.6, N15.1, N29.0, N34.0, N41.2, N45, N61, N70.0, N71.0, N73.0, N73.3, N74.0, N74.1, N74.2, N74.3, N74.4, N75.1, N76.4, O03.0, O03.5, O04.0, O04.5, O05.0, O05.5, O06.0, O06.5, O07.0, O07.5, O08.0, O23.0, O75.3, O85, O91.1, O98.0, O98.1, P23, P23.1, P23.2, P23.3, P23.4, P23.5, P23.6, P23.9, P36, P36.0, P36.1, P36.2, P36.3, P36.4, P36.5, P36.8, P36.9, P37.0, P37.2, P39.0, P39.2, P39.3 |
| Localised bacterial infections | A00, A04, A04.0, A04.1, A04.2, A04.3, A04.4, A04.5, A04.6, A04.7, A04.8, A04.9, A05, A05.0, A05.1, A05.2, A05.3, A05.4, A05.8, A05.9, A09, A26, A26.0, A26.9, A37, A48.2, A54.0, A54.3, A54.5, A54.6, A56.0, A56.3, A56.4, A57, A58, A64, A71, A74.0, B98.0, H00, H01.0, H10.0, H10.5, H60.3, H66.0, H67.0, J01.0, J02, J02.0, J02.8, J02.9, J03, J03.0, J03.8, J03.9, J04, J20.0, J20.1, J20.2, J22, K02, K04.4, K04.5, K04.6, K04.7, K05.0, K05.2, K05.3, K05.4, K11.3, L00, L01, L02, L03, L04, L05, L08, L70.1, N30.0, N39.0, N41.0, N76.0, O23.1, O23.2, O23.3, O23.4, O23.5, O23.9, O86, O91.0, O98.2, O98.3, O98.8, O98.9, P38, P39.1, P39.4, A02.1, A20.7, A21.7, A22.7, A32.7, A39.1, A39.2, A39.3, A39.4, A40, A40.0, A40.1, A40.2, A40.3, A40.8, A40.9, A41, A41.0, A41.1, A41.2, A41.3, A41.4, A41.5, A41.8, A41.9, A42.7, A44.0, A48.3, A48.4, P36, P36.0, P36.1, P36.2, P36.3, P36.4, P36.5, P36.8, P36.9, P37.2, A01, A02, A02.0, A02.2, A02.8, A02.9, A03, A03.0, A03.1, A03.2, A03.3, A03.8, A03.9, A15, A16, A17, A17.0, A17.1, A17.8, A17.9, A18, A19, A20, A20.0, A20.1, A20.2, A20.3, A20.8, A20.9, A21, A21.0, A21.1, A21.2, A21.3, A21.8, A21.9, A22, A22.0, A22.1, A22.2, A22.8, A22.9, A23, A23.0, A23.1, A23.2, A23.3, A23.8, A23.9, A24, A24.0, A24.1, A24.2, A24.3, A25, A25.0, A25.1, A25.9, A26.7, A26.8, A27, A28, A28.0, A28.1, A28.2, A28.8, A28.9, A30, A31, A32, A32.0, A32.1, A32.8, A32.9, A33, A34, A35, A36, A38, A39, A39.0, A39.5, A39.8, A39.9, A42, A42.0, A42.1, A42.2, A42.8, A42.9, A43, A43.0, A43.1, A43.8, A44, A44.1, A44.8, A44.9, A46, A48.0, A48.1, A48.8, A49, A49.0, A49.1, A49.2, A49.3, A49.8, A49.9, A50, A50.0, A50.1, A50.2, A50.3, A50.4, A50.5, A50.6, A50.7, A50.9, A51, A51.0, A51.1, A51.2, A51.3, A51.4, A51.5, A51.9, A52, A52.0, A52.1, A52.2, A52.3, A52.7, A52.8, A52.9, A53, A54, A54.1, A54.2, A54.4, A54.8, A54.9, A55, A56.1, A56.2, A56.8, A65, A66, A67, A68, A69, A69.0, A69.1, A69.2, A69.8, A69.9, A70, A74.8, A74.9, A75, A75.0, A75.1, A75.2, A75.3, A75.9, A77, A77.0, A77.1, A77.2, A77.3, A77.8, A77.9, A78, A79, A79.0, A79.1, A79.8, A79.9, B47.1, B95, B96.0, B96.1, B96.2, B96.3, B96.4, B96.5, B96.6, B96.7, B96.8, B98.1, D73.3, E32.1, G00, G00.0, G00.1, G00.2, G00.3, G00.8, G00.9, G01, G04.2, G05.0, G06, H05.0, H44.0, H60.0, H60.1, H60.2, H62.0, H70.0, I32.0, I33.0, I41.0, I52.0, J05.1, J13, J14, J15, J15.0, J15.1, J15.2, J15.3, J15.4, J15.5, J15.6, J15.7, J15.8, J15.9, J16, J16.0, J16.8, J17.0, J17.8, J18, J36, J39.0, J39.1, J85.1, J85.2, J85.3, J86, K12.2, K23.0, K35, K57.0, K57.2, K57.4, K57.8, K61, K63.0, K65.0, K67.0, K67.1, K67.2, K67.3, K75.0, M00, M00.0, M00.1, M00.2, M00.8, M00.9, M01.0, M01.1, M01.2, M01.3, M46.2, M46.3, M46.5, M49.0, M49.1, M49.2, M63.0, M65.0, M65.1, M71.0, M71.1, M72.6, M73.0, M73.1, M86, M86.0, M86.1, M86.2, M86.3, M86.4, M86.5, M86.6, M86.8, M86.9, N10, N13.6, N15.1, N29.0, N34.0, N41.2, N45, N61, N70.0, N71.0, N73.0, N73.3, N74.0, N74.1, N74.2, N74.3, N74.4, N75.1, N76.4, O03.0, O03.5, O04.0, O04.5, O05.0, O05.5, O06.0, O06.5, O07.0, O07.5, O08.0, O23.0, O75.3, O85, O91.1, O98.0, O98.1, P23, P23.1, P23.2, P23.3, P23.4, P23.5, P23.6, P23.9, P37.0, P39.0, P39.2, P39.3 |
| Intracellular bacterial infections | A01*, A02, A020, A021, A022, A028, A029, A03, A030, A031, A032, A033, A038, A039, A042, A045, A046, A15*, A16*, A17, A170, A171, A178, A179, A18*, A19*, A20, A200, A201, A202, A203, A207, A208, A209, A21, A210, A211, A212, A213, A217, A218, A219, A23, A230, A231, A232, A233, A238, A239, A24, A240, A241, A242, A243, A26, A260, A267, A268, A269, A281, A282, A30*, A31*, A32, A320, A321, A327, A328, A329, A43, A430, A431, A438, A44, A440, A441, A448, A449, A481, A482, A493, A55*, A560, A561, A562, A563, A564, A568, A58*, A70*, A71*, A740, A748, A749, A75, A750, A751, A752, A753, A759, A77, A770, A771, A772, A773, A778, A779, A78*, A79, A790, A791, A798, A799, B960, J157, J160, J200, K230, K670, K673, M011, M490, M491, N740, N741, N744, O980, P231, P370, P372 |
| Extracellular bacterial infections | A00*, A040, A041, A043, A044, A047, A050, A051, A052, A053, A054, A22, A220, A221, A222, A227, A228, A229, A25, A250, A251, A259, A27*, A280, A33*, A34*, A35*, A36*, A37*, A38*, A39, A390, A391, A392, A393, A394, A395, A398, A399, A40, A400, A401, A402, A403, A408, A409, A410, A411, A412, A413, A480, A483, A484, A490, A492, A50, A500, A501, A502, A503, A504, A505, A506, A507, A509, A51, A510, A511, A512, A513, A514, A515, A519, A52, A520, A521, A522, A523, A527, A528, A529, A53*, A54, A540, A541, A542, A543, A544, A545, A546, A548, A549, A57*, A65*, A66*, A67*, A68*, A692, A698, A699, B95*, B961, B962, B963, B964, B965, B966, B967, B980, B981, G000, G001, G002, G003, J020, J030, J13*, J14*, J150, J151, J152, J153, J154, J155, J201, J202, K671, K672, L00*, L01*, M000, M001, M002, M010, M012, M730, M731, N742, N743, O981, O982, P232, P233, P234, P235, P360, P361, P362, P363, P364 |
| Gram-positive bacterial infections | A047, A050, A051, A052, A054, A22, A220, A221, A222, A227, A228, A229, A26, A260, A267, A268, A269, A32, A320, A321, A327, A328, A329, A33*, A34*, A35*, A36*, A38*, A40, A400, A401, A402, A403, A408, A409, A410, A411, A412, A42, A420, A421, A422, A427, A428, A429, A43, A430, A431, A438, A480, A483, A490, A491, B471, B95*, B967, G001, G002, G003, J020, J030, J13*, J152, J153, J154, J202, L00*, L01*, M000, M001, M002, P232, P233, P360, P361, P362, P363, P372 |
| Gram-negative bacterial infections | A00*, A01*, A02, A020, A021, A022, A028, A029, A03, A030, A031, A032, A033, A038, A039, A040, A041, A042, A043, A044, A045, A046, A053, A20, A200, A201, A202, A203, A207, A208, A209, A21, A210, A211, A212, A213, A217, A218, A219, A23, A230, A231, A232, A233, A238, A239, A24, A240, A241, A242, A243, A25, A250, A251, A259, A27*, A280, A281, A282, A37*, A39, A390, A391, A392, A393, A394, A395, A398, A399, A413, A415, A44, A440, A441, A448, A449, A481, A482, A484, A492, A50, A500, A501, A502, A503, A504, A505, A506, A507, A509, A51, A510, A511, A512, A513, A514, A515, A519, A52, A520, A521, A522, A523, A527, A528, A529, A53*, A54, A540, A541, A542, A543, A544, A545, A546, A548, A549, A55*, A560, A561, A562, A563, A564, A568, A57*, A58*, A65*, A66*, A67*, A68*, A692, A698, A699, A70*, A71*, A740, A748, A749, A75, A750, A751, A752, A753, A759, A77, A770, A771, A772, A773, A778, A779, A78*, A79, A790, A791, A798, A799, B961, B962, B963, B964, B965, B966, B980, B981, G000, J14*, J150, J151, J155, J156, J160, J201, K670, K671, K672, M010, M012, M491, M492, M730, M731, N290, N742, N743, N744, O981, O982, P231, P234, P235, P364 |
| All viral infections | A080, A081, A082, A083, A084, A60*, A630, A80*, A811, A812, A83*, A84*, A85*, A86*, A87*, A88*, A89*, A90*, A91*, A92, A920, A921, A922, A923, A924, A928, A929, A93, A930, A931, A932, A938, A94*, A95*, A96*, A97*, A98, A980, A981, A982, A983, A984, A985, A988, A99*, B00, B000, B001, B002, B003, B004, B005, B007, B008, B009, B01, B010, B011, B012, B018, B019, B02, B020, B021, B022, B023, B027, B028, B029, B04*, B05, B050, B051, B052, B053, B054, B058, B059, B06, B060, B068, B069, B07*, B080, B081, B082, B083, B084, B085, B088, B09*, B15, B150, B159, B16, B160, B161, B162, B169, B17*, B18*, B19, B190, B199, B20*, B21*, B210, B22, B220, B221, B222, B227, B23*, B24*, B25*, B26, B260, B261, B262, B263, B268, B269, B27, B270, B271, B279, B30*, B33, B330, B331, B332, B333, B334, B338, B34, B340, B341, B342, B343, B344, B348, B349, B97, B970, B971, B972, B973, B974, B975, B976, B977, B978, C46, G020, G041, G051, H191, H621, H671, I411, J09*, J10*, J11*, J12*, J171, J203, J204, J205, J206, J207, J21*, J210, J211, J218, J219, M014, M015, O984, O985, O987, P230, P350, P351, P352, P353, Z21 |
| Acute viral infections | A080, A081, A082, A083, A084, A80*, A83*, A84*, A85*, A86*, A87*, A88*, A89*, A90*, A91*, A92, A920, A921, A922, A923, A924, A928, A929, A93, A930, A931, A932, A938, A94*, A95*, A96*, A97*, A98, A980, A981, A982, A983, A984, A985, A988, A99*, B04*, B05, B050, B051, B052, B053, B054, B058, B059, B06, B060, B068, B069, B07*, B080, B081, B083, B084, B085, B088, B09*, B15, B150, B159, B16, B160, B161, B162, B169, B17*, B19, B190, B199, B26, B260, B261, B262, B263, B268, B269, B30*, B33, B330, B331, B332, B334, B338, B34, B340, B341, B342, B343, B348, B349, B97, B970, B971, B972, B974, B975, B976, B978, H671, I411, J09*, J10*, J11*, J12*, J171, J203, J204, J205, J206, J207, J21*, J210, J211, J218, J219, M014, O985, P230 |
| Herpesvirus infections | A60*, B00, B000, B001, B002, B003, B004, B005, B007, B008, B009, B01, B010, B011, B012, B018, B019, B02, B020, B021, B022, B023, B027, B028, B029, B082, B210, B25*, B27, B270, B271, B279, C46, H191, P351, P352 |
| Other potentially persistent viral infections | A630, A811, A812, B18*, B20*, B21*, B22, B220, B221, B222, B227, B23*, B24*, B333, B344, B973, B977, G041, O984, O987, P353, Z21 |
| Parasitic infections | A06, A060, A061, A062, A063, A064, A065, A066, A067, A068, A069, A07*, A59*, B50*, B500, B508, B509, B51*, B52*, B53, B530, B531, B538, B54*, B550, B551, B552, B559, B56*, B57*, B58, B580, B581, B582, B583, B588, B589, B600, B601, B602, B608, B64*, B65, B650, B651, B652, B653, B658, B659, B66, B660, B661, B662, B663, B664, B665, B668, B669, B67*, B68, B680, B681, B689, B69, B690, B691, B698, B699, B70, B700, B701, B71, B710, B711, B718, B719, B72*, B73*, B74*, B75*, B76*, B77*, B78*, B79*, B80*, B81, B810, B811, B812, B813, B814, B818, B82, B820, B829, B83, B830, B831, B832, B833, B834, B838, B839, B85*, B86*, B87*, B88, B880, B881, B882, B883, B888, B889, B89*, H061, H130, J173, K231, M631, O986, P371, P373, P374 |
| Fungal infections | B35*, B36*, B37, B370, B371, B372, B373, B374, B375, B376, B377, B378, B379, B38, B380, B381, B382, B383, B384, B387, B388, B389, B39, B390, B391, B392, B393, B394, B395, B399, B40, B400, B401, B402, B403, B407, B408, B409, B41, B410, B417, B418, B419, B42, B420, B421, B427, B428, B429, B43, B430, B431, B432, B438, B439, B44*, B45, B450, B451, B452, B453, B457, B458, B459, B46, B460, B461, B462, B463, B464, B465, B468, B469, B470, B48, B480, B481, B482, B483, B484, B487, B488, B49*, B59*, G021, H622, J172, M016, P375 |
| Superficial fungal infections | B35, B350, B351, B352, B353, B354, B356, B358, B359, B36, B360, B368, B369, B372 |
| Deep fungal infections | B370, B371, B373, B374, B375, B376, B377, B378, B379, B382, B387, B388, B389, B390, B399, B408, B430, B432, B440, B441, B447, B448, B449, B450, B451, B459, B470, B487, B488, B59, G021, H622, J172 |

* Indicates inclusion of underlying 4- and 5-digit codes·

## Table S44. Classification of site-specific infectious diseases in the study on diagnosis level.

| **Infection site** | **ICD10 codes** |
| --- | --- |
| Upper respiratory tract including ear | A545, B053, B085, B974, J010, J02, J020, J028, J029, J03, J030, J038, J039, J04*, J051, J36*, J390, J391, H600, H601, H602, H603, H620, H621, H622, H623, H660, H670, H671 |
| Lower respiratory tract | A065, A15*, A16*, A202, A210, A212, A221, A420, A430, B012, B052, B221, B371, B380, B381, B382, B400, B401, B402, B410, B420, B450, B460, B583, B59*, B664, B960, B961, J12*, J13*, J14*, J15, J150, J151, J152, J153, J154, J155, J156, J157, J158, J159, J16, J160, J168, J170, J171, J172, J173, J178, J18*, J20, J200, J201, J202, J203, J204, J205, J206, J207, J208, J209, J21*, J210, J211, J218, J219, J22*, J851, J852, J853, J86*, O980, P23, P230, P231, P232, P233, P234, P235, P236, P238, P239, P370 |
| Gastrointestinal tract including liver | A051, A052, A053, A054, A058, A059, A06, A00, A000, A001, A009, A01*, A02, A020, A022, A028, A029, A03, A030, A031, A032, A033, A038, A039, A04, A040, A041, A042, A043, A044, A045, A046, A047, A048, A049, A05, A050, A064, A060, A061, A062, A063, A07*, A080, A081, A082, A083, A084, A085, A09*, A222, B054, B15, B150, B159, B16, B160, B161, B162, B169, B17*, B18*, B19, B190, B199, B462, B57*, B581, B663, B81, B810, B811, B812, B813, B814, B818, B971, B980, K35*, K570, K572, K574, K578, K61*, K630, K650, K670, K671, K672, K673, K678, K750, K770, O984, P353 |
| Skin and soft tissue | A067, A201, A220, A260, A320, A441, A431, A513, A67*, B000, B001, B07*, B088, B09*, B35*, B372, B383, B43, B430, B431, B432, B438, B452, B463, B480, B551, B552, B86*, L00*, L01*, L02*, L03*, L04*, L05*, L08*, L701, P394 |
| Bloodstream | A021, A207, A217, A218, A219, A227, A267, A327, A391, A392, A393, A394, A40, A400, A401, A402, A403, A408, A409, A41, A410, A411, A412, A413, A414, A415, A418, A419, A427, A440, A483, A484, B377, B650, B651, B652, B653, B658, B659, P36, O85*, P360, P361, P362, P363, P364, P365, P368, P369, P372, R650, R651, R788 |
| Bone joint and connective tissue | A544, B330, B453, M00, M000, M001, M002, M008, M009, M010, M011, M012, M013, M014, M015, M016, M018, M462, M463, M465, M490, M491, M492, M493, M600, M630, M631, M632, M650, M651, M710, M711, M726, M730, M731, M86*, M860, M861, M862, M863, M864, M865, M866, M868, M869 |
| Genitourinary | A510, A511, A540, A541, A542, A546, A548, A549, A55*, A560, A561, A562, A563, A564, A568, A57*, A58*, A59*, A60*, A630, A638, A64*, B260, B373, B374, N080, N10*, N136, N151, N160, N290, N291, N300, N340, N390, N410, N412, N45*, N61*, N700, N710, N730, N733, N74, N740, N741, N742, N743, N744, N748, N751, N760, N764, N770, N771, O030, O035, O040, O045, O050, O055, O060, O065, O070, O075, O080, O230, O231, O232, O233, O234, O235, O239, O753, O86*, O910, O911, O98, O981, O982, O983, O985, O986, O987, O988, O989, P39, P390, P393, P398 |
| Heart and circulation | A520, B332, B334, B376, I301, I320, I321, I330, I400, I410, I411, I412, I430, I520, I521, I681 |
| Neurological and eye | A066, A17, A170, A171, A178, A179, A211, A203, A321, A39, A390, A391, A392, A393, A394, A395, A398, A399, A503, A504, A523, A521, A522, A543, A71*, A740, A80*, A811, A812, A83*, A84*, A85*, A86*, A87*, A88*, A89*, B003, B004, B005, B010, B011, B020, B021, B022, B023, B050, B051, B060, B261, B262, B30*, B375, B384, B451, B461, B580, B582, B691, G00, G000, G001, G002, G003, G008, G009, G01*, G020, G021, G028, G03*, G041, G042, G050, G051, G052, G06*, G07*, H00*, H010, H050, H061, H100, H105, H130, H190, H191, H192, H220, H320, H440, P391 |

* Indicates inclusion of underlying 4- and 5-digit code

## Table S45 Definition, classification, and missing rates of major covariates.

| **Covariates** | **Description** | **Missing rates** |
| --- | --- | --- |
| **Age** | Age at recruitment was derived by subtracting the date of recruitment from the date of birth and UK Biobank calculated the variable truncating to the whole year. | / |
| **Sex** | It was treated as a categorical variable (“Female” and “Male”) | / |
| **Ethnicity** | The ethnic groups available for participants to choose from are: “White” (including British, Irish, any other White background), “Mixed” (including White and the Black Caribbean, White and Black African, White and Asian, any other mixed background), “Asian or Asian British” (including Indian, Pakistani, Bangladeshi, any other Asian background), “Black or Black British” (including Caribbean, African, any other Black background), “Chinese”, “Other ethnic group”, “Do not know” or “Prefer not to answer”. We treated it as a binary variable (“White” and “Others”), putting all options except “White” into the group “Others”. | 0.59% |
| **Townsend deprivation index (TDI)** | TDI was defined according to the unemployment rate, the percentage of overcrowded households, the percentage of people without cars, and the percentage of people without houses for each area in the UK, and baseline TDI calculated immediately before participant joining UK Biobank based on the preceding national census output areas. Each participant was assigned a score corresponding to the output area in which their postcode is located. We defined “Low”, “Moderate”, and “High” deprivation corresponding to tertiles of TDI and treated it as a continuous variable in survival analyses. | 0.12% |
| **Education level** | The educational qualification categories available for participants to choose from were as follows: “College or University degree,” “A levels/AS levels or equivalent,” “O levels/GCSEs or equivalent,” “CSEs or equivalent,” “NVQ or HND or HNC or equivalent,” “Other professional qualifications, e.g., nursing, teaching,” “None of the above,” and “Prefer not to answer.” Except for the first and last options, all other options are lower than college qualification. This was treated as a binary variable (“College” and “Below college”), with all options except “College or University degree” placed in the group “Below college”. | 2.29% |
| **Smoking status** | The smoking status variable originally included four options: ‘Never’, ‘Previous’, ‘Current’, and ‘Prefer not to answer’. For analysis, smoking status was treated as a categorical variable with three groups: non-smoker (‘Never’), previous smoker (‘Previous’), and current smoker (‘Current’). Missing or non-informative responses were imputed using the most frequent category. | 0.63% |
| **Alcohol consumption** | Alcohol consumption was assessed by touchscreen questionnaire asking: *“Did you have any alcoholic drinks yesterday? For instance, beer, wine, or spirits.”* Based on multiple rounds of assessment, drinking status was classified into three categories: current drinkers (those reporting alcohol consumption), previous drinkers (those who reported no drinking in the most recent round but had prior history of alcohol use), and never drinkers (those consistently reporting no alcohol consumption). | 0.36% |
| **Physical activity level** | Categorical variable, regular and irregular.  UK Biobank Touchscreen questionnaire on the reported type and duration of physical activity (including walking, DIY, moderate and vigorous physical activity, strenuous sports, etc).  One of the following is equal to 1 score:  1.≥150 minutes of moderate activity per week.  2.≥ 75 minutes of vigorous activity per week.  3. Equivalent combination.  4. Moderate physical activity at least 5 days a week or vigorous activity once a week.  1 score and more indicated regular physical activity. | / |
| **Body mass index (BMI)** | BMI was constructed from weight and height immediately after physical measurement at the assessment center. We treated it as a continuous variable. | 0.63% |
| **INFLA score** | The INFLA score includes C-reactive protein, white blood cell, platelet count, and the neutrophil granulocyte to lymphocyte ratio (G/L ratio). It is a biomarker of low-grade inflammation. For each of the biomarker levels, ten-tiles were created. In this system, the highest deciles (7th to 10th) were assigned positive scores increasing from 1 to 4. Conversely, the lowest deciles (1st to 4th) received negative scores decreasing from -4 to -1. If a biomarker level fell into the 5th or 6th decile, it was given a score of zero. This method resulted in an INFLA-score that varied from -16 to 16, calculated as the aggregate of the scores from these four biomarkers. A higher INFLA score indicates increased low-grade inflammation. | 9.41% |
| **Charlson Comorbidity Index (CCI)** | CCI is used to predict mortality by classifying or weighting comorbidities (co-existing medical conditions). Each comorbidity category has an associated weight, based on the adjusted risk of mortality or resource use, and the sum of all the weights results in a single comorbidity score for a patient. The higher the score, the more likely the predicted negative outcome (like death) will occur. This index is widely used in health research to adjust for the severity of comorbid conditions in risk adjustment models. | / |

## Table S46. Comprehensive IBD-related protein panel integrating inflammatory, therapeutic, and barrier-fibrosis pathways.

| **Classify** | **UKB ID** | **Gene** | **Protein name** |
| --- | --- | --- | --- |
| Classical inflammatory pathway | 2712 | TNF | Tumor necrosis factor |
|  | 1386 | IL1B | Interleukin-1 beta |
|  | 1418 | IL6 | Interleukin-6 |
|  | 750 | CXCL8 | Interleukin-8 |
|  | 388 | CCL2 | C-C motif chemokine 2 |
|  | 1853 | NFKB1 | Nuclear factor NF-kappa-B p105 subunit |
|  | 2696 | TLR4 | Toll-like receptor 4 |
|  | 2399 | SERPINA3 | Alpha-1-antichymotrypsin |
| Anti-TNF biologics–related pathway | 2712 | TNF | Tumor necrosis factor |
|  | 2727 | TNFRSF1A | Tumor necrosis factor receptor superfamily member 1A |
|  | 2728 | TNFRSF1B | Tumor necrosis factor receptor superfamily member 1B |
| Anti-IL-12/23 biologics–related pathway | 1365 | IL12B | Interleukin-12 subunit beta |
|  | 1366 | IL12RB1 | Interleukin-12 receptor subunit beta-1 |
| Anti-integrin biologics–related pathway | 1463 | ITGB7 | Integrin beta-7 |
|  | 1314 | ICAM1 | Intercellular adhesion molecule 1 |
|  | 1453 | ITGAL | Integrin alpha-L |
|  | 1454 | ITGAM | Integrin alpha-M |
|  | 1460 | ITGB2 | Integrin beta-2 |
| JAK inhibitor–related pathway | 2574 | STAT2 | Signal transducer and activator of transcription 2 |
|  | 2575 | STAT5B | Signal transducer and activator of transcription 5B |
|  | 93 | AKT2 | RAC-beta serine/threonine-protein kinase |
| Anti-IL-17 biologics–related pathway | 1374 | IL17A | Interleukin-17A |
|  | 1378 | IL17RA | Interleukin-17 receptor A |
| S1P receptor modulator–related pathway | 388 | CCL2 | C-C motif chemokine 2 |
|  | 750 | CXCL8 | Interleukin-8 |
| Oxidative stress–related protein markers | 2521 | SOD2 | Superoxide dismutase [Mn], mitochondrial |
|  | 367 | CAT | Catalase |
|  | 2133 | PRDX3 | Thioredoxin-dependent peroxide reductase, mitochondrial |
|  | 1281 | HMOX1 | Heme oxygenase 1 |
|  | 1226 | GSR | Glutathione reductase, mitochondrial |
|  | 1230 | GSTP1 | Glutathione S-transferase P |
|  | 2520 | SOD1 | Superoxide dismutase [Cu-Zn] |
| ntestinal barrier function–related protein markers | 1930 | OCLN | Occludin |
|  | 484 | CDH1 | Cadherin-1 |
|  | 1781 | MUC2 | Mucin-2 |
|  | 2654 | TFF3 | Trefoil factor 3 |
|  | 2048 | PIGR | Polymeric immunoglobulin receptor |
|  | 2691 | TJP3 | Tight junction protein ZO-3 |
|  | 1645 | LYPD8 | Ly6/PLAUR domain-containing protein 8 |
| Intestinal fibrosis core protein markers | 629 | COL1A1 | Collagen alpha-1(I) chain |
|  | 633 | COL3A1 | Collagen alpha-1(III) chain |
|  | 1731 | MMP1 | Interstitial collagenase |
|  | 2685 | TIMP1 | Metalloproteinase inhibitor 1 |
|  | 2660 | TGFB1 | Transforming growth factor beta-1 proprotein |
|  | 248 | BCL2L11 | Bcl-2-like protein 11, Isoform BimL |
|  | 240 | BAX | Apoptosis regulator BAX |
|  | 271 | BMPER | BMP-binding endothelial regulator protein |
|  | 1071 | FN1 | Fibronectin |
|  | 246 | BCL2 | Apoptosis regulator Bcl-2 |
|  | 248 | BCL2L11 | Bcl-2-like protein 11, Isoform BimL |
|  | 240 | BAX | Apoptosis regulator BAX |

## Table S47. Definition and coding of antibiotic use.

| **Type** | **Coding** |
| --- | --- |
| Antibiotic | 1140853180,1140853184,1140853186,1140853188,1140853262,1140853270,1140853272,1140853274,1140853276,1140853278,1140853282,1140853342,1140853344,1140853370,1140853484,1140853488,1140853790,1140853854,1140853862,1140853910,1140856602,1140856604,1140856606,1140856608,1140856610,1140856612,1140856618,1140856620,1140856622,1140856636,1140856638,1140856640,1140856642,1140856644,1140856646,1140856690,1140856692,1140856694,1140856696,1140856704,1140856706,1140856708,1140856710,1140856712,1140856714,1140856716,1140856718,1140856722,1140856724,1140856726,1140856728,1140856730,1140856732,1140856734,1140856736,1140856740,1140856742,1140856744,1140856746,1140856748,1140856750,1140856752,1140856754,1140856756,1140856758,1140856760,1140856762,1140856772,1140856774,1140856778,1140856780,1140856784,1140856786,1140856788,1140856790,1140856792,1140856796,1140856802,1140856804,1140856806,1140856808,1140856812,1140856814,1140856820,1140856822,1140856824,1140856826,1140856832,1140856834,1140856836,1140856838,1140856858,1140856860,1140856862,1140856864,1140856868,1140856870,1140856878,1140856880,1140856882,1140856890,1140856898,1140856900,1140856902,1140856904,1140856906,1140856908,1140856916,1140856918,1140856926,1140856928,1140856932,1140856934,1140856940,1140856942,1140856944,1140856952,1140856954,1140856960,1140856964,1140856966,1140856974,1140856976,1140856980,1140856984,1140856990,1140856992,1140857000,1140857006,1140857008,1140857010,1140857012,1140857014,1140857016,1140857022,1140857024,1140857032,1140857036,1140857038,1140857044,1140857050,1140857052,1140857054,1140857056,1140857060,1140857062,1140857064,1140857092,1140857094,1140857096,1140857106,1140857108,1140857110,1140857112,1140857114,1140857120,1140857124,1140857126,1140857128,1140857134,1140857138,1140857140,1140857146,1140857150,1140857152,1140857160,1140857162,1140857166,1140857180,1140857182,1140857184,1140857186,1140857188,1140857190,1140857192,1140857194,1140857196,1140857198,1140857200,1140857202,1140857206,1140857208,1140857232,1140857234,1140857238,1140857240,1140857288,1140857294,1140857296,1140857298,1140857300,1140857302,1140857304,1140857306,1140857308,1140857310,1140857326,1140857330,1140857336,1140857346,1140857348,1140859058,1140859068,1140859070,1140859148,1140859150,1140859208,1140859220,1140863512,1140863598,1140863600,1140864180,1140864352,1140864362,1140864366,1140864368,1140864530,1140864584,1140864712,1140864912,1140869142,1140869240,1140869242,1140872584,1140872586,1140872590,1140872596,1140872598,1140872600,1140872602,1140872604,1140872606,1140872608,1140872610,1140872612,1140872614,1140872616,1140872622,1140872624,1140872632,1140872634,1140872638,1140872642,1140872646,1140872650,1140872652,1140872656,1140872658,1140872660,1140872662,1140872664,1140872666,1140872668,1140872674,1140872676,1140872678,1140872680,1140872682,1140872694,1140872708,1140872710,1140872712,1140872714,1140872716,1140872718,1140872720,1140872722,1140872724,1140872728,1140872730,1140872732,1140872734,1140872748,1140872758,1140872760,1140872762,1140872764,1140872766,1140872768,1140872770,1140872772,1140872776,1140872778,1140872780,1140872782,1140872784,1140872786,1140872788,1140872790,1140872792,1140872794,1140872796,1140872798,1140872800,1140872802,1140872804,1140872806,1140872808,1140872810,1140872812,1140872814,1140872816,1140872818,1140872820,1140872822,1140872824,1140872826,1140872838,1140872840,1140872844,1140872846,1140872848,1140872850,1140872852,1140872854,1140872856,1140872858,1140872860,1140872862,1140872864,1140872866,1140872882,1140872884,1140872886,1140872888,1140872896,1140872898,1140872900,1140872902,1140872904,1140872906,1140872908,1140872910,1140872912,1140872914,1140872916,1140872918,1140872920,1140872922,1140872924,1140872934,1140872936,1140872952,1140872960,1140872962,1140872964,1140872966,1140872968,1140872970,1140872972,1140872974,1140872976,1140872978,1140872982,1140872984,1140872986,1140872994,1140872998,1140873000,1140873002,1140873004,1140873006,1140873008,1140873020,1140873022,1140873024,1140873026,1140873066,1140873068,1140873070,1140873072,1140873074,1140873090,1140873094,1140873098,1140873100,1140873102,1140873104,1140873106,1140873108,1140873110,1140873124,1140873126,1140873128,1140873130,1140873138,1140873140,1140873142,1140873144,1140873146,1140873148,1140873154,1140873156,1140873168,1140873170,1140873172,1140873174,1140873182,1140873184,1140873186,1140873194,1140873196,1140873202,1140873204,1140873206,1140873208,1140873210,1140873216,1140873220,1140873224,1140873242,1140873244,1140873248,1140873250,1140873252,1140873258,1140873260,1140873262,1140873264,1140873266,1140873268,1140873280,1140873282,1140873284,1140873286,1140873288,1140873304,1140873306,1140873308,1140873310,1140873312,1140873314,1140873316,1140873318,1140873320,1140873322,1140873324,1140873326,1140873328,1140873330,1140873332,1140873334,1140873336,1140873350,1140873356,1140873358,1140873360,1140873364,1140873366,1140873368,1140873370,1140873372,1140873374,1140873376,1140873384,1140873390,1140873394,1140873396,1140873398,1140873400,1140873402,1140873404,1140873408,1140873410,1140873412,1140873416,1140873422,1140873424,1140873428,1140873430,1140873434,1140873436,1140873438,1140873440,1140873450,1140873456,1140873458,1140873460,1140873462,1140873464,1140873466,1140873474,1140873476,1140873480,1140873482,1140873484,1140873488,1140873490,1140873496,1140873498,1140873506,1140873508,1140873512,1140873514,1140873516,1140873518,1140873520,1140873522,1140873524,1140873536,1140873538,1140873540,1140873542,1140873548,1140873554,1140873556,1140873558,1140873560,1140873562,1140873564,1140873568,1140873570,1140873574,1140873582,1140873586,1140873588,1140873590,1140873592,1140873608,1140873618,1140873620,1140873622,1140873630,1140873632,1140873634,1140873640,1140873642,1140873648,1140873650,1140873652,1140873654,1140873656,1140873658,1140873660,1140873672,1140873674,1140873676,1140873678,1140873680,1140873682,1140873690,1140873692,1140873694,1140873696,1140873698,1140873700,1140873702,1140873708,1140873712,1140873714,1140873718,1140873722,1140873730,1140873732,1140873738,1140873740,1140873742,1140873744,1140873746,1140873748,1140873750,1140873752,1140873754,1140873756,1140873768,1140873774,1140873776,1140873780,1140873798,1140873810,1140873812,1140873814,1140873816,1140873818,1140873820,1140873822,1140873824,1140873826,1140873828,1140873830,1140873832,1140873834,1140873842,1140873844,1140873846,1140873850,1140873856,1140873858,1140873860,1140873862,1140873864,1140873866,1140873868,1140873870,1140873872,1140873874,1140873876,1140873878,1140873880,1140873882,1140873884,1140873886,1140873888,1140873890,1140873892,1140873894,1140873896,1140873898,1140873928,1140873930,1140873932,1140873934,1140873936,1140873938,1140873940,1140873942,1140873944,1140873946,1140873948,1140873950,1140873952,1140873956,1140873962,1140873966,1140873976,1140873978,1140874008,1140874014,1140874030,1140874032,1140874034,1140874036,1140874038,1140874040,1140874042,1140874044,1140874046,1140874048,1140874050,1140874052,1140874054,1140874098,1140874112,1140874114,1140874116,1140874118,1140874120,1140874126,1140874138,1140874140,1140874142,1140874144,1140874146,1140874148,1140874160,1140874164,1140874166,1140874170,1140874174,1140874178,1140874184,1140874186,1140874188,1140874190,1140874192,1140874194,1140874196,1140874198,1140874208,1140874210,1140874214,1140874216,1140874220,1140874226,1140874228,1140874230,1140874232,1140875558,1140875560,1140875562,1140875566,1140875568,1140875576,1140875580,1140875582,1140875650,1140875652,1140875658,1140875660,1140875662,1140875664,1140875666,1140875730,1140875736,1140875740,1140875746,1140875968,1140875970,1140876036,1140876044,1140876046,1140878520,1140878526,1140878528,1140880068,1140880140,1140880142,1140880146,1140880200,1140880202,1140880276,1140880336,1140880338,1140880340,1140880342,1140880350,1140880352,1140880354,1140880356,1140880360,1140880366,1140880378,1140880462,1140882324,1140882326,1140882328,1140882330,1140882332,1140882336,1140882340,1140882344,1140882352,1140882354,1140882356,1140882358,1140882360,1140882364,1140882366,1140882374,1140882416,1140882418,1140882420,1140882422,1140882426,1140882432,1140882458,1140882460,1140882498,1140882506,1140882514,1140882516,1140882520,1140882522,1140882530,1140882532,1140882534,1140882536,1140882538,1140882542,1140882544,1140882546,1140882560,1140882564,1140882892,1140883982,1140883984,1140883986,1140883988,1140883998,1140884002,1140884006,1140884008,1140884012,1140884016,1140884022,1140884042,1140884046,1140884048,1140884050,1140884052,1140884054,1140884064,1140884066,1140884068,1140884070,1140884072,1140884074,1140884076,1140884082,1140884086,1140884090,1140884098,1140884102,1140884114,1140884118,1140884142,1140884168,1140884170,1140884176,1140884178,1140884186,1140884190,1140884194,1140884198,1140884202,1140884206,1140888718,1140888720,1140888758,1140909416,1140909454,1140909504,1140909542,1140909544,1140909656,1140909658,1140909682,1140909688,1140909690,1140909698,1140909716,1140909718,1140909746,1140909748,1140910046,1140910360,1140910444,1140910558,1140910610,1140910816,1140910894,1140911714,1140911716,1140911718,1140911720,1140911838,1140911840,1140912210,1140912778,1140912910,1140912912,1140912914,1140912994,1140912998,1140913038,1140916292,1140916294,1140916608,1140916654,1140916656,1140916846,1140917210,1140917212,1140917214,1140917216,1140917218,1140917220,1140917228,1140917236,1140917238,1140917240,1140917458,1140917474,1140921786,1140921790,1140921792,1140921842,1140922030,1140922048,1140922052,1140922556,1140923472,1140923614,1140923616,1140923750,1140923752,1140926570,1140926572,1140926574,1140926576,1140926578,1140926868,1140926870,1140926968,1140926970,1140928674,1140928848,1140928850,1140928852,1141145800,1141146054,1141146112,1141146120,1141147308,1141150930,1141150932,1141151346,1141151424,1141151884,1141151886,1141152062,1141152068,1141152070,1141152128,1141152130,1141152132,1141152134,1141152200,1141152202,1141152204,1141152206,1141152208,1141152210,1141152218,1141152520,1141152966,1141153228,1141153292,1141157224,1141157230,1141157232,1141157340,1141157342,1141157344,1141157346,1141157348,1141157350,1141157352,1141157354,1141157356,1141157358,1141157364,1141157366,1141157396,1141157438,1141157498,1141162668,1141162676,1141162678,1141162680,1141163532,1141163716,1141163778,1141163784,1141163860,1141164834,1141166116,1141166128,1141166132,1141166498,1141166502,1141166930,1141166932,1141166956,1141166960,1141167038,1141167508,1141167608,1141167610,1141168270,1141168330,1141168332,1141168334,1141168372,1141169416,1141169418,1141170186,1141170534,1141171002,1141171562,1141171612,1141171614,1141172120,1141172954,1141172958,1141172970,1141173698,1141173700,1141173702,1141173760,1141175914,1141179760,1141179928,1141180226,1141180228,1141180368,1141180392,1141180450,1141180644,1141181004,1141181008,1141182110,1141182122,1141182124,1141182178,1141182666,1141182668,1141183258,1141184388,1141185430,1141185434,1141186826,1141186830,1141187100,1141187104,1141187280,1141187764,1141187768,1141187770,1141188762,1141188766,1141190776,1141191010,1141192042,1141192154,1141192340,1141193808,1141193810,1141195072,1141195074,1141195112,1141195580,1141195582,1141200706 |

**Reference:**

1. Shrestha S, Olén O, Eriksson C, Everhov ÅH, Myrelid P, Visuri I, Ludvigsson JF, Schoultz I, Montgomery S, Sachs MC, Halfvarson J; SWIBREG Study Group; Olsson M, Hjortswang H, Bengtsson J, Strid H, Andersson M, Jäghult S, Eberhardson M, Nordenvall C, Björk J, Fagerberg UL, Rejler M, Grip O, Karling P, Block M, Angenete E, Hellström PM, Gustavsson A. The use of ICD codes to identify IBD subtypes and phenotypes of the Montreal classification in the Swedish National Patient Register. Scand J Gastroenterol. 2020 Apr;55(4):430-435.

2.Sipilä PN, Heikkilä N, Lindbohm JV, Hakulinen C, Vahtera J, Elovainio M, Suominen S, Väänänen A, Koskinen A, Nyberg ST: Hospital-treated infectious diseases and the risk of dementia: a large, multicohort, observational study with a replication cohort. The Lancet Infectious Diseases 2021, 21(11):1557-1567.

3.Zheng J, Ni C, Lee SR, Li FR, Huang J, Zhou R, Huang Y, Lip GY, Wu X, Tang S: Association of hospital‐treated infectious diseases and infection burden with cardiovascular diseases and life expectancy. Journal of Internal Medicine 2024, 295(5):679-694.

4. Jess T, Simonsen J, Nielsen NM, Jørgensen KT, Bager P, Ethelberg S, Frisch M. Enteric Salmonella or Campylobacter infections and the risk of inflammatory bowel disease. Gut. 2011 Mar;60(3):318-24.

5. Monaghan TM, Cockayne A, Mahida YR. Pathogenesis of Clostridium difficile Infection and Its Potential Role in Inflammatory Bowel Disease. Inflamm Bowel Dis. 2015 Aug;21(8):1957-66

6. Sun Y, Yuan S, Chen X, Sun J, Kalla R, Yu L, Wang L, Zhou X, Kong X, Hesketh T, Ho GT, Ding K, Dunlop M, Larsson SC, Satsangi J, Chen J, Wang X, Li X, Theodoratou E, Giovannucci EL. The Contribution of Genetic Risk and Lifestyle Factors in the Development of Adult-Onset Inflammatory Bowel Disease: A Prospective Cohort Study. Am J Gastroenterol. 2023, 118(3):511-522.

7. Liu JZ, van Sommeren S, Huang H, Ng SC, Alberts R, Takahashi A, Ripke S, Lee JC, Jostins L, Shah T, Abedian S, Cheon JH, Cho J, Dayani NE, Franke L, Fuyuno Y, Hart A, Juyal RC, Juyal G, Kim WH, Morris AP, Poustchi H, Newman WG, Midha V, Orchard TR, Vahedi H, Sood A, Sung JY, Malekzadeh R, Westra HJ, Yamazaki K, Yang SK; International Multiple Sclerosis Genetics Consortium; International IBD Genetics Consortium; Barrett JC, Alizadeh BZ, Parkes M, Bk T, Daly MJ, Kubo M, Anderson CA, Weersma RK. Association analyses identify 38 susceptibility loci for inflammatory bowel disease and highlight shared genetic risk across populations. Nat Genet. 2015, 47(9):979-986.

8. Liu Z, Liu R, Gao H, Jung S, Gao X, Sun R, Liu X, Kim Y, Lee HS, Kawai Y, Nagasaki M, Umeno J, Tokunaga K, Kinouchi Y, Masamune A, Shi W, Shen C, Guo Z, Yuan K; FinnGen; International Inflammatory Bowel Disease Genetics Consortium; Chinese Inflammatory Bowel Disease Genetics Consortium; Zhu S, Li D, Liu J, Ge T, Cho J, Daly MJ, McGovern DPB, Ye BD, Song K, Kakuta Y, Li M, Huang H. Genetic architecture of the inflammatory bowel diseases across East Asian and European ancestries. Nat Genet. 2023;55(5):796-806.

9. Shi H, Schweren LJS, Ter Horst R, Bloemendaal M, van Rooij D, Vasquez AA, Hartman CA, Buitelaar JK. Low-grade inflammation as mediator between diet and behavioral disinhibition: A UK Biobank study. Brain Behav Immun. 2022 Nov;106:100-110.
